# Nanoarchitectured prototypes of mesoporous silica nanoparticles for innovative biomedical applications

**DOI:** 10.1186/s12951-022-01315-x

**Published:** 2022-03-12

**Authors:** Ranjith Kumar Kankala, Ya-Hui Han, Hong-Ying Xia, Shi-Bin Wang, Ai-Zheng Chen

**Affiliations:** 1 Institute of Biomaterials and Tissue Engineering, Huaqiao University, Xiamen, 361021 Fujian People’s Republic of China; 2 College of Chemical Engineering, Huaqiao University, Xiamen, 361021 Fujian People’s Republic of China; 3 Fujian Provincial Key Laboratory of Biochemical Technology (Huaqiao University), Xiamen, 361021 Fujian People’s Republic of China

**Keywords:** Surface immobilization, Metal-impregnation, Degradability, Biocompatibility, Drug delivery, Tissue engineering, Immune therapy

## Abstract

Despite exceptional morphological and physicochemical attributes, mesoporous silica nanoparticles (MSNs) are often employed as carriers or vectors. Moreover, these conventional MSNs often suffer from various limitations in biomedicine, such as reduced drug encapsulation efficacy, deprived compatibility, and poor degradability, resulting in poor therapeutic outcomes. To address these limitations, several modifications have been corroborated to fabricating hierarchically-engineered MSNs in terms of tuning the pore sizes, modifying the surfaces, and engineering of siliceous networks. Interestingly, the further advancements of engineered MSNs lead to the generation of highly complex and nature-mimicking structures, such as Janus-type, multi-podal, and flower-like architectures, as well as streamlined tadpole-like nanomotors. In this review, we present explicit discussions relevant to these advanced hierarchical architectures in different fields of biomedicine, including drug delivery, bioimaging, tissue engineering, and miscellaneous applications, such as photoluminescence, artificial enzymes, peptide enrichment, DNA detection, and biosensing, among others. Initially, we give a brief overview of diverse, innovative stimuli-responsive (pH, light, ultrasound, and thermos)- and targeted drug delivery strategies, along with discussions on recent advancements in cancer immune therapy and applicability of advanced MSNs in other ailments related to cardiac, vascular, and nervous systems, as well as diabetes. Then, we provide initiatives taken so far in clinical translation of various silica-based materials and their scope towards clinical translation. Finally, we summarize the review with interesting perspectives on lessons learned in exploring the biomedical applications of advanced MSNs and further requirements to be explored.

**Graphical Abstract:**

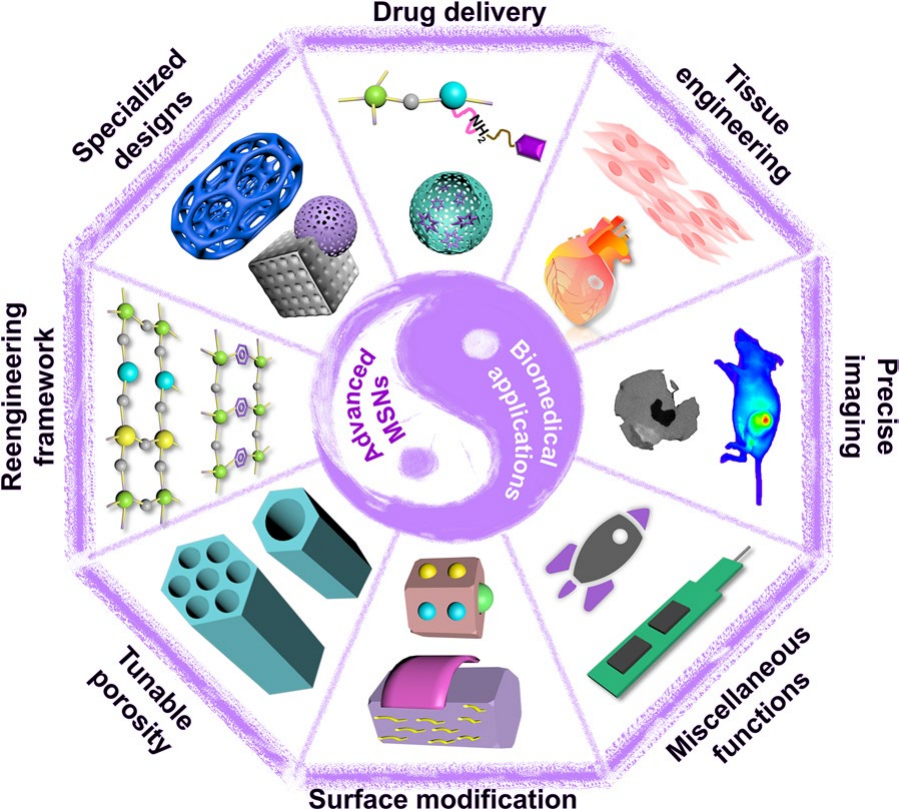

## Introduction

Since the mid-1950s, immense progress has evidenced the development of various nanoplatforms (size of 1 to 100 nm) with intrinsic functionalities for diverse applications, such as agriculture, adsorption, engineering, medicine, energy, and environment-related fields [1]. These nano-sized particulate forms offer unique advantages such as scalability, ease of synthesis, tunable physicochemical features (electronic, mechanical, magnetic, and optical), and unique morphological attributes, distinctively varied from their bulk counterparts [2, 3]. In addition, the predominant importance is gained from researchers due to prominent features of great surface chemistry and large surface-to-volume ratio, facilitating the encapsulation of various guest species [4, 5]. Owing to different precursors, several kinds of inorganic-based materials have been fabricated, including but not limited to silver (Ag), gold (Au), platinum (Pt), calcium phosphates (hydroxyapatite), titanium, black phosphorus (BP) based-quantum dots (QDs), cadmium-conjugated selenium, and tellurium-based QDs, layered double hydroxides (LDHs), palladium, silica, rhodium, and zinc nanoconstructs [1, 3, 6–8]. In medicine, these nanocontainers encapsulated with certain therapeutic guest species alter their biobehavior by overcoming various biological barriers and delivering them to the target site to improve the therapeutic efficacy both in-vitro and in-vivo.[9] In addition to highly advantageous physicochemical and morphological attributes, the tailoring convenience and functionalization of surfaces substantially improve their intrinsic properties of compatibility, versatility, and stability (thermal/colloidal) towards potential biomedical applications, such as biosensing, drug delivery, peptide enrichment, artificial enzymes, photoluminescence, bioimaging, and tissue engineering [8, 10–14]. Recently, several advancements towards fabricating innovative therapeutic platforms have been evidenced using these diverse constructs and their composites with other species, resulting in the magnetic- [15, 16], ultrasound- [17, 18], and photo- [18–20] responsive materials for synergistic therapeutic effects [9].

Among diverse inorganic-based nanomaterials available, silica has gathered enormous attentiveness in medicine owing to its considerable compatibility. Moreover, several facts of endogenous availability in bone and as an excipient in oral formulations have rendered the silica-based materials safe by the United States Food and Drug Administration (US-FDA) for medicine. The versatile mesoporous silica nanoparticles (MSNs) present highly advantageous physicochemical features and attractive morphological attributes, such as particle sizes of 30–200 nm, the surface area of ~ 1500 m^2^/g, tunable mesopores of 2–10 nm, and arbitrary sizes, as well as shapes (spheres, fibers, gyroids, and tubules). Such impressive attributes are of particular interest in adsorption [21], catalysis [22], optical devices [23], polymeric fillers [24], and biomedicine-related applications [25, 26]. Specifically, several other attractive features of exceptional hydrophilic surface topology and porous interior, facilitate ease of surface functionalization, colloidal stability, and high dispersity. These advantageous characteristics have enabled their applicability in various biomedical applications, such as bio-imaging [27], biosensing [18], biocatalysts [28], tissue engineering [29], and therapeutic cargo (drug/protein/gene) delivery [26, 30–32]. Compared to various organic-based nanoformulations, these inorganic silica matrices offer higher efficacy in conveying the therapeutic cargo due to the porous architectures and exceptional colloidal and thermal stabilities. Accordingly, the conventional MSNs have been extensively investigated both in-vitro and in-vivo to explore the performance efficacy and safety attributes. Nonetheless, some of the performance attributes, such as long-term circulation and distribution efficacy, fail to result in satisfactory results due to a complex biological environment, hindering their applicability [33]. While understanding the complexity and exploring to solve these inherent problems of traditional MSNs, several advancements have been evidenced in the fabrication of various advanced prototypes of MSNs [34–36]. Considering the characteristics of advantageous tunable siliceous frameworks and highly reactive surface hydroxyl groups, it is highly convenient to fabricate advanced MSNs by harnessing their physicochemical and morphological properties, in terms of modifying the surface with the supramolecular networks, impregnating molecular species (metals), and improvising the porosity towards augmenting their applicability for innovative biomedical applications [9, 37, 38]. Notably, these advancements have explored the versatility of MSNs, guiding their chances in the progression to their translation to address the therapeutic needs.

**Fig. 1:**
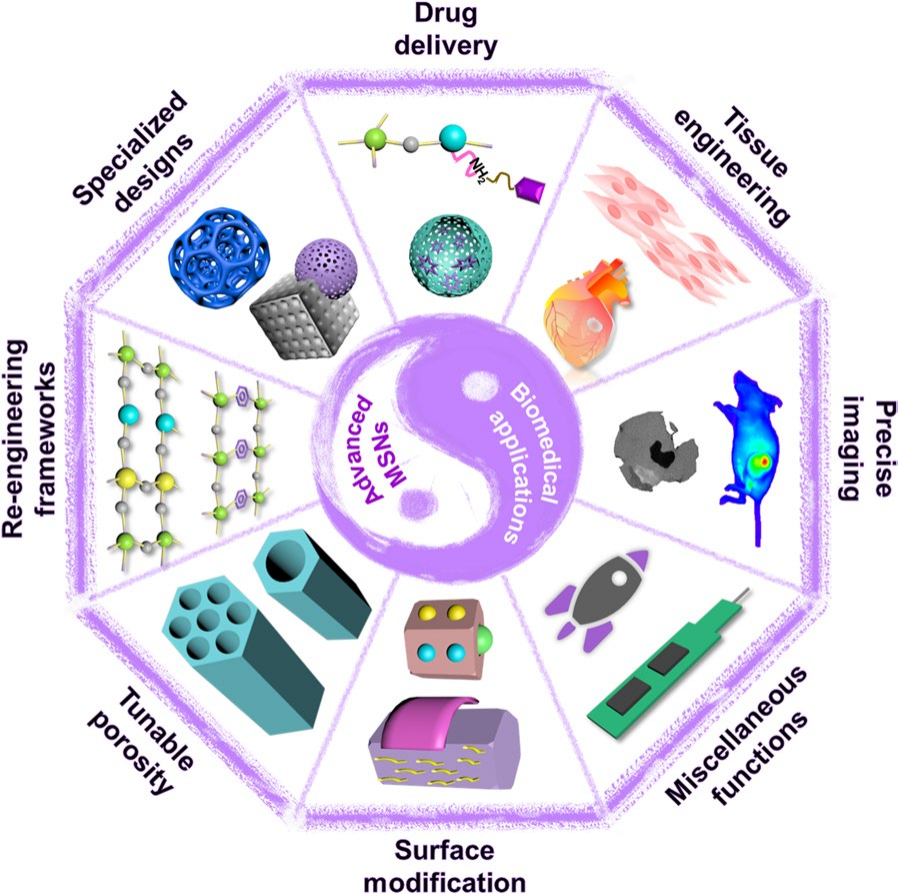
Schematic illustrating the different advanced prototypes of MSNs for varied biomedical applications

Although several reviews based on MSNs have been highlighted over the past two decades [9, 23, 25, 26, 29–31, 33, 37, 39–49], this article substantially differs with them in emphasizing the critical notified advancements of the MSNs, highlighting their biomedical applicability in diverse biomedical applications reported in the past 5 years and exploration to clinics (Fig. 1). In most of the instances, the published reviews from us and others are focused on either of the aspects of advancements, for instance, polymer coating/surface modification [50], or capping [33, 51], or framework modification [52], or discussions restricted to stimuli-responsive delivery [53], and one of the specific biomedical applications of cancer therapy [26, 31, 54], tissue engineering [29], as well as bioimaging [55]. To be precise, in our previous review on advanced MSNs, we were intended to explore the advanced prototypes of MSNs, in which the discussions were predominantly focused only on the physicochemical features and morphological attributes after modifying the MSNs with different ways of surface modification, pore alteration, and molecular impregnation in MSN frameworks [43]. In another review, we have discussed the precise encapsulation of various metallic constituents in MSNs at different positions for diverse applications of catalysis, adsorption, and medicine [42]. In another instance, we have demonstrated the modification of the framework alone with diverse molecular species towards improving the applicability [52]. Although the context of these published review articles is based on medicine, the biomedical applicability of these advanced MSNs was not well explored, in which the discussions were constricted to the modifications and their effects on the physicochemical and morphological attributes. In this article, we are intended to provide the detailed insights of various advanced prototypes of MSNs in different biomedical applications in the past few years, highlighting the effects of altered modifications in drug delivery, bioimaging, tissue engineering, and miscellaneous applications (DNA detection, artificial enzymes, peptide enrichment, and photoluminescence). In addition, recent advances in the past 2 years reported after the previous article[43] are also emphasized, for instance, cancer immune therapeutic advances and streamlined MSNs (tadpole-like) with tunable curvature. Moreover, we have provided the fundamentals and critical properties of these designed advanced prototypes of MSNs in medicine for their exploration to clinical translation.

In the further sections, we briefly introduce various types of MSNs and detailed fabrication processes to provide insights, emphasizing the factors influencing the MSNs formation and mechanisms involving reaction kinetics. Further, the advanced prototypes of MSNs, such as modified surfaces, engineered frameworks, and altered porous architectures, as well as the highlight complex Janus-type and nature-mimicking architectures, are discussed for better insight. Then, we comprehensively illustrate various biomedical applications of these advanced MSNs, with insights on drug delivery, exploring the stimuli-responsiveness (pH/light/thermos/ultrasound), targeted delivery, as well as cancer immune therapy, and various other ailments, such as diabetes, cardiac-, vascular-, and central nervous systems. Further, explicit discussions on other important biomedical applications of these advanced MSNs are provided, such as biosensing, tissue engineering/wound healing, peptide enrichment, bioimaging, photoluminescence, artificial enzymes, and deoxyribose nucleic acid (DNA) extraction, among others, opting a set of examples with critical advancements in recent times. Finally, we provide the summary with exciting perspectives, emphasizing the lessons learned so far in applying these advanced MSNs and future opportunities and challenges in their translation to clinics.

## Types and fabrication strategies

**Fig. 2:**
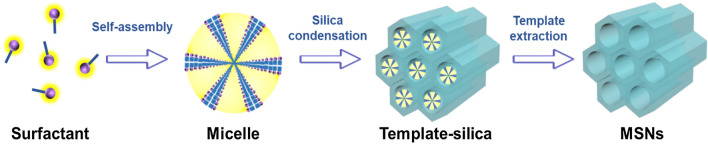
Schematic illustrating the sequential steps towards the fabrication of conventional MSNs

In the early 1990s, mesoporous silica-based hierarchical crystalline molecular sieves were first testified by Kresge et al. [56], naming them as Mobil Composition of Matter (MCM)-41. These molecular sieves were fabricated by combining the amphiphilic surfactant with silica precursor, resulting in ordered hexagonal mesophases (15–10 nm) in high yields [40]. Further, several prototypes with altered physicochemical properties have been fabricated by changing the reaction conditions and precursors, the silica source, and the surfactant templates, such as MCM-based materials (MCM-48 and MCM-50), Santa Barbara Amorphous (SBA)-type materials-1, 15, and 16, using the amphiphilic triblock copolymers as structure-directing agents [40], as well as Institute of Bioengineering and Nanotechnology (IBN-X, 2–5) using the fluorocarbons as structure-directing agents and trimethyl benzene (TMB) as a swelling agent [32, 33, 57, 58]. Comparatively, these advanced types with improved porosity are often preferred in catalysis due to significant thermal and mechanical stabilities [9, 23, 40, 59]. The synthesis of the ordered mesoporous silica species is generally based on templating method, utilizing the tetraethoxysilane (TEOS) as the silica source and amphiphilic surfactants as structure-directing templates (for instance, cetyltrimethylammonium bromide, CTAB) (Fig. 2). Although no convincing principles have been established, it is convenient to draw the MSN formation mechanism using the typical particle generation principles. In a surfactant-templating strategy, the dissolved surfactant molecules are initially arranged as micelles at a critical micelle concentration (CMC) in an alkaline medium. Further, the added silica gets deposited over the micelles through precise electrostatic interactions between the inorganic and organic constituents, resulting in their nucleation and subsequent condensation to uniform-sized MSN-based structures. Notably, the critical assessment of these two steps of nucleation and growth is often required to control the size and overall morphology of MSNs, focusing on the thermodynamics guiding the assembly of silica and surfactant, as well as the controlled reaction kinetics.

Notably, the precise control over the interfacial tensions during the self-assembly of surfactant micelles and subsequent silica condensation rates lead to controllable particle size and shapes, as well as tunable pore sizes [60]. Owing to these facts, it is appropriate to construct various mesostructures ranging from disordered structures to ordered lamellar architectures. However, optimizing reaction conditions is often necessary for the precise synthesis of uniform-sized mesoporous nanoarchitectures [23, 60]. Although the excellent topological and morphological attributes of the MSNs offer desirable properties, these features could be well-regulated by altering various factors of the synthesis conditions, i.e., formulation (surfactant, silica, and solvent) and processing/reaction (pH, temperature, and stirring speed) [23, 40, 61]. Notably, the choice of suitable surfactant and its concentration play critical roles in resulting in the ordered mesoporous arrays upon removal. The changes in the surfactant species would influence the particle size and pore dimensions. In addition to CTAB to establish the supposed interactions with the silica during its condensation, other cationic surfactants have been applied, such as cetyltrimethylammonium chloride (CTAC) or cetyltrimethoxysilane (CTMS). Despite the success in forming ordered mesoporous arrays, the CTAB surfactant would result in the MSNs with a diameter over 50 nm. To further reduce the particle size, CTAC species have been applied, which, however, resulted in disordered pore arrays. In addition to these facile surfactants, complex species, such as Pluronic copolymer and fluorocarbon surfactants with altered hydrophobicity, have been applied, resulting in the 3D cubic large porous architectures (5–30 nm) [57]. Further, several soft- and hard-templating strategies of polymers and metals as the surfactant templates have resulted in different innovative architectures, such as hollow architectures and nanorods. In addition, binary surfactants with different Mol. Wt., have been utilized, such as CTAB with (polystyrene-b-poly(acrylic acid), PS-b-PAA) to generate dual-mesoporous core–shell structures [62]. In addition to surfactants, the utilization of several external additives, swelling agents, or pore-expanders (for instance, TMB and octane) results in MSNs with enlarged pores towards improving the encapsulation of large-sized biomolecules, such as proteins [63–65]. The concentration of surfactant species plays a significant role, as in some instances, the changes in the concentration lead to irreversible aggregation and subsequent large-particle sizes with irregular pores. In addition to the most-commonly applied precursor, TEOS, TMOS can be replaced to synthesize MSNs. According to the modified Stober process, ammonia is often preferred as the reaction medium, i.e., solvent, to provide the favorable alkalinity for the fabrication of MSNs. Further improvements have been made in replacing ammonia, referred to as pseudomorphic synthesis, widely appropriate for the transformation processes. This approach utilizes sodium hydroxide to establish the alkaline conditions for rapid condensation of silica over the surfactant micelles. In addition, triethanolamine was applied to provide alkaline conditions in the reaction medium as an alternative to sodium hydroxide [66]. The major advantage of triethanolamine is the generation of small-sized particles of ~ 20 nm, avoiding aggregation due to rapid hydrolysis.

To this end, the reaction-based factors include pH value, temperature, and stirring speed. Among these factors, the pH value plays a crucial role in silica's charge affecting the hydrolysis and subsequent co-condensation rates [32]. The charge of silica species differs depending on the pH value in terms of the isoelectric point, where the reaction medium attains a negative charge at a pH over the IEP value and vice versa [23]. Accordingly, the alkaline pH medium facilitates the highly negatively-charged silica, improving the assembly and condensation over the positively-charged species (cationic surfactants) through interactions between the contrary-charged species. Similar to the charge density, the silica condensation rate differs in displaying the mixed behaviors with changes in the pH value, i.e., increased rate of condensation to pH-7.5 and then decreases due to instability of silicates. In addition to the formation, the overall changes in the final shape of the particles are evident, possibly happened by using the different mixtures of the cationic surfactant species. Accordingly, it is highly convenient to fabricate MSNs with arbitrary sizes (several tens to hundreds) and shapes (spherical to irregular) by regulating the formulation and reaction conditions. In some instances, surface functionalization of MSNs utilizing diverse organosilanes may substantially control their morphology and help anchor for gatekeeping and immobilization of various guest species [67]. Accordingly, mesoporous architectures with uniform morphological attributes and altered physicochemical characteristics have been fabricated by systematically adjusting the conditions, such as surfactant templates pH and silica source [25, 40]. In this vein, several efforts have been dedicated by numerous research groups, including but not limited to Grun, Lin, Cai, Shi, as well as Mou et al. to fabricating MSNs with uniform particle sizes and ordered pore sizes, along with satisfied biocompatibility attributes [68–73]. Therefore, it should be noted that the appropriate particle size and surface functionalization are often taken into account for establishing the interactions with the biological membranes and improving the delivery of encapsulated guest species [43, 74, 75].

Despite the success in utilizing cationic surfactants as structure-directing agents, the fabrication process of conventional MSNs requires complex removal and subsequent immobilization procedures. To overcome these aspects, drugs can be co-loaded with the surfactants as chemosensitizers. The performance efficacy of the retained structure-directing micelles and their complexes is explored by monitoring the compatibility issues surfactants towards cancer therapy [76]. Similarly, the newly designed synthetic templates, i.e., drug-structure-directing complexes, have been employed to fabricate MSNs with exceptional functional and structural advantages [77–79]. Compared to traditional surfactants, these drug-complex templates exhibit higher stability and robustness, resulting in the MSNs with high surface area and pore volume. In this vein, Morales et al. designed several drug-based structure-directing templates to fabricate MSNs using various long-chain organic templates, such as fatty acids (decanoyl, palmitoyl, lauroyl, and oleoyl, stearoyl chloride) [78, 80]. The drug-complex templates-based MSN composites provided excellent pharmacological and nutraceutical efficacies, avoiding additional surfactant removal steps and functionalization and drug immobilization process [78]. Interestingly, the encapsulated drugs offered the sustained release of the encapsulated drugs both intracellularly and extracellularly for over months. In another case, the oil-in-water (O/W) emulsion-based synthesis of hollow-shell MSNs using the _L_-tryptophan with palmitoyl chloride as the drug-structure directing agent resulted in the peapod-like morphology [81]. Interestingly, the lamellar pillars were grown inside the hollow shells of MSNs. Further, the authors demonstrated the fabrication of MSNs to deliver the lipidic derivatives of cilastatin, a kidney protector [79]. Similarly, Stewart et al. explored the encapsulation of surfactant-like drugs (for instance, octenidine dihydrochloride) to fabricate the drugs structure-directing agents’ concept for antimicrobial therapy [77]. Despite the success, these complex templates resulted in large-sized MSNs of 100–700 nm with multi-dispersion and non-uniform distribution. Moreover, specific chemical linkages are often employed to fabricate the drug complex and the long-chain template molecule. However, the selection of template and drug, as well as suitable linkage reaction play crucial roles, determining their release and performance efficacies. Since reported recently, strict optimization of processing parameters is still required to explore their morphological and physicochemical parameters and suitability to various drugs.

## Engineered MSN prototypes

Indeed, MSNs are known for highly advantageous physicochemical characteristics and controllable morphological features. However, these stable siliceous constructs are merely reinforced as carriers for encapsulating and transporting diverse therapeutic guests (drugs/contrast agents) [29, 42]. Despite the success, several shortcomings include poor drug encapsulation and delivery capacities, degradability and compatibility issues, as well as reduced cellular internalization efficiency, limiting their applicability in medicine. Depending on the affinities between MSNs and guest molecules, these siliceous frameworks often result in poor encapsulation due to weak interactions between them, leading to the quick release of therapeutic cargo while loading with an exchange of surrounding ions [26, 82]. In general, the conventional hydrothermal approach often results in the robust siloxane (–Si–O–Si–) framework, which is highly challenging to be degraded in the physiological environment [83]. Although more stable than other similar generation materials, such as polymers and liposomes, to a considerable extent, the surface siloxane species could be degraded by slow hydrolysis, depending on the condensation rate during synthesis, specific surface area, porosity, and particle size, as well as the presence of immobilized groups [43, 83, 84]. In some instances, the presence of metal ions in the dissolution medium and polyethylene glycol coating (PEGylation) may facilitate partial degradation of MSNs [9, 85, 86]. Nonetheless, monitoring the uncontrolled degradation behavior of siliceous frameworks is highly challenging [76]. To this end, MSNs are considered biologically compatible due to two major reasons of the presence of surface hydroxyl groups and resultant silicic acid species from degradation. However, several contrary reports have enunciated that the MSNs would result in toxic signs in various cell lines in-vitro due to delayed degradation, exhibiting the accumulation-induced toxicity risks [87]. Moreover, it must present the improved cellular uptake efficacy to exhibit desired efficacy, irrespective of the cell surface. To this end, the negatively-charged MSNs are often limited with the cellular internalization efficacy as cell membranes are similarly charged, which, however, could be internalized through receptor-mediated endocytosis due to small size.

**Fig. 3:**
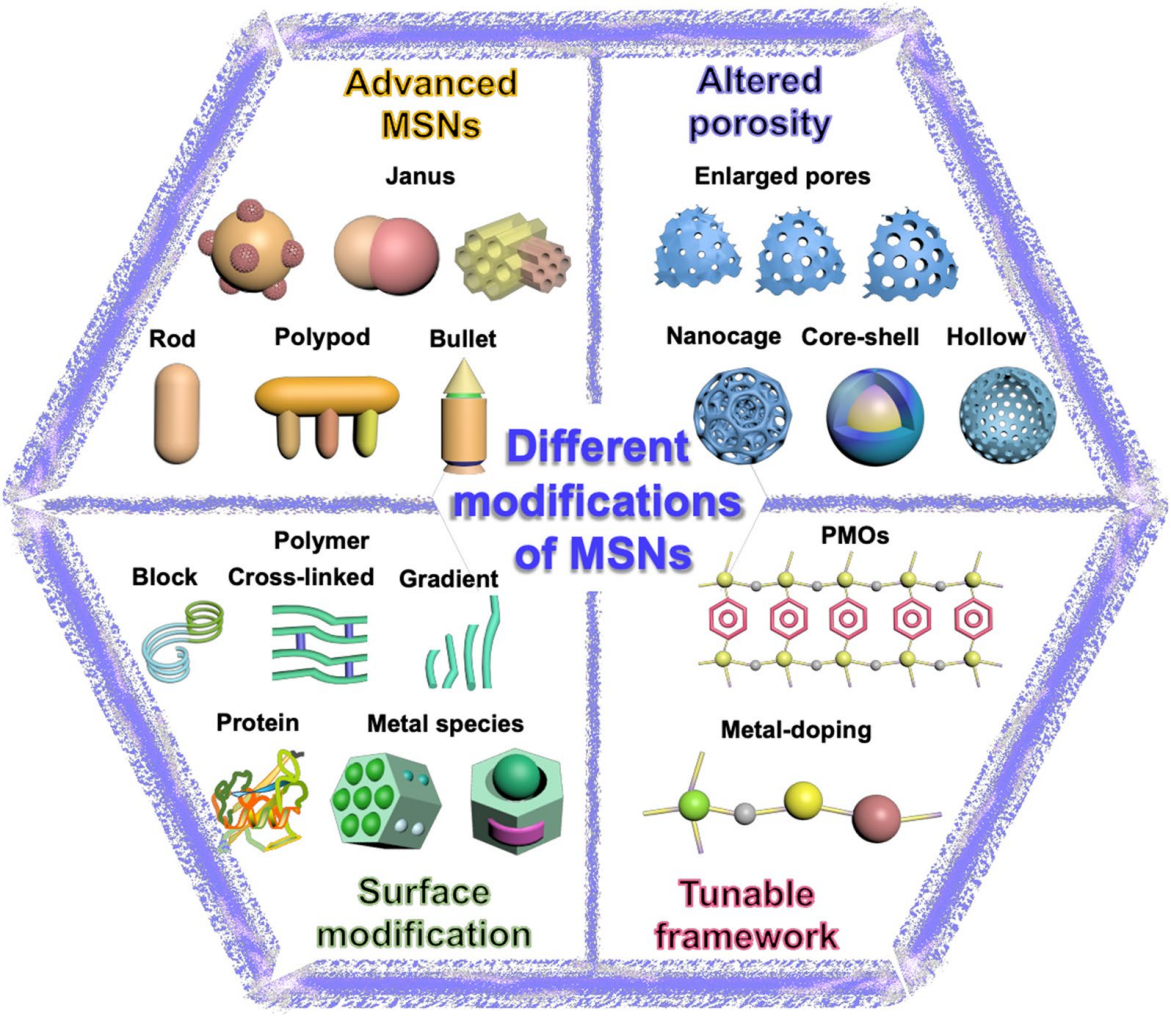
Illustration representing various categories of advanced prototypes of MSNs, including modified surfaces with various compositions, altered porosities, engineered siliceous frameworks, and specialized cutting-edge architectural designs (Janus & multipodal, bullet type, flower-shaped, and deformable architectures)

MSNs have been modified to fabricate advanced prototypes due to these critical limitations over the past two decades. In general, these advanced prototypes have been so far confined to different crucial aspects: (1) modifying the hydrophilic MSN surface by coating with biocompatible polymers/peptides/biological membranes [88–90]; (2) engineering the siliceous frameworks to improve their degradation with enriched properties [41, 91, 92]; (3) tuning the mesopore ordering toward fabricating hollow and cage-like structures [62, 93]; and (4) modifying the overall structure forming the complex architectural forms, such as Janus-type and flower-like architectures (Fig. 3) [43]. Notably, these precise modifications result in considerable changes not only in the resultant morphology (altered particle sizes and shapes) but also specific physicochemical characteristics, such as colloidal stability and surface characteristics, facilitating their improved applicability in various fields of biomedicine [6, 26, 33, 40, 42, 94–96]. Although the discussed series of modifications were described explicitly in our previous article, [43] herein, we briefly emphasize these advancements to provide insights, highlighting their pros and cons towards substantially enriching their applicability in biomedical applications.

### Modified MSN surface

Due to the extensive surface hydroxyl moieties, the hydrophilic surface of MSNs can be employed to conveniently modify through chemical functionalization and immobilize various functionalities on both the interior and exterior surfaces. The convenient immobilization of multiple functionalities on the versatile MSNs hydrophilic surface is often facilitated by either electrostatic interactions or covalent conjugation through post-grafting of organosilanes [6, 97–99]. Comparatively, the post grafting approach is safer and more efficient, requiring an additional step to immobilize the organosilane over the facile electrostatic interactions [37, 38, 100]. To this end, the electrostatic interactions depend on the attractions between the surface silanol groups of MSNs and cationic polymers (for instance, polyethylenimine, PEI) [101]. The surface engineering of MSNs through applying innovative chemistries, in terms of coating them with the supramolecular systems, offer numerous advantages: gate-keeping of the enclosed guest species improves their biological half-life (for instance, genes and proteins); enriching the biodegradability of the siliceous frameworks with susceptible coating materials; and ability to immobilize ligands (TAT peptides and nuclear localization signal, NLS) [102–104] for choking specific physiological barriers towards enriched, safe, and targeted delivery [73, 89, 105–109]. In this vein, several components can be employed to modify the surface of MSNs for exploring safe delivery. To explore these aspects, the modification of MSN surface with different materials is broadly divided into two different types of nanocomposites, i.e., organic (polymers, liposomes, biomembranes, and protein)-inorganic (mSiO_2_) hybrids and inorganic (metal/metal oxide shield)-inorganic (mSiO_2_) hybrids.

#### Organic coating

Among various organic modifiers to fabricating organic–inorganic hybrids, polymer coating plays a crucial role in modifying the MSN surface, which acts as one of the efficient controlled delivery vehicles in improving the performance and fate of various guest molecules through prolonging the therapeutic effects due to the structural diversities and different chemical functionalities [24]. Various classic polymers used in coating over MSNs include chitosan, alginate, polyethylene glycol (PEG), poly(2-vinyl pyridine), pyridine disulfide hydrochloride (PDS) Pluronic P123, and poly(2-(methacryloyloxy)ethyl ferrocenecarboxylate) (PFcMA) [85, 109, 110]. Some of these polymers provide additional benefits of offering sensitivity to various biological (pH and glutathione, GSH) and external (light, ultrasound, and temperature) stimuli, triggering the precise release of therapeutic guests from MSNs in the desired microenvironments and avoiding adverse events [17, 88, 111, 112]. Several examples of stimuli-responsive polymers coated over MSNs include poly(acrylic acid) (PAA), polyvinylferrocene (PVFc), chitosan, poly(*N*-isopropyl acrylamide) (pNIPAAm), poly(2-(diethylamino)-ethyl methacrylate) (PDEAEMA), and poly(2-phenyl-1,3-dioxan-5-yl methacrylate) [106, 109, 113–116]. Nonetheless, the chemical functionalities play crucial roles in the post-grafting of polymers over MSNs. Despite the success in coating with various polymers, in some instances, it may limit the targeted delivery of therapeutic guests [64], thus requiring to immobilize a targeting ligand to enrich the targeted delivery. For instance, Arg-Gly-Asp (RGD) ligand was immobilized over PDS and PEG-coated MSNs for receptor-mediated internalization [112]. In addition, the targeting ligands immobilized over MSNs would substantially improve the delivery efficiency intracellularly by overcoming the macrophage uptake and combating the multi-drug resistance in cancer and bacteria [109, 117]. In addition, the bilayered liposome is coated over the structure to offer additional compatibility features and improved delivery efficacy [19, 117]. However, it is often preferred to add positively charged constituents to form a protective coat due to a similar charge. Similarly, biological membrane-based supramolecular architectures are cloaked over the MSNs surface, such as extracted cell membranes of cancer, RBC, and macrophages, among others [90, 118–121]. Comparatively, these biomembranes directly camouflaged as coats over MSNs are more advantageous over polymers and liposomes, in terms of improving hemocompatibility, offering long-term circulation, providing macrophage escape, and delaying renal clearance. These properties of camouflaged-MSNs enable targeted tumor precision therapy and improved internalization efficacy through substantial entanglement with the cell membranes due to similar composition [120–122]. Nonetheless, the progress remained at the infant stage, requiring mechanistic exploration in terms of interactions between the bio-nano interfaces within the composite, as well as cloaked composites and cell membranes.

In addition to complete biological membranes, a unique strategy of coating biological constituents (for instance, proteins) has also been demonstrated to establish specific bionanomaterials for different biomedical applications [63, 123]. In general, the successful encapsulation of proteins in the interiors of frameworks can safeguard from denaturation and enable to deliver towards improving their efficacy [63, 123–125]. Owing to the abundant hydrophilic surface, MSNs can be coated with the proteins towards improving the interactions with the cellular membranes, facilitating the improved internalization and gating of encapsulated guest species towards subsequent enriched delivery in-vivo [126]. The classic examples of various proteins include albumin, hemoglobin, streptavidin, Concanavalin A (Con A), biotin-avidin complex, and biotinylated transferrin [100, 127]. In addition, the stimuli-responsiveness (glucose, pH, thermos, and light) of the gated proteins over MSNs has subsequently improved the delivery efficacy of therapeutic guests precisely [100, 127]. In addition to precise delivery, some proteins covered over MSNs through covalent interactions may result in stable corona, optimum for biosensing [128]. For instance, covalently-linked Hb and glucose oxidase (GOD) were coated over MSNs as multiple layers, which would be applied as biocompatible biomarkers due to the autofluorescence property and catalytic devices [128]. Despite the convenience of immobilizing such macromolecules, these surface-coated proteins might be prone to denaturation when administered in-vivo*.*

#### Inorganic shielding

Another type of surface-modified nanohybrids includes inorganic-inorganic composites. Several metal nanoparticles (MNPs) can be arranged as shields over the MSN surface, for instance, iron (Fe), cobalt (Co), gold (Au), copper (Cu), silver (Ag). Noticeably, these arranged MNP-based shielding offers excellent physicochemical and optoelectronic attributes [129]. In addition, species like iron-based constructs allow the composites to explore the paramagnetic behavior in applying them not only for magnetic field-assisted targeted drug delivery, leaving the surrounding tissues safe but also for magnetic resonance imaging (MRI), enabling ease of diagnosis [130–132]. Moreover, such paramagnetic behavior facilitates the ease of separation towards the reusability of the constructs [129, 133–135]. In this vein, diverse MNPs (iron oxide, cadmium sulfide, CdS, AuNPs, and AgNPs) have been utilized towards modifying the surface of MSNs, via tethering molecular or supramolecular gating groups for drug delivery (drugs, biocides, genes, proteins, and dyes) [33, 68, 88, 97, 136–145]. Despite the success in exploring the stimuli responsiveness-assisted delivery of encapsulated guests [140], the compatibility issues of the surface-modified MNPs, along with the silica density, would result in adverse events, for instance, inflammation, which yet remained to be addressed [146].

### Altered porosity

Indeed, MSNs were named due to their porosity range of 2 to 50 nm based on the International Union of Pure and Applied Chemistry (IUPAC) nomenclature [9, 30]. These porous architectures facilitate the harboring of various guest molecules and their delivery without any signs of destabilization in their highly inert siliceous frameworks [2]. Although the MSNs possess highly ordered porous architectures, it is highly challenging to encapsulate a high amount of guests and their release, as well as large-sized and dense therapeutic guests, towards improved therapeutic efficacy [147, 148]. Owing to these facts, the porosity of ordered MSNs can be reorganized towards fabricating MSNs with enlarged pores and hollow-type MSNs (HMSNs) for accommodating large-sized or highly dense biomacromolecules.

#### Enlarged pore size

To encapsulate bulk molecules, the mesopore size is often tuned by using various auxiliary agents, i.e., swelling agents or enhancers (for instance, TMB, *n*-octane, *n*-decane, *N*, *N*′ dimethylalkylamines) [36, 63, 69, 149–151]. Despite the increased mesopore sizes, the major limitation lies in the fact that it is highly challenging to preserve these ordered mesostructures [152]. Further, several polymers/surfactants have been applied in generating different mesoporous features, such as disordered or ordered, unimodal or bimodal, and diameters of 5 to 30 nm [23, 153, 154]. These classic additives include PEO-PPO-PEO, polystyrene-poly(ethylene oxide) [155], and polystyrene-polybutadiene-polystyrene [156–158] as well as Brij surfactants (Brij 56 and 76) [159, 160]. Although the generation of large-pore sizes is highly conducive by adding auxiliary pore expanders, it is highly challenging to control the collapse of crystalline mesostructures' geometrical phase change after addition [149]. Notably, the type of composition of surfactants plays a crucial role in eventual mesoscopic characteristics. Interestingly, the large-pore-sized MSNs were fabricated using PS-*b*-PAA copolymer at an altered CTAB concentration, resulting in the cubic *(Fd3m)*, hexagonal, and lamellar porous architectures [36]. In addition to pore size, the overall pore volume can be regulated by altering the other reaction conditions, such as additional treatment with *N*, *N*′ dimethyldecylamine, and increased temperature, among others [153, 161, 162].

#### HMSNs

In this vein, pore engineering has been employed to further address the shortcomings of conventional MSNs in accordance with the storage and transportation of bulk proteins [148, 163, 164]. These HMSNs can be prepared using soft- and hard-templating strategies, in which the former utilize amphiphilic surfactants as single vesicles [165, 166], micelles [167], and microemulsion droplets [168], as well as the latter, employ dissolvable or combustible interiors (polymer beads based on PS, polymethylmethacrylate, PMMA, polyvinyl pyrrolidone, PVP, and pNIPAAm, as well as metal/metal oxides/semiconductor nanoparticles of CdS, Ag, Au, zinc sulfide, silica, hydroxyapatite, and calcium phosphate) as the hard templates [11, 148, 164, 169]. Among various facile soft-templating approaches, the micelle-based strategy results in tiny HMSNs, which are not appropriate for delivering large amounts of therapeutic cargo. To a considerable extent, hydrophobic expansion agents, for instance, poly(styrene-*b*-2-vinylpyridine-*b-*ethylene oxide) (PS-PVP-PEO), can be added to improve the inner hollow void spaces [170]. Notably, the final size of HMSNs can be improved by packing the micelles at a high packing parameter, leading to large-sized globular hollow architectures of 100 nm and core–shell composites [23, 171]. To this end, a microemulsion system can be applied as the complex soft templating approach employing hybrid phases (O/W) to fabricate HMSNs [168]. Applying the expansion agents in this complex templating approach has significantly improved mesopore sizes, such as kippah-shaped HMSNs [172, 173].

To this end, the hard-templating method utilizing various polymeric beads and MNPs is the most effective approach to generating discrete, uniform, and mono-dispersed HMSNs from several tens of nanometers to over a micron in diameter. Utilizing polymer beads offers more advantages as they are cheaper than the MNPs and can be extracted through facile acid-dissolution, solvent extraction, and calcination (400 °C) strategies [11, 148]. On the other hand, the MNPs are more expensive and require hazardous or corrosive solvents to extract from the core [148]. Notably, this hard templating approach can be applied to accommodate metal/metal oxide cores, resulting in customized core–shell hybrid nanoarchitectures in the optimal reaction conditions. The functionalities and advantages of the individual components can be applied for synergistic theranostics [6, 174–180]. The removal of the core MNPs through various extraction approaches may result in the formation of HMSNs, which is notably expensive and time-consuming [180, 181]. In some instances, the generation of core–shell architectures has been improved by coating surfactants or polymers over the MNPs before silica casting, resulting in the uniform distribution of silica shell coating over the MNPs [15, 174–176]. Moreover, the major limitation of the hard-templating approach is the controlled synthesis of HMSNs in small sizes with MNPs in interiors [182]. In addition, several other approaches have been applied to fabricate yolk-shell architectures, such as selective etching, Ostwald ripening, bottom-up, template-free, ship-in-bottle, Kirkendall effect-based strategies [174–177, 180, 183–187]. Nonetheless, the generation of uniform-sized constructs is conducive by applying a binary surfactant mixture of anionic and zwitterionic surfactants, leading to vesicular structures encapsulated with highly stable MNPs [183, 188, 189].

### Re-engineered siliceous frameworks

Due to the intrinsic stability of highly dense siliceous frameworks, MSNs often suffer from major disadvantages of deprived biological compatibility and poor degradability, resulting in the reduced elimination-induced toxicity risks invivo [9, 41, 42, 83]. Notably, the degradability of MSN frameworks in the highly complex biological environment is one of the critical attributes related to their compatibility. Moreover, the encapsulation of diverse therapeutic guests in the interiors of MSNs is often limited due to the poor interactions between the silica host and the guest species [110, 190]. Despite various modifications of modifying the surface with biocompatible polymers and altered porosity, several concerns of compatibility and degradability in medicine remain unaddressed, leading to their inadequate clinical translation. To this end, the third kind of modification, i.e., the precise engineering of siliceous networks, can address these intrinsic shortcomings through impregnating various species with contrary charge and modifying the patterns of siloxane species arrangement, for instance, organic (periodic mesoporous organosilicas, PMOs) [83, 147, 190] or inorganic (transition metals, divalent and trivalent) moieties [82, 92, 96, 110]. The supramolecular arrangement of the condensed silica species with the other species has generated massive scope in fabricating advanced prototypes of MSNs, which subsequently improve the degradability of MSNs and facilitate encapsulation efficacy as well as the stimuli-responsive release of guest species [179, 190, 191]. These successive modifications have unlocked new-fangled prospects for these emerging materials in diverse biomedical applications [179, 192].

#### PMOs

PMOs are often referred to as an innovative class of hybridized mesoporous covalently-bonded siliceous frameworks containing organic and inorganic components distributed homogeneously to offer new expanding possibilities and reconnoitering pioneering applications [45, 190, 193]. Similar to traditional MSNs, these PMO matrices are generally fabricated by sol–gel process, involving the cocondensation of organic groups-bridged silanes instead of sole silica precursor (TEOS) [147, 194]. Parallel efforts from Stein, Inagaki, and Ozin generated diverse PMOs initially using the low Mol. Wt. organo-silanes (methane, benzene, ethylene, and ethane-bridged groups), which substantially improved the siliceous frameworks' physicochemical attributes and mesopore ordering [45, 194, 195]. Although incorporating organic moieties improves the degradability features, the arrangement of pore walls is of significant concern. Initially, Mizoshita et al. presented that the pore walls were arranged as regularly packed columnar assemblies due to the hydrogen bonds facilitating molecular-scale ordering of pore walls [196]. Further research has explored that the change in the bridging organic moiety would certainly facilitate improved scope in their application in medicine due to improved degradability and compatibility attributes and reduced sizes for effective drug delivery [83]. Further efforts have been dedicated to altering the organo-siloxane, for instance, ethylene [197], biphenyl [198], divinylbenzene [199], thiophene [200], bis-imidazolium [86], and 2,20-bipyridine [201], resulting in the assorted varieties of PMOs (20–500 nm) and diverse shapes (wormlike to spherical) [190, 202]. Interestingly, several studies have reported that the utilization of mixed organosilane precursors would improve the surface area significantly [83, 203]. These diverse varieties of PMOs have been applied in various fields of adsorption, catalysis, applied as synthetic templates, enzyme immobilization, protein separation, and drug delivery [83, 179, 192, 204].

Due to the lower silica density than pristine MSNs, PMOs show improved degradation and compatibility attributes, facilitating their augmented applicability in medicine, for instance, disulfide-bridged composites [66, 83, 190, 205, 206]. Further, the applicability of PMOs could be improved through impregnating larger-sized organic functionalities with stimuli-responsive linkers in the siliceous frameworks towards fabricating hybrid composites [83]. Nonetheless, the colloidal stability of the fabricated hybrid systems must be of prior importance while fabricating such systems. Moreover, strict optimization is required to regulate the morphological attributes and encapsulation parameters for effective loading and improved applicability. Further, the mechanistic elucidations related to encapsulation and favorable release of therapeutic guests precisely for improved therapeutics. In addition to improving the fabrication and performance efficacies, the viewpoint and detailed mechanistic insights of safety and in-vivo fate attributes must be considered, as these are the necessary factors for the clinical translation owing to the highly complex biological microenvironment [207].

#### Metal impregnation

To a considerable extent, the surface engineering of MSNs with contrary-charged polymers and camouflaging biomembranes could modify their in-vivo fate through overcoming the extracellular repulsions. However, the intracellular fate of MSNs remains a significant challenge during the drug delivery application in terms of reduced degradability and performance efficacy. Although the MSNs offer exceptional mechanical and thermal stabilities, the amorphous character of silica hinders their applicability [208]. To address these intrinsic limitations, the re-engineering of MSNs would considerably facilitate the altered charge density of the MSN framework. Similar to PMOs, the generation of altered MSN frameworks by incorporating positively charged metal species, such as transition metals (Co, Fe, Cu, and Ni) with excellent electronic architecture, is appropriate for varying the overall charge density [82, 95, 96, 129, 209, 210]. Notably, several attributes must be considered while metal incorporation, such as the reactant concentrations of silica-to-metal ratio and arrangement of pores. The eventual mesoporous frameworks get distorted or disordered at higher concentrations, resulting in irregular shapes and separation as metal oxides [208]. The impregnated meal species in the siliceous pool would considerably affect their performance at a lower concentration due to deprived loading efficiency. After critical optimization, our group had observed that the concentration of silica-to-metal ratio of 30:1 in Cu species could be optimum for biomedical applications considering the morphological attributes and subsequently augmented encapsulation and delivery efficacies [82, 96, 110, 129]. However, it should be noted that the concentration may vary with the change in the metal species [92]. Further, the arrangement of mesopores must be considered while metal impregnation, such that it should result in deep and large volume pores to improve the loading efficiency of the therapeutic guest molecules significantly [82, 129, 209].

Concerning the biomedical applications, these pristine MSNs often suffer from the deprived encapsulation efficacy of therapeutic guests due to poor interactions between the inert silica host and therapeutic guests. The incorporation of therapeutic guests in conventional MSNs is often favorable through physical adsorption, resulting in their rapid withdrawal from the mesopores in exchange with the surrounding ions during encapsulation [26, 116]. In this vein, our group has presented several studies to impregnate metals in the MSN frameworks, i.e., divalent, Cu(II), and trivalent, Fe(III). These impregnated metal species substantially improved the loading efficacy of therapeutics through establishing stable coordination interactions that are pH-responsive, specifically in the acidic environment of cancerous tissue and infectious bacterial site [82, 96]. In addition to building the high-loaded metal-impregnated carriers, these composites present the improved cellular internalization and favored delivery due to the altered surface charge density that facilitates the enhancement of the interactions of the carriers with the negatively charged biomembranes over the inert silica carrier [96]. Nonetheless, it should be noted that the coordination linkage is often established with the amine functional groups, limiting their universal applicability with all kinds of therapeutic drugs.

In addition to encapsulation and delivery, the impregnated metal species in the siliceous frameworks of MSNs would participate in the performance of these innovative carriers. The versatile metal species can act as nanomachinery elements and transform the naturally available molecules (hydrogen peroxide, H_2_O_2_) to deadly cytotoxic radicals (i.e., reactive oxygen species, ROS) at the diseased site. This specific chemical transformation is referred to as Fenton-like reactions, which happen to be favorable due to the specific electronic architectures of transition elements (Cu and Fe species), resulting in tumor ablation [96]. Notably, this process is often higher in the diseased sites due to higher H_2_O_2_ levels as a part of antioxidant defenses than normal cells [129]. Further, our group had explored the incorporation of such metal species to explore the synergistic effects of chemodynamic therapy [92]. Interestingly, the similarly charged transition metal species changed the shape of MSNs due to repulsions between the impregnated transition metals, leading to sphero-ellipsoid. Such altered shapes with improved intrinsic functionalities of MSNs would undoubtedly open new burgeoning opportunities in utilizing them towards innovative applications.

### Specialized architectural designs

Apart from the conventional modifications of MSNs in terms of modifying surfaces, altering porosity, and engineering intrinsic siliceous frameworks, several other advancements in the past decade have evidenced the generation of redesigned architectures through reshuffling their molecular arrangement patterns [124, 211, 212]. Despite the rigid siliceous mesoporous frameworks of MSNs, re-engineering siliceous frameworks into hetero-nanostructures by changing their shape and geometry leads to such specialized architectural designs of Janus-type and multi-podal structures as well as dynamically-regulated deformable solids and advanced nature-simulating (flower-shaped) and biologically-inspiring (tadpole-like) architectures.[25, 26, 213–215] With the application necessities as principal concerns, these specialized designs offer exceptional performance in terms of stimuli-responsive release, as well as degradation, and compatibility in-vivo [210].

#### Janus-type architectures

The innovative Janus-type composites, often referred to as irregular-shaped anisotropic structures, offer augmented physicochemical and morphological attributes owing to their distinguished surface properties and structural attributes [16, 121, 216–218]. Notably, these architectures not only overcome the shortcomings of conventional Janus structures made of silica and polymers but also address the limitations of pristine MSNs, such as poor encapsulation efficacy and augmented physicochemical and morphological attributes [211, 219, 220]. In most instances, additional constituents, such as diverse varieties of MNPs, are often applied to prepare the versatile Janus-type architectures, which would instead provide improved stability due to altered charge density and superfluous corresponding optical, electrical, and magnetic characteristics [16, 212, 221–223]. In one case, UCNPs-enclosed MSN shells with dual-independent mesophases were fabricated to encapsulate two different guests for therapeutic co-delivery [211]. In another case, electron beam evaporation-assisted metal coating resulted in irregularly deposited islands on MSN surface, towards powering them as self-thermophoresis-based cargo delivery [224]. Similarly, vacuum sputtering led to the fabrication of metal coating over MSNs as hemispherical cap-like Janus structures [212]. These irregularly distributed metal coats over the MSN surface could be applicable as therapeutic carriers or nanocatalysts [218, 224–226]. Despite the success in exploring the versatile functionalities, further studies in terms of performance optimization are needed to be explored, as safe transportation through self-thermophoresis is always a challenging issue, requiring additional protection in terms of gatekeepers or capping. Moreover, the multi-step fabrication of Janus-type architectures is indeed another challenge to be addressed as the multiple synthetic steps might result in mechanical abrasion, leading to poor morphological features and deprived loading efficacy, as well as therapeutic performances. To overcome this shortcoming, our group has demonstrated the utilization of multiple transition elements (Cu and Fe) to alter MSN networks using the modified Stober process, resulting in the Janus-type architectures. The metal species with similar charges were rearranged due to repulsions during the condensation, resulting in the sphero-ellipsoid nanoarchitectures [92]. Interestingly, the reduced inert silica content and increased metal-based coordination linkages between the silica and metal species had improved the drug loading and releasing efficiencies precisely and resulted in the degradation of the nanocontainers.

#### Multipodal and nature-mimicking designs

In addition to Janus-type architectures that are generally referred to as a single pod-containing structure, MSNs with multiple pods are another type of highly complex morphological architectures [227, 228]. Similar to Janus-type architectures, the multi-pod-like structures offer selective encapsulation of multiple therapeutic guests in different pods towards their precise delivery. Initially, Wiesner et al. generated silica-based two-dimensional (2D *P*6*mm*)-hexagonal pods on their cubic cores (*Pm3n*) porosity using the epitaxial growth [229]. Further, Croissant et al. fabricated hybrid, crystal-like multipodal architectures of PMOs, in which the pods of ethylene-bridge PMOs were condensed over the core of benzene-bridged PMO [230]. Although these interesting designs presented exceptional performances, several aspects of reproducibility at the optimal synthetic conditions and exploring the performance attributes at various in-vitro and in-vivo levels yet remained to be addressed comprehensively.

Apart from the diverse varieties of synthetic architectures, several nature-inspired designs have been fabricated, specifically flower-shaped and streamlined tadpole-like MSNs. In a case, MSNs with the core-cone structure were prepared by packing silica cones over the MSN core in water-chlorobenzene, resulting in the MSN nanoflowers [124]. The cones arranged over the core with enlarged openings provided high pore volume, which could be convenient for delivering large-sized/high Mol. Wt. therapeutic guests (for instance, proteins). Despite the success in fabricating flower-like arrangements, the stability of arranged cones is challenging, resulting in detached cones. In addition, several radially porous, flower-mimicking designs, referred to as dendritic MSNs, have been fabricated to encapsulate a large amount of cargo along with complex molecules, such as proteins, nucleic acids, and vaccines [231, 232]. Interestingly, Lee et al. generated the iron oxide-based clusters as core materials decorated with the large porous siliceous shell and PEI for vaccine delivery [233]. The addition of ethyl acetate in the ammonium hydroxide solution prior to TEOS addition resulted in the extra-large-porous shell by reducing the polarity of the sol–gel process for enhanced cancer vaccine delivery (further details of vaccine delivery are provided in the Cancer Immune Therapy) [234].

**Fig. 4:**
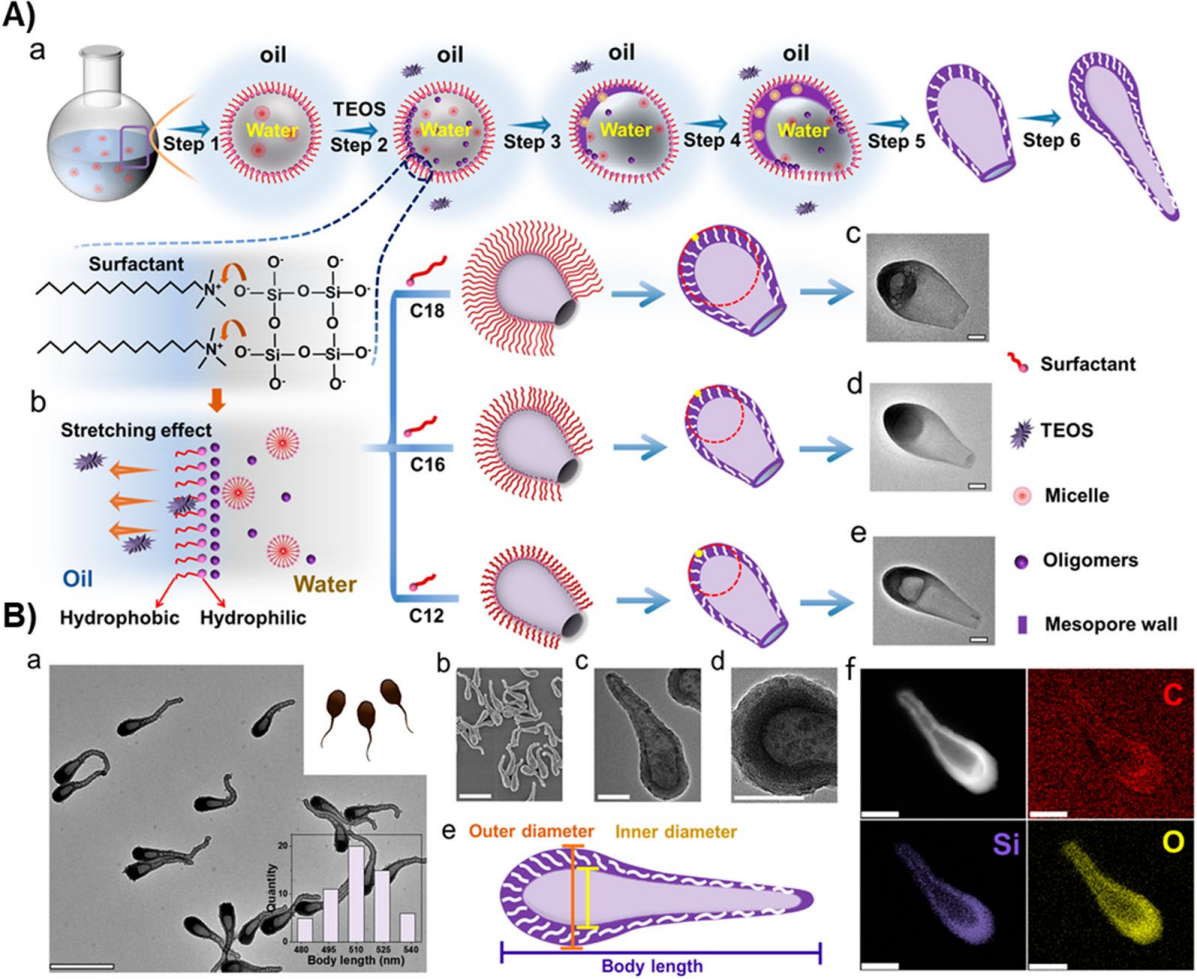
**A** The illustration of interfacial dynamic migration mechanism. **a** The formation process of the streamlined mesoporous silica nanoparticles. **b** Schematic illustration for the curvature regulation of the streamlined mesoporous nanoparticles by using surfactants with different chain lengths. **c**–**e** TEM images of the streamlined silica nanoparticles with different curvatures. All scale bars are 50 nm. **B** Streamlined hollow mesoporous silica nanotadpoles. **a**, **c**, **d** TEM images with different magnifications, **b** SEM image, and **e** structural model of the mesoporous silica nanotadpoles prepared by the interfacial dynamic migration strategy. **f** Element mapping of the mesoporous silica nanotadpoles. Scale bars represent 500 nm in panels **a** and **b**, 100 nm in panels **c**, **d**, and **h**. Insets in panel **a**: digital photo of the tadpoles (upper right corner) and the corresponding distribution histograms of the body length of the mesoporous silica nanotadpoles (lower right corner). Reproduced with permission from Ref [235]. Copyright 2021, American Chemical Society

In addition to several non-flexible designs, recent research has evidenced the fabrication of some innovative nature-mimicking, highly flexible, streamlined architectures, which look like tadpoles, fishes, and sperm-like structures. Ma et al. designed the asymmetric streamlined MSN architectures with tunable surface curvatures and larger inner cavities [235]. These structures with ellipsoidal heads and a contracted tail offered a low fluid resistance coefficient for enhanced locomotivity. Employing the dynamic migration strategy resulted in adjusting the tail length to over 500 nm by controlling the reaction time and manipulating the inner cavity from open to closed (Fig. 4). Initially, the W/O emulsion containing CTAB as a stabilizer with oil phase as pentanol, in which the streamlined tadpole-like structures were obtained by dynamic migration of the interfaces between pentanol and water and the stretching effects of the phases resulted in the curvature radius. Moreover, regulating alkyl chain lengths at the dynamic surface enabled the tuning of the head curvatures. The authors demonstrated several physicochemical attributes and functional coefficients that explore the synthesis optimization and functional characteristics. Owing to the streamlined morphology and diffusional performances of these large porous containers, iron oxide was enclosed to explore the H_2_O_2_ driven motor-like movements, which would undoubtedly open the new burgeoning area towards biomedical applications.

#### Deformable solids

Indeed, MSNs are highly stable due to their rigid siliceous frameworks. The irreversible stiffness of intricate siliceous frameworks, in some instances, may limit their penetration efficacy owing to complex biological barriers [55]. Nonetheless, it is possible to engineer and conveniently make these siliceous frameworks deformable by impregnating relatively flexible cross-linked organic groups in MSNs [210]. These flexible groups allow changes in the shape of MSNs in the physiological environments, referred to as deformability due to biological stress, while crossing the biological barriers, enabling the augmented internalization more conveniently than traditional MSNs [144, 236]. Moreover, this deformable attribute offers improved bioavailability of various drugs in tumors along with long-term circulation. In a case, PMOs-based nanocapsules with highly deformable frameworks were fabricated using the preferential-etching process [210]. These capsules substantially improved the cellular uptake by altering their shape while crossing the biological barrier from sphere to oval, resulting in the augmented bioavailability by 26-fold over traditional MSNs. Although these materials look soft like liposomes and polymers, these deformable materials are mesostructured with certain exceptional physicochemical features, which could be reliable in treating and diagnosing various diseases [210].

## Biomedical applications

### Drug delivery

Indeed, the conventional therapeutic molecules alone or in combination with specific polymeric hosts often result in poor therapeutic performance due to limited bioavailability of drugs, enormous intrinsic toxicity, poor colloidal stability, and non-specific interactions with the biomolecules in-vivo. Since the magic bullet concept by Paul Ehrlich was proposed, the nanotechnology-based devices and their advancements have evidenced the fabrication of diverse varieties of controlled and targeted drug delivery nanoplatforms, as well as diagnostic and sensing systems towards versatile theranostic nanomedicine. These intelligent carriers could offer exceptional physicochemical and morphological attributes to improving the bioavailability and pharmacokinetic biobehaviour of drugs [220]. In addition, these versatile nanomedicines offer high encapsulation, avoiding premature leakage, stimuli (pH/thermos/light)-controlled delivery in a spatial-controlled manner towards achieving desired therapeutic action at the pathophysiological region of interest. In this regard, MSNs have been employed as carriers for delivering various therapeutic guest molecules due to their ordered pore fabrication and high surface area, providing the mesostructured architectures for hosting the drug cargo. In addition, this attribute of encapsulation ability facilitates the enhancement of solubility of hydrophobic drugs through adjusting the porous morphology [74, 82]. To further enhance the performance of these robust and highly stable carriers, several advancements have been made to significantly control the release of drugs from the mesoporous by surface functionalization due to abundant surface chemistry. In this vein, installing various molecular and metallic species in MSNs not only enriches their encapsulation but also offers controlled as well as targeted delivery efficacies for augmented therapeutic effects. Moreover, the beneficial properties of MSNs as innovative carriers can be harnessed by decorating with uniquely designed gate-keeper molecules over the mesopores, facilitating the controlled release of drugs. This section provides an emphasis on these advanced MSNs-based carriers with a set of notable examples of molecular linkages and metal species immobilized in the siliceous frameworks, responding to one or more stimuli for diverse theranostic applications.

#### Controlled delivery of chemotherapeutics

##### Molecular and supramolecular linkages

Over the decades, enormous progress has been evidenced by incorporating various selected promising molecular or supramolecular linkers in MSNs, which could be well controlled through different complicated mechanisms. These innovative switches include biological stimuli (pH/molecular) and externally-applied triggers (light/ultrasound/temperature/magnetic field), which effectively stimulate the delivery of various drugs/genes/therapeutic peptides (Table 1) [8, 237–242]. In cancer, these theranostic nanomedicines carrying the chemotherapeutics are targeted passively to tumors based on the enhanced permeation and retention (EPR) effect due to leaky vasculature, resulting in their accumulation specifically in tumors. Notably, these stimuli-responsive strategies are expected to afford time-, dose- and spatiotemporal-controlled release in the tumors both in-vitro and in-vivo systems [11, 243–245]. Collectively, these effective stimuli-responsive systems show significant impact in not only drug delivery with improved drug loading and release but also in protecting encapsulated drug cargo from premature release under physiological conditions reducing adverse effects and extravasation by RES uptake [246].

**Table 1:** Various advanced prototypes of MSNs conveying therapeutic cargo for various stimuli-responsive delivery

Type of Stimuli	Advanced composites	Advancement/modifications	Morphology	Cargo	Particle size	Targeted site/outcome	Refs
pH-responsive	V7-RUBY	Wormhole pore, Chitosan-coated, and V7 peptide-modified	Spherical	IR780 dye, paclitaxel, or carboplatin	< 40 nm	The tumor-specific-targeted release presented improved therapeutic effects against the orthotopic ovarian tumors	[258]
	LB-MSN-OVA	Lipid bilayer-coated over the surface	Rectangular	Ovalbumin	~ 200 nm	MSNs-encapsulated in microneedle arrays showed exceptional intradermal antigen delivery	[260]
	CS-PtNPs@Zn-MSNs	CS-coated over the MSNs	Spherical	DOX	~ 100 nm	pH-responsive CS degradation facilitated the convenient delivery of MSNs intracellularly, overcame the MDR, and offered PtNPs-assisted deep tumor penetration	[110]
	Triple-labeled MSNs	YQRLGC-peptide conjugated, PEI/PEG/THPMP	Spherical	FITC, OG, and RITC	~ 200 nm	These lysosome-targeted nanoprobes enriched the understanding of the fate of MSNs intracellularly	[257]
	FCA@mSiO_2_	Fe_3_O_4_ coated carbon/silver (FCA) as core and mesoporous silica as shell	Core–shell	Fe^2+^, artemisinin	~ 200 nm	Artemisinin-loaded FCA@mSiO_2_ presented the acid-specific release of Fe^2+^ ions to non-enzymatically convert artemisinin to toxic species for cancer ablation	[430]
	Cu-Fe-MSNs	Dual metal-impregnated constructs	Janus-type	DOX	~ 100 nm	Impregnating two similarly-charged metals facilitated shape changes and promoted the ROS-assisted CDT	[92]
	HMSNs-β-CD-AD-PEG	Multiple surface-modified pH-responsive linkers, benzoic imine and boronic acid ester	Spherical	DOX	~ 100 nm	These PEG-coated, CD-gated, hollow MSNs with cascade pH stimuli cleaving the benzoicimine bonds and boronic acid ester presented excellent intracellular delivery	[431]
	M-CHO-DOX@DOX-PEG	pH-sensitive dynamic benzoic–imine covalent bond as capping	Spherical	DOX	~ 160 nm	Dynamic PEGylation via benzoic–imine bond further endowed the drug-self-gated nanocarrier with tumor extracellular pH-triggered cell uptake and improved therapeutic efficiency in-vivo	[240]
	DOX-MSN-CF127	Polymeric micelle (F127-CHO)-gating	Spherical	Curcumin, DOX	~ 70 nm	Multifunctional stimuli-responsive opening of polymeric micelle cap improved the drug delivery and optical imaging	[432]
	MSN–R848	Heterobifunctional cross-linker maleimide-PEG-NHS modified and biotin-avidin capped	Core–shell	R848 and OVAp	~ 70 nm	The nanocomposites with pH-responsive acetal linker presented the release of R848 cargo and offered dendritic cells activation as well as enhanced cytotoxic T-cell responses	[256]
	MSN-PAA-PEG	Optimized degree of polymerization with escalated PAA unit number in PAA-PEG	Spherical	AZD6244 and PLX4032	< 100 nm	The pH-responsive on-demand controlled release from MSNs reversed the MEK-inhibitor-induced suppression of activated CD8 + T-cells and enhanced the secretion of INF-γ and IL-2	[255]
	MSN-Fe-AuNPs	AuNPs-Cys as gatekeepers, pH-dependent photothermal conversion	Core-satellite	DOX	~ 100 nm	Fe-induced AuNPs presented combined photothermal therapy, chemotherapy, and Fenton reaction-based tumor therapy	[433]
	MSN-WS_2_-HP	WS_2_QDs-HP, tLyP-1	Spherical cluster bomb	DOX	~ 50 nm	The pH-responsive size-changeable constructs presented the CendR pathway and NIR-light-triggered photothermal ablation of 4T1 tumors	[434]
	Lipid-PEG coated silicasomes	Lipid bilayer and PEG-coated constructs for co-administration of anti-PD-1 antibody	Silicasome	DACHPt	~ 140 nm	DACHPt silicasome by anti-PD-1 antibody presented excellent chemotherapy and ICD response in orthotopic Kras-derived pancreatic cancer	[262]
		PEG and lipid bilayer coat A linked downstream cascade	Core–shell silicasome	IRIN	< 100 nm	These composites with scale-up features presented improved therapeutic efficacy against robust treatment-resistant Kras-induced pancreatic cancer	[263]
	USMO@MSNs	Ultrasmall manganese oxide-capping over MSNs	Core–shell structures	DOX	~ 50 nm	The designed nanocomposites presented MRI-guided pH-switching theranostic performance for synchronous MRI diagnosis and chemotherapy	[435]
GSH-responsive	MSN-S–S-NAC-Trp	Disulfide bond and short peptide as capping agents	Spherical	DOX	~ 90 nm	A bolt-like blocking nanovalve presented GSH-responsive release for HeLa cell apoptosis	[291]
	DMSN-DP@CM	MCF-7 membrane coated	Core/shell	DNA fuel strands	243 nm	GSH-responsive DNA strands in DMSNs posed to Immune escape and homotypic-targeting	[364]
	MSNs-S–S-siRNA	Disulfide capping	Spherical	DOX and Bcl-2 siRNA	80 nm	Synergistic tumor growth inhibition in-vivo showed potential chemotherapy and gene therapy	[292]
	CDs@MSN-TPP@AuNPs	TPP and AuNPs coated over the MSN surface	Spherical	DOX	~ 40 nm	GSH-responsive etching of AuNPs provided effective cancer therapy and mitochondrial-targeted imaging	[436]
	MSN-ss-ADDA-TCPP	Disulfide-based Tat48-60, RGDS, ADDA, peptide-based amphiphile capping	Spherical	DOX	~ 120 nm	Targeting and GSH-responsive delivery of DOX to αvβ3 integrin overexpressed tumor cells	[317]
	HMSNs	TEOS and BTESPD with disulfide linkages	Hollow mesoporous shell	DOX	~ 100 nm	These constructs resulted in high loading capacity, and GSH-responsive controlled degradation	[293]
	Fa-PEG-MMSNs	Fa-PEG coated Mn^2+^-doped MSNs	Spherical	DHA	~ 100 nm	Accumulating PL-PUFA-OOH oxidized by ·OH and destroyed the structure of polyunsaturated fatty acids	[437]
	Au@MSN@HP NPs	HA, HS, and HP glycosaminoglycan modification	Core–shell	DOX	~ 100 nm	GSH-assisted degradation of the disulfide bond between GAG and MSNs favored precise synergistic chemophotothermal treatment	[438]
	PDA/MnO_2_ coated MSNs	PDA/MnO_2_ coating over MSNs	Spherical	DOX	150–300 nm	GSH-assisted transformation of MnO_2_ to Mn^2+^ led to the release of drug cargo	[439]
	FMSN-MnO_2_-BCQ	BSA-modified, NIR-II small molecule and MRI reporter	Fusiform/rod-like	MnO_2_ and CQ4T	width- ~ 15 nm, length- ~ 90 nm	TME-activated tumor-deep delivery system for dual-mode imaging and self-reinforcing chemodynamic therapy	[440]
Ultrasound-responsive	PV-MSNs	Platelet vesicles-coated over the surface	Spheroid	CA and IR780	100 nm	IR780-based SDT and the CA-based GSH depletion improved cancer ablation	[441]
	MSN-FA-TAN-MB	FA-immobilized over the surface	MB	TAN	~ 110 nm	This multifunctional vehicle showed exceptional ultrasound responsive properties towards tumor targeting and imaging	[442]
	FITC-labelled MSNs	Submicron cavitation nuclei	Spherical	Rhodamine B	–	Ultrasound-induced inertial cavitation enhanced the extravasation of the nanocarriers	[290]
	HYBRID_L_-PEG-RGD	Biotin or RGD peptide coated	Spherical	DOX	~ 220 nm	Ultrasound-responsive random copolymer enhanced cellular uptake and cancer-killing efficacy	[443]
	MSNs-PEG	PEG-coated over surface	Spherical	Gd(DTPA)^2–^	~ 92 nm	MRgHIFU stimulated cargo release facilitated by ultrasound-responsive PEG for MRI-guided therapy	[444]
	MNP@MSNs-AMA-CD	Bulky hydrophilic β-CD capping	Core–shell	DOX	~ 55 nm	HIFU-stimulated cleavage of ACVA C − N bonds facilitated the ultrasound-responsive release	[445]
Magnetic-responsive	EuSPION@MSNs	Polarization anisotropy (r) of two luminescence emission bands	Core–shell	–	–	Néel relaxation as the dominant heating mechanism resulted in understanding hyperthermia-based drug release	[296]
	SPNC@MSN	MnFe_2_O_4_@CoFe_2_O_4_ core and capped with Phe − Phe − Gly − Gly (N − C)	Core–shell	Fluorescein or daunorubicin	120 nm	Localized magnetic heating presented high cytotoxicity on pancreatic carcinoma cells	[295]
	MMSNs-PEG	PEG and thermoresponsive polymer-coated over the surface	Core–shell	DOX	160 nm	Heated magnetic species in the core facilitated the polymer transition and opening towards drug release from MSNs	[446]
	Fe_3_O_4_-mSiO_2_	Janus	Janus-type	Berberine	~ 300 nm	The superparamagnetic constructs with high drug-loading amounts, superior endocytic ability, and low cytotoxicity acted against hepatocellular carcinoma	[447]
	Mag@MSNs	Thermo-responsive polymer-coated core–shell MSNs	Core–shell	Fluorescein	55 nm	These core–shell constructs avoided the risk of inducing tumor metastasis generated by hyperthermia	[294]
	SPION@MSN	Retro-Diels Alder reaction DA, Mal, or CD	Sphere	Fluorescein	70–80 nm	Non-invasive external actuation through alternating magnetic fields improved the drug release	[448]
	MARS	ZnNCs Cucurbit[6]uril	Core–shell	DOX	< 200 nm	The non-invasive controlled delivery was achieved after being exposed to the AC field for treating breast cancer cells	[449]
Temperature-responsive	THI@HMS@P(NIPAAm-MAA)	P(NIPAAm-*co*-MAA)-coated HMSNs	Hollow MSNs	THI	~ 170 nm	The strongly temperature-dependent and distance-limiting mechanism was demonstrated using positive temperature coefficient pesticide	[450]
	MSNs-MNFs	P(NIPAAm-*co*-HMAAm)-encapsulated with MET-MSNs	Spheres in the electrospun nanofibers	MET	MSNs- < 100 nm MNFs-diameter of 420 nm	ON–OFF’ switching of AMF showed excellent heat generation efficacy and subsequent cytotoxicity on B16F10 melanoma cells	[451]
	MSN-PEG	RAFT polymerization of PEG	Spherical	Sulforhodamine B, PDI	140 nm	A temperature-controlled “pumping” mechanism was demonstrated for drug release from mesopores	[452]
	MSN-thermoresponsive polymer	Disulfide-containing cystamine linked thermoresponsive polymer	Spherical	DOX	50–100 nm	UCST polymers coated over the surface presented responsive release against breast cancer cells (SK-BR-3)	[283]
Light-responsive	Porphyrin capped-MSNs	Porphyrin capping	Spherical	RBP, TOP, or CAL	130 nm	Visible radiation-assisted generation of ROS-cleavable linkages allowed the release of TOP	[277]
	AuNPs-MSNs	AuNPs-capping with photoliable linker	Spherical	PTX	100 nm	Low power photoirradiation-assisted cleavage of linkers facilitated the zero premature release for chemotherapy	[136]
	UCNPs@mSiO_2_-DPP–CD	Strong host–guest interactions between CD and Ad	Core–shell–shell	DOX and platinum(II)	65 nm	Activating the platinum(IV), pro-drug gained higher toxicity effects of platinum(II)	[279]
	bMSNs-AZO/DS/CD-PMPC	AZO isomerization-modified surfaces	Core–shell	DS	150 nm	Light-responsive drug delivery and lubrication enhancement were beneficial for the treatment of osteoarthritis	[281]
	CuS@MSNs	CuS coated with MSN over the surface	Core–shell	DOX	86.2 nm	The carrier presented excellent combined NIR-based PTT and chemotherapy	[453]
	MSN-linker-azo/Ce6@Cargo@CD	CD-gated MSNs	Spherical	Rhodamine B or calcein	~ 100 nm	Excellent spatiotemporal controllability of red light excitation and the active target ligand FA improved efficacy of PDT and chemotherapy and controlled drug release	[278]
	MC/IR820-MSNs	Thermal-sensitive hydrogel platform MC/IR820	Hybrid hydrogel	DOX	~ 50 nm	These versatile photo-responsive hydrogels offered synergistic chemophotothermal treatment of OSCC	[454]
	FITC-PGSN	Polyglycerol-doped MSNs	Spherical	(Rose bengal, RB) FITC	~ 100 nm	TPA-PDT-assisted MSNs could transfer energy to the loading drugs via an intraparticle FRET mechanism	[455]
	FA-PEG–coated Ag-NPs-JNPs	FA-linked PEG-coated over the surface	Janus-type	ICG	200–400 nm	The effector for photothermal therapy acted as the initiator to activate the chemotherapy	[269]
Multi-responsive	MSN-S–S-DTPP&DTCPP	pH- and GSH-sensitivity	Spherical	DOX	~ 120 nm	Versatile dual-stimuli-sensitive MSNs could provide an effective strategy for combinational tumor therapy	[456]
	Serum albumin and myoglobin-gated UCNP@mSiO_2_	pH, GSH, or H_2_O_2_-responsive	Core–shell spherical nanostructures with worm-like pores in shells	DOX	64 nm	These nanocomposites showed spatiotemporally targeted drug delivery for cancer chemotherapy	[298]
	Dm@TMSN-PEI	Redox-enhanced pH-responsive	Spherical morphology with wormlike mesostructure	DOX and miRNA-145	~ 183 nm	The nanocomposites with affinity to glucose-regulated protein 78 (GRP78), a cell surface protein overexpressed in colorectal carcinoma is developed	[297]
	MSN-ANA-HFn	Redox- and pH-triggered	Spherical	DOX	100 nm	HFn capped MSNs provided TfR1 targeting on suppression of tumor growth	[299]
	TTTMSNs	pH- and redox-dual-responsive MSN-S–S-Peptide-MPEG	Rectangular	DOX	~ 125 nm	RGDFFFFC-assisted targeting, benzoic-imine bond-based pH-responsive, and di-sulfide cleavage-based redox-responsive enhanced the tumor-targeting efficacy	[457]
	MSN@p(NIPAAm-*co*-MA)	Thermal- and pH-responsive p(NIPAAm-*co*-MA)	Spherical	EVO and BBR	~ 160 nm	These composites with dual drugs provided excellent therapeutic effects against EMT-6 mouse mammary carcinoma tumor allograft	[261]
	MSNs@PDA@keratin	pH and GSH dual responsive Keratin as capping	Spherical/ellipsoidal	DOX	~ 100 nm	These composites selectively showed higher toxicity against A549 cells than normal cells	[301]
	MSN-SS-PDA	Redox/pH/NIR-multi-dependent, Disulfide linked PDA-coating	Spherical	DOX	~ 130 nm	These composites exhibited excellent photo-thermal conversion ability, multi-stimuli responsive drug release, chemo/photothermal synergistic therapy effect	[458]
	MSN-S–S-N = C-HA	pH- and redox-responsive HA-g-CD	Sphere with highly ordered honeycomb channels	DOX	~ 100 nm	The composites with dual-responsiveness provided CD44 over-expressed cancer cell targeting effects	[300]
	MSN-Au	GSH- and NIR-triggered AuNPs	Ellipsoid	DOX	~ 250 nm	A combination of chemotherapy and photothermal therapy toward A549 cells	[302]

β-CD: β-Cyclodextrin; ACVA: 4,4′-Azobis(4-cyanovaleric acid); AD- 1-Adamantanemethylamine; ADDA-TCPP: C12-CGRKKRRQRRRPPQRGDS; AMA: 1-Adamantylamine; AuNPs-Cys: _L_‐Cysteine‐derivatized gold nanoparticles; AZO: Azobenzene; BBR: Berberine; BFO: Bismuth ferrite; BTESPD: Bis[3-(triethoxysilyl)propyl] disulfide; CA: Cinnamaldehyde; CAL: Calcein; CDs: Carbon nanodots; Ce6: Chlorin e6; CendR: Neuropilin-1 (NRP-1)-dependent endocytic/exocytic transport; CM: Coumarin; CS-PtNPs@Zn-MSNs: Chitosan-Platinum nanoparticles coated Zinc-doped MSNs; CuS: Copper sulfide; Cu-Fe-MSNs Copper and iron-doped MSNs; DACHPt: Activated oxaliplatin (1,2-diamminocyclohexane platinum(II); DHA: Dihydroartemisinin; DMSNs-dendritic MSNs; DOX: Doxorubicin; DS: Diclofenac sodium; EVO: Evodiamine; EuSPION: Europium-doped superparamagnetic iron oxide nanoparticle; FA: Folate; FaPEG: Folate-grafted PEG; FCA-Fe_3_O_4_ coated carbon/silver; FITC: Fluorescein isothiocyanate; FRET: Fluorescence resonance energy transfer; Gd(DTPA)^2–^: Gadopentetate dimeglumine; GSH -glutathione; HA: Hyaluronic acid; HAp: Hydroxyapatite; Hfn: Human H chain ferritin; HMAAm: *N*-hydroxymethylacrylamide; HMSiO_2_ /HMSNs/HMS: Hollow mesoporous silica nanoparticles; HNPs: harmonic nanoparticles; HP: Heparin; HS: Heparin sulfate; HYBRID: Hybrid mesoporous silica nanocarrier; IBU: Ibuprofen; ICG: Indocyanine green; INF-Interferon; IL-Interleukin; JNPs: Janus-type MSNs; LB-MSN-OVA: Lipid bilayer-MSN-ovalbumin; MA: Methacrylic acid; Mal: Maleimidopropyl triethoxysilane 1; MARS: Magnetically activated release system; MB: Microbubble; MC: Methylcellulose; MET: Metformin; miRNA: MicroRNA; MMSNs: Manganese-doped MSNs; MNFs: Magnetic nanofibers; MNPs: MnFe_2_O_4_@CoFe_2_O_4_ nanoparticles; MRI: Magnetic resonance imaging; MRgHIFU: MRI-guided high-intensity focused ultrasound; mSiO_2_-mesoporous silica; MSNs- mesoporous silica nanoparticles; MSN-POLY: RAFT polymerization on the surface of MSNs; NIPAAm: *N*-isopropylacrylamide; OG: Oregon green; OSCC: Oral squamous cell carcinoma; OVAp: Ovalbumin; PAA: Polyacrylic acid; PDA: Polydopamine; PEG: Polyethylene glycol; PEI: Polyethylenimine; PGSN: Polyglycerol-doped MSNs; PL-PUFA-OOH: Lipid peroxides; PMPC: Poly(2-methacryloyloxyethyl phosphorylcholine); p(NIPAAm-co-MA) : Poly(*N*-isopropylacrylamide-co-methacrylic acid); PV: Platelet membrane vesicle; NAC: *N*-acetyl-l-cysteine; SDT: Sonodynamic therapy; siRNA: Small interfering RNA; SPNC: Superparamagnetic nanoparticle cores; TAN: Tanshinone IIA; TEOS: Tetraethyl orthosilicate; TfR1: Transferrin receptor 1; THI: Thiamethoxam; THPMP: 3-trihydroxysilyl propylmethylphosphonate; TOP: Topotecan; TPA-PDT: Two-photon activated-photodynamic therapy; TPP: Triphenylphosphine; Trp: Tryptophan; pDNA: Plasmid DNA; PV-coated MSNs: Platelet vesicles-coated MSNs; RB: Rose bengal; RBP: [Ru(bipy)_3_]Cl_2_; RITC: Rhodamine B isothiocyanate; ROS: reactive oxygen species; UCNPs: Upconversion nanoparticles; USMO: Ultrasmall manganese oxide; V7-RUBY: Wormhole mesoporous silica nanoparticles; YQRLGC: lysosomal sorting peptides; WS_2_-HP: Tungsten disulfide quantum dots; ZnNCs: Zinc-doped iron oxide nanocrystals

##### pH-responsiveness

Typically, the infectious and cancerous sites, including intracellular and extracellular regions, possess lower pH levels (4.5–6.0) than the surrounding physiological fluids (the blood, pH of 7.4). More often, the designed nanoplatforms are passively targeted and accumulated due to the EPR effect based on the leaky vasculature, specifically, solid tumors. In this context, the delivery systems upon internalization through endocytosis face an extreme shift in the pH value to 6.0 in the surrounding medium of early endosomes, which gradually reduces to pH-4.5 in late endosomes and lysosomes due to the excess availability of proteases and hydrolases, as well as supply of protons into their lumen as a part of defense mechanisms against foreign bodies. A simultaneous shift in pH of the environment could be used as an effective biological trigger, favoring the release of therapeutic guest molecules, specifically anticancer and antimicrobial drugs. This potent triggering effect of responsiveness by specific functionalities immobilized on the advanced nanocontainers could address numerous issues associated with the conventional approaches, such as premature leakage of drugs, combating MDR toward effective therapy [247]. Several examples of specific functional groups include ionizable weakly acidic/basic functional groups, such as hydrazone, disulfide linkage, and dinitroimidazole, among others [14, 248]. In addition, some of the currently available polymers contain pH-responsive linkers, such as chitosan and dextran from the natural origin, as well as carboxylic and amine functional groups containing polymers from synthetic origins. More often, these pH-responsive moieties are applied by conjugating them in the mesopores or coated over the surface for immobilizing the therapeutic molecules for triggered release either alone or through a capping agent [74, 114, 127, 143, 226, 249–252].

**Fig. 5:**
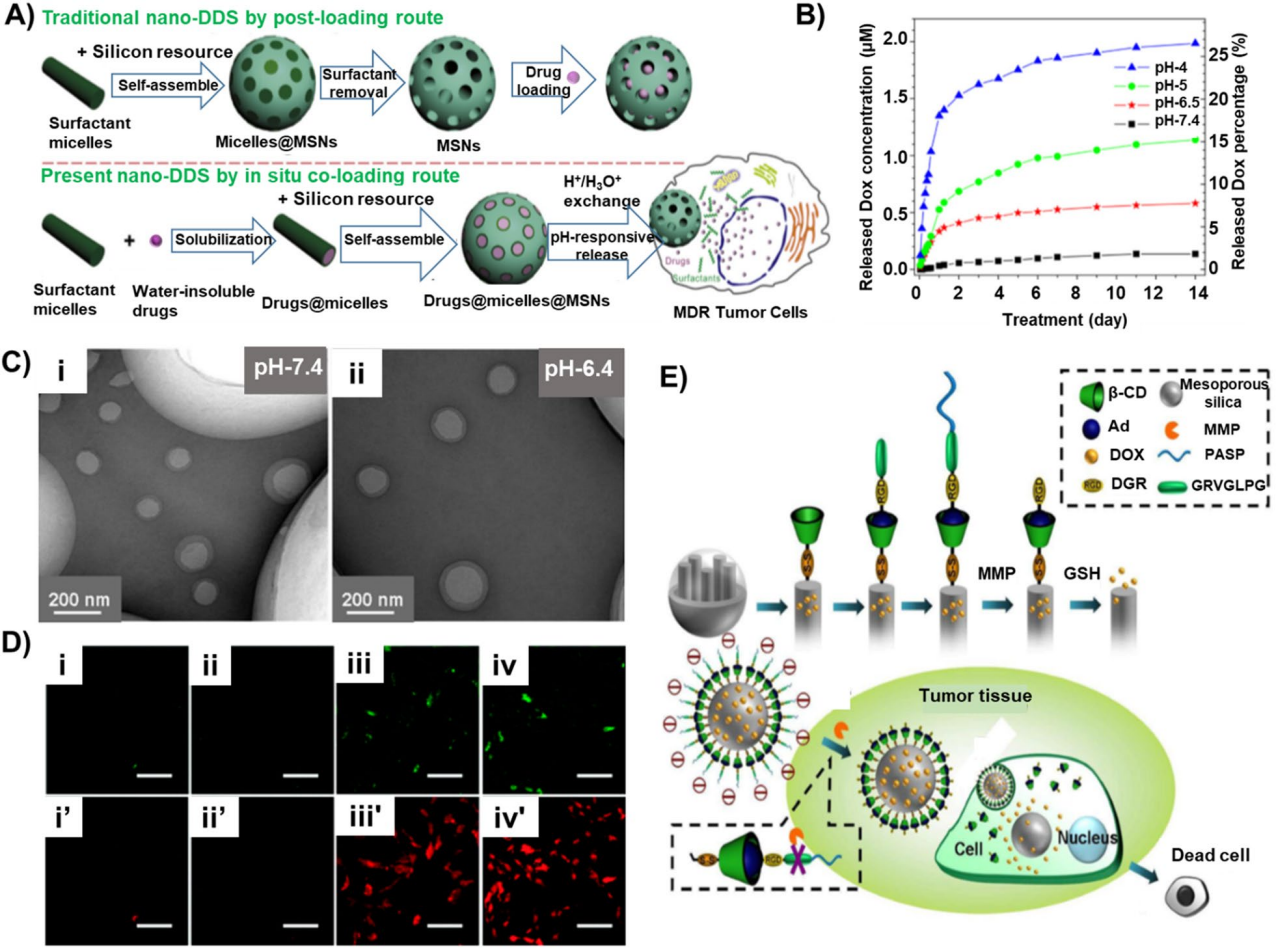
Various advanced MSNs for pH-responsive delivery of drugs. **A** Methodological comparison between the traditional MSNs-based nano-drug delivery systems by the drug post-loading route and the proposed MSNs by the drug in situ co-loading route. **B** In-vitro pH-responsive drug release behaviors of DOX@CTAB@MSNs in the release media of different pH values, which were used to simulate the alkalescent conditions in normal tissues and blood (pH ~ 7.4) and the acidic conditions in the tumor (pH = 4–6.8). Reproduced with permission from Ref. [76] Copyright 2011, Elsevier. **C** TEM images of nanomicelles with (i) and without (ii) PEG segments at different pH values. **D** Confocal microscopic images of HeLa cells treated by rhodamine B-loaded and FITC-labeled micelles for 4 h at different conditions: (i) pH = 7.4, 37 °C; (ii) pH = 7.4, 39 °C; (iii) pH = 6.4, 37 °C; (iv) pH = 6.4, 39 °C (scale bar: 80 μm). Reproduced with permission from Ref. [253] Copyright 2010, American Chemical Society. **E** Schematic showing the fabrication of MSNs for tumor-triggered targeting drug delivery. Functionalization protocol of the MSN; drugs loaded under physiological condition; removal of PASP protection layer in response to MMP at a tumor site; cell uptake through RGD-mediated interaction; GSH-triggered drug release inside the cell; and apoptosis of tumor cells. Reproduced with permission from Ref. [254] Copyright 2013, American Chemical Society

The advanced MSNs with tunable morphology and appropriately immobilized functional groups exhibit precise pH-responsiveness in attaining the advanced delivery and exceptional cancer therapy performance. In this vein, several studies have been reported by utilizing various pH-responsive linkers in the efficient delivery of therapeutic guest molecules, specifically at the targeted tumor and infection sites [116]. In one case, He et al. demonstrated the release of CTAB and doxorubicin (DOX) from positively-charged micelles/MSNs composites through interactions with the proton, specifically in a high proton environment compared to traditional post-loaded drug systems for overcoming MDR in cancer (Fig. 5A, B) [76]. The interesting feature of this design included a facile single-step synthesis, avoiding the surfactant removal and drug encapsulation procedures separately. In another case, the surface-coated PEG protective layer over the MSNs could be detached at a pH lower than 6.8, for instance, the cancer microenvironment. The encapsulated MSNs were subsequently internalized by the cancer cells with the identification of the RGD motif (Fig. 5C, D) [253]. Similarly, Zhang et al. fabricated versatile envelope-type MSNs to achieve pH-triggered, tumor-targeted delivery of DOX [254]. Further, upon the hydrolysis of matrix metalloproteinase substrate peptide, the polyanion outer layer was eliminated, resulting in the exposure of the RGD motif. Moreover, the GSH-assisted disintegration of disulfide, along with the improved uptake of tumor cells, significantly contributed to the rapid drug release through cyclodextrin gatekeeper (Fig. 5E). In this regard, various polymers, for instance, chitosan, PEI, and PEG, PAA [111, 251, 255–259], and lipids [109, 260, 261], as well as the mixture of polymers and lipids [262, 263], have been coated over the MSNs for the pH-responsive delivery of chemotherapeutics from MSNs. These complex designs significantly facilitated versatile functionalities eventually, resulting in the apoptosis of the cancer cells. Although the design presented multiple successful functionalities, the multiple immobilized groups require multiple-step synthetic procedures, which could substantially limit the design in bulk manufacturing and compatibility explorations. Moreover, the eventual tracking of performance efficacy and exploring the pharmacokinetic behavior of the MSNs-based complex designs are further challenges to be addressed.

In the vein of addressing the establishment of innovative therapeutic modalities against cancer therapy, most studies have focused on the establishment of pH-responsive linkers for drug delivery and coating of pH-responsive polymers for capping of MSNs towards successful intracellular delivery [264]. These innovative modifications have successfully addressed cancer therapy to a considerable extent, including delivering chemotherapeutics and overcoming the drug resistance towards improved biological performance [243, 265]. Despite the delivery success, the complete eradication of cancer remained a significant challenge as most solid tumors possess tight junctions with impermeable barriers, limiting the entry of nanomedicines into deep tumors and failing their clinical translation [50]. Over the years, these highly complex anatomical features of tumors resulted in unsuitable therapeutic strategies, resulting in the ablation of surface tumor cells alone, and the deep tumor led to recurrence. To a considerable extent, several surface modification strategies have been employed to address the deep penetration via piercing the tight junctions of tumors through surface charge reversal and size reduction, among others [50, 266]. In this regard, MSNs and their advanced prototypes have been employed to address the deep tumor penetration using the surface-modified MSNs. In a case, MSNs were coated with the up/downconverting luminescent nanoparticles (U/DCNPs) via acid-labile (benzoic-imine) linkages to form core–shell assemblies and subsequently coated with the charge reversible polymers, poly(allylamine)-dimethyl maleic anhydride–polyethylene glycol (PAH-DMMA-PEG) [266]. The charge reversal of coated polymer from negative to positive in the acidic microenvironment induced the disintegration into isolated MSNs and UCNPs, facilitating the successful penetration into solid tumors. In another case, Mannose-decorated MSNs were coated with the PDA-Gd-PEG composite for MRI and deep penetration through charge reversal for synergistic photothermal and visualized therapy [267]. Although the success in the fabrication of these innovative constructs, in several instances, the degradation of the resultant MSNs after degradation of the surface-modified organic components are the further challenges remained to be addressed. In an attempt to address this aspect to a considerable extent, our group has demonstrated the fabrication of the nanoplatforms based on the pH-responsive chitosan-encapsulated with Pt nanoparticles coated over the metal-impregnated MSNs [110]. The decoration of ultrasmall Pt-based nanoconstructs facilitated the advanced therapeutic capabilities of deep tumor penetration through influencing the adherens junctions between the tumor cells. Interestingly, the metal-impregnated MSNs, certainly showed the degradation of MSNs due to low silica density and coordination arrangement of metal species in the MSN framework. However, further studies of addressing the controlled degradation ability and monitoring the resultant byproducts remained to be explored.

##### Light-responsiveness

**Fig. 6:**
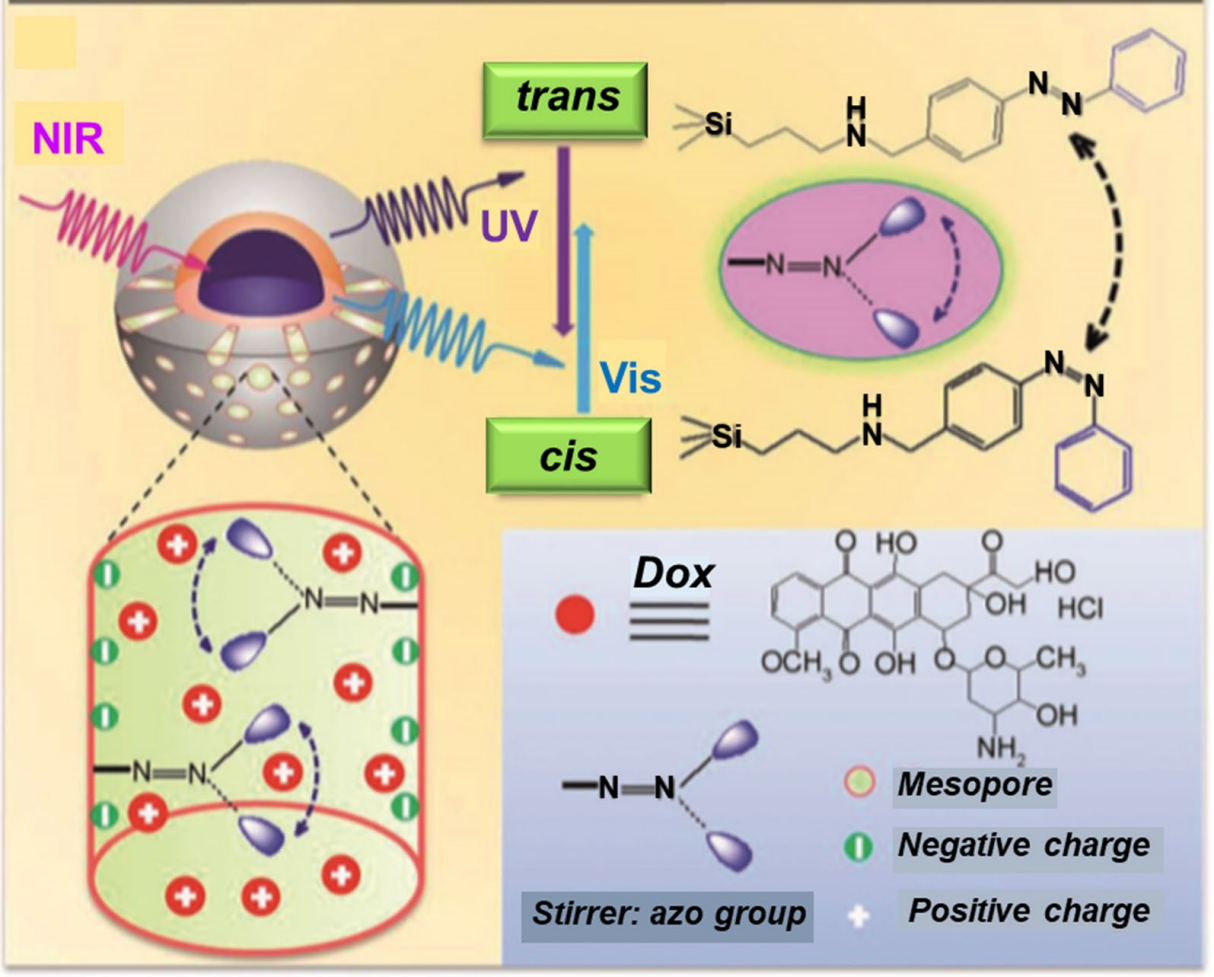
Light-responsive advanced MSNs. NIR light-triggered DOX release by using the upconversion property of UCNPs and trans–cis photo-isomerization of azo molecules grafted in the mesopore network of MSNs. Reproduced with permission from Ref. [272] Copyright 2013, John Wiley and Sons

The light-based therapies, i.e., photodynamic therapy (PDT) and photothermal therapy (PTT), offer several beneficial qualities, such as selectivity, preferential localization of photosensitizer at the tissue of interest, and substantial activation execute the therapeutic function, among others [268, 269]. However, severe aggregation of photosensitizers due to hydrophobicity is one of the critical issues of such light-induced therapies, limiting their applicability [269, 270]. To a considerable extent, the utilization of various carriers with adequate carrying capacity enables the successful transportation of photosensitizers to the desired sites and substantially facilitates their clearance from the body [271]. Despite the limited efficacy and vulnerable damage by ultraviolet (UV) radiation, it is highly desirable to establish NIR light to avoid that quick attenuation, which further prevents the biological samples and living organs tenuously from being destructed due to its more profound tissue penetration efficacy [252, 272, 273]. Investigations on light (visible/UV/NIR)-induced therapeutic efficacy using MSNs-based nanocarriers have been carried out, attributing to the light-triggered characteristics in a spatiotemporal manner [129, 136, 140, 268, 274–276]. In a case, a novel drug carrier was fabricated based on MSNs coated with porphyrin caps grafted with ROS cleavable bonds [277]. The visible light, i.e., common light,-assisted cleavage of decorated porphyrin caps could release the encapsulated drug cargo, topotecan, from MSNs. The highly compatible light and dual effects of ROS production, in terms of triggered release and therapeutic effects, substantially demonstrated the antitumor effects in HOS cells. Although the design is advantageous, several performance- and compatibility-related attributes in-vivo yet remain to be explored as the light penetration and depth of tumor may hinder the efficacy of light exposure. Moreover, the comprehensive evaluations of type of light utilization, exposure time, and distance, must be selectively optimized. In this regard, several studies with some smart linkers, such as azo-isomerization, and light-assisted opening of gatekeepers, enabled the fabrication of various intelligent nanocarriers using MSNs [278–281]. In another case, Liu et al. fabricated MSNs-based upconversion core–shell nano-constructs, which were highly appealing toward the exertion of NIR light [272]. Considering the fascinating feature of photo-triggered luminescence ability, the cancer-therapeutic drug (DOX) was loaded in the mesopores of silica constructs, in which azobenzene (azo) group is selected as “stirrer” (Fig. 6), on absorbing the NIR light (980 nm), the nanoparticles significantly transmitted photons in UV band that instantly absorbed by the light-responsive azo group. Further, the isomerization generated the cyclic movement of rotation-inversion, promoting the release of DOX as an impeller via simultaneous UV–Vis light emission. The interesting switching of light frequencies by cyclic movement of rotation-inversion would be convenient in exploring through the in-vitro investigations, which, however, is undoubtedly challenging in executing in in-vivo as the tissue microenvironment is highly complex.

##### Thermos-responsiveness

Indeed, the environmental temperature is another fascinating and promising stimulus, in which the controlled increase of the temperature at the diseased site may substantially trigger the controlled release of therapeutic guests. In this context, various spatiotemporal thermos-responsive units have been immobilized in these MSN-based architectures, exhibiting an exciting feature of reversibly responding to temperature, such as pNIPAAm, and sulfobetaine methacrylate (SBMA), or depositing specific metal species like graphene oxide or Pt nanoparticles [176, 252, 282–285]. Instinctively, the system is operated based on a reversible switching-on/off mechanism at the mesopore entry via diverse internal or external decoration or modification over the surface. These composites at elevated environmental temperatures exhibit phase transition, enabling the release of drugs through changes in their hydrophobic interactions [284, 285]. More often, the thermogelling polymers, such as pNIPAAm, present an exciting feature of gelation at elevated temperatures, such that they facilitate the localized delivery of drugs in a controlled fashion [285]. Several reports have focused on manipulating temperature-induced drug release, showing a tremendous challenge to distinguish between fatal tumor sites and healthy peripheral tissues [286]. For instance, Sun et al. demonstrated the novel temperature-responsive polyampholyte-coated MSNs by reversible addition-fragmentation chain transfer polymerization on their surface for the first time, displaying the controlled release behaviors and high biosafety [282]. In another case, Tian et al. applied a thermo-sensitive polymer (pNIPAAm) and coated it over SBA-15-type silica to synthesize an ordered mesostructure with the low critical solution temperature collated [287]. Notably, different from the capping agents acted by super valves, the flexible polymeric shells over MSNs offer significant advantages over agglomerating and providing other partitions for cargo loading apart from the pores of MSNs [252, 283, 284]. Apart from the delivery through thermos-responsiveness, specific light-responsive molecules, such as indocyanine green (ICG), could facilitate the synergistic therapeutic efficacy through augmented temperatures, referred to as hyperthermia, at the tumor sites, resulting in the substantial ablation through singlet oxygen generation. Notably, the MSNs-based advanced nanosystems with synergistic light- and temperature-induced ablation offer more advantages over the single-responsive system.

##### Ultrasound-responsiveness

**Fig. 7:**
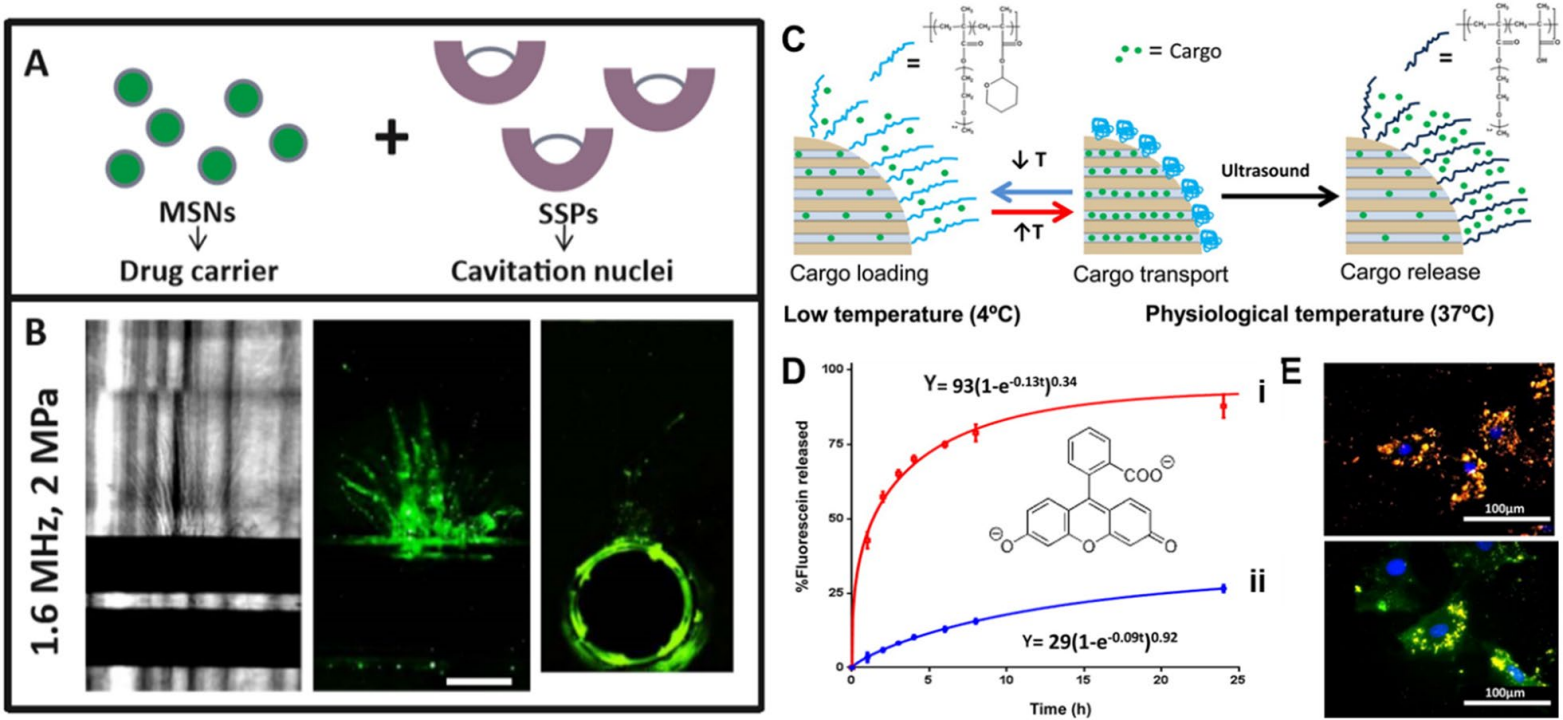
MSNs-based ultrasound responsive drug delivery. **A** Schematic of combining MSNs with SSPs. **B** Representative microscopy images of nanoparticle delivery in the agarose phantom model (MSN in combination with SSPs), showing bright field (left), green fluorescence (center), and green fluorescence in a cross-section of the flow channel (right) (n = 3). Scale bar = 500 µm. Reproduced with permission from Ref. [290] Copyright 2017, Elsevier. **C** Schematic illustration of the behavior in an aqueous medium of the dual responsive release system. **D** Release profiles of fluorescein from hybrid-MSNs in PBS solution versus time with ultrasound exposure (10 min and 1.3 MHz, 100 W) (i) and without ultrasound (ii). **E** Fluorescence microscopy images of LNCaP cells incubated with Rhodamine B-labeled hybrid-MSNs with fluorescein loaded before (top) and after (bottom) ultrasound irradiation. Reproduced with permission from Ref. [17] Copyright 2015, American Chemical Society

The ultrasound-based responsiveness acts by applying a higher frequency wave that exceeds human auditory limitations. The sonochemical process is classified based on frequency and penetration depth. In general, the low frequency (< 1 MHz) shows a superior penetration depth irrespective of the site. In comparison, high (5–10 MHz) and medium (1–5 MHz) frequencies display inferior penetration capacity along with the destruction of living tissues [288]. Regarding the biomedical applications, the approach has recently garnered enormous attention towards tumor ablation, either by mechanical or thermal methods conducted on subcellular disruption or coagulative necrosis, respectively [289]. Inertial acoustic cavitation concept by the oscillation raised from fluidic gas bubbles, either linear or non-linear related, has been acknowledged as the most significant mechanical ultrasound effect for biomedical applications [276]. Nevertheless, an unstable phenomenon exists of the drastic collapse of the growing bubbles under the fluidic inertia. In one case, interest on drug carriers and nuclei for inertia cavitation stimulation, Paris et al. enhanced the extravasation of MSNs with the combination of acoustic-sensitive polymeric particles, resulting in the initiation of inertia cavitation process by reducing the required pressure under the focused ultrasound conditions (Fig. 7A, B) [290]. In another study, they fabricated ultrasound-sensitive MSNs, presenting significant effects by controlled release of encapsulated therapeutic guests through gate-opening strategy [17]. The coated polymer over MSNs was collapsed initially and then opened its conformation like a coil after the hydrophobicity change, triggering the cargo release with the preservation of ultrasound-sensitive capacity (Fig. 7C–E). In-depth analyses are required to establish the mechanistic studies that are yet to be explored.

In addition to the notified most common stimuli triggers (pH, light, and thermos), various advancements have been evidenced in employing advanced MSNs that are responsive to other stimuli, such as GSH-, and magnetic-responsive. More often, the internal trigger, GSH-responsive, linkages are decorated either on the surface of MSNs or in the siliceous frameworks, for instance, di-sulfide-linkages [291, 292]. The decoration of GSH-responsive linkers containing polymers on the surface act as gate keepers, facilitating the release of the encapsulated therapeutic cargo from the mesopores [291]. In addition, such responsiveness can be employed to encapsulate the drugs or genes for controlled release, specifically in the GSH-rich environment, i.e., cancers [292]. In most instances, the GSH-responsive disulfide linkages were bridged in the organosilica frameworks towards fabricating the biodegradable MSNs [41, 83, 293]. These smart disulfide bridged MSNs not only present the successful delivery of the therapeutic guests in the intracellular environment precisely but also offer the impressive and required biodegradability attribute. To this end, the MSNs encapsulated with the magnetic species, for instance, iron oxide, as core–shell architectures are often employed for the localized delivery using the externally applied magnetic field [294–296]. Apart from a single trigger, several studies have explored the utilization of multiple stimuli (two or more) either to control the release of therapeutic guests by unlocking the capping or the conveyance of the MSNs [297]. In this regard, various multiple stimuli combinations employed in the fabrication of advanced prototypes of MSNs include pH, GSH, or H_2_O_2_-responsive [298], redox-enhanced pH-responsive [297, 299], thermos- and pH-responsive [261], pH- and GSH-responsive [300, 301], GSH- and NIR-triggered [302] nanocomposites. These composites with multiple stimuli-triggered delivery or degradation offer more advantages than the single stimuli-based composites. However, they require various mechanistic elucidations and comprehensive evaluations in orchestrating their synergistic effects for their successful translation to clinics.

#### Metallic linkers

Despite the advantageous morphological attributes and well-defined porous architectures, it is evident that the traditional siliceous frameworks can be supported only as carriers concerning the drug delivery application [42, 43]. However, specific modifications according to the requirements often lead to enhanced performance and substantial applicability of the carrier. In addition to numerous aforementioned molecular and supramolecular stimuli-responsive constituents [13, 33, 243, 249, 265, 303], various transition metal species with exceptional potential can be employed to establish the responsive linkages within the siliceous frameworks due to their unique electronic properties and structural attributes [220, 304, 305]. Establishing the transition metals (Cu, Fe, Co, and Zn)-based functional coordination linkages offer enormous advantages over traditional MSNs in drug delivery, in terms of improved drug loading efficiency, precise control over their on-demand release, and controlled degradability. In recent times, lanthanides (La^3+^ ions) have also been doped into the siliceous frameworks for drug delivery applications, which, however, require further optimization and safety considerations comprehensively [306]. In addition to safety issues, the loading efficiency of therapeutic guests within the porous containers must be addressed as it explores the therapeutic levels at the target site to perform its therapeutic action and eliminate the irregularities in the dosage frequency.

**Fig. 8:**
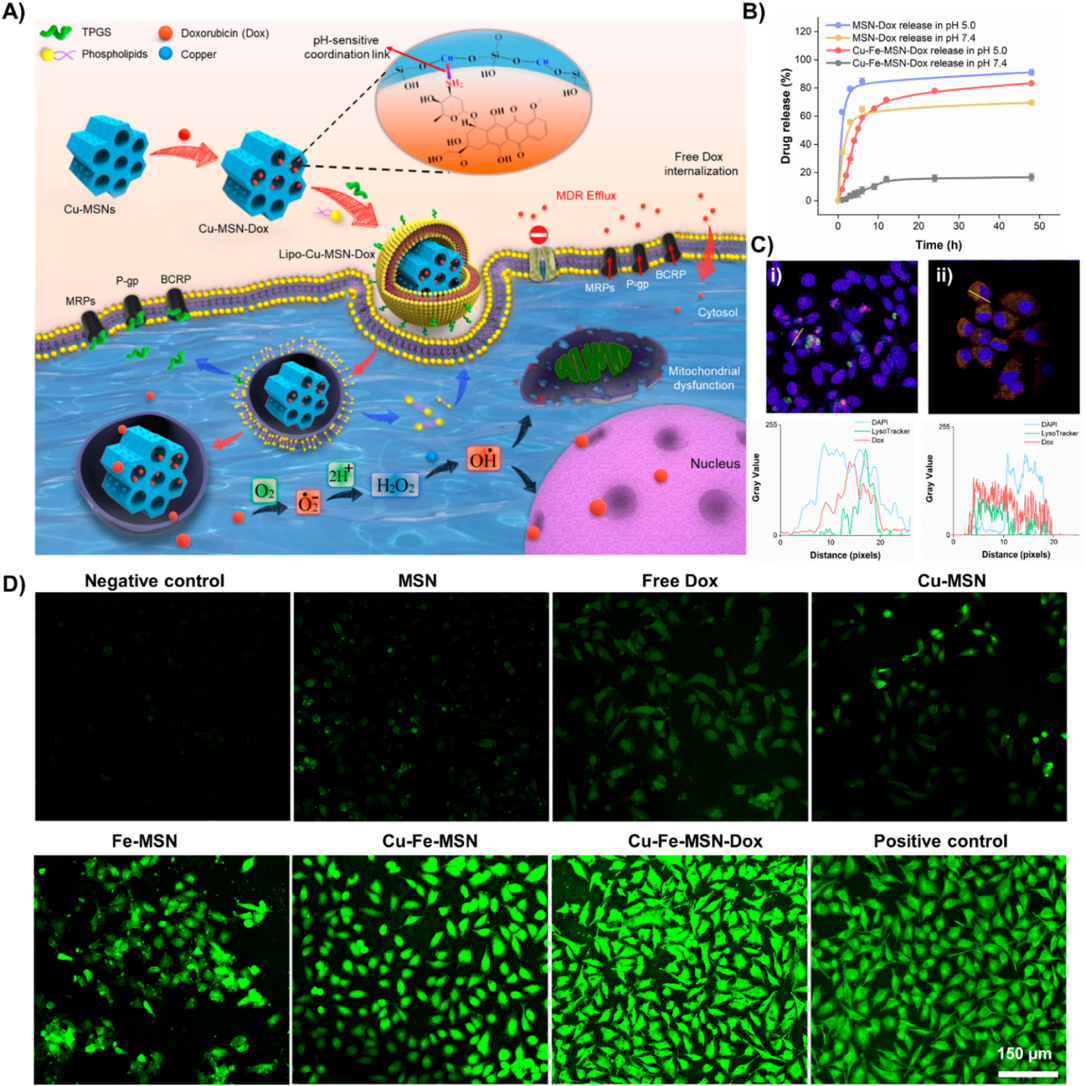
Metallic linkers in MSNs for diverse applications. **A** Schematic illustration showing the synthesis and cell internalization of designed hierarchical metal-impregnated MSN nanoformulation elucidating the delivery of DOX and plausible mechanism of surpassing MDR. Reproduced with permission from Ref. [82] Copyright 2017, American Chemical Society. **B** pH-responsive release of DOX from Janus-type (Cu-Fe-MSNs) architectures at various time intervals in simulated fluids, PBS at pH 5.0 and 7.4. compared to naked MSNs. **C** Cellular internalization illustrating the lysosomal escape of free DOX and Janus-type nanocontainers (Cu-Fe-MSN-DOX) in HeLa cell line and their respective RGB fluorescence intensity profiles of DAPI, LysoTracker, and DOX at the selected region (yellow line) from the samples (i) Free DOX, and (ii) Cu-Fe-MSN-DOX for 2 h, respectively. **D** CLSM images showing the ROS levels correlating to DCF-DA fluorescence in HeLa cells after treatment with bare MSNs, Cu-MSNs, Fe-MSNs, Cu-Fe-MSNs, and Cu-Fe-MSN-DOX at 80 μg/mL for 3 h. Reproduced with permission from Ref. [92] Copyright 2019, Elsevier

Previous reports indicated that the loading efficiency of the therapeutic guest molecules in the MSN pores is significantly poor as the affinities between the guest and host predominantly depend on the physical adsorption by weak hydrogen bonding or other electrostatic interactions, leading to a simultaneous exchange with the ions in the surrounding medium [26, 116, 307]. In addition, these weak interactions often result in premature loss of therapeutic molecules not only during the encapsulation but also while conveyance through physiological fluids, resulting in deprived therapeutic efficiency due to their insufficient levels at the desired site. To a considerable extent, these issues have been addressed by installing various stimuli-responsive capping molecules, such as the utilization of quantum dots [68, 250], AuNPs [139, 145], and protein or polymer assembly [88, 89, 97, 127, 259]. However, it should be noted that these complex strategies require multi-step synthesis, leading to mechanical abrasion-induced changes in MSN shapes [92]. In an attempt to address this issue, we have done enormous work by fabricating innovative metal-encapsulated MSNs through a single-step, facile approach by impregnating positively-charged first-transition-row metals species viz*.* Cu metal species in the mesoporous frameworks [82, 96]. These metal species in the frameworks offered coordination linkages with the therapeutics (herein, DOX molecules), resulting in their improved loading efficiency compared to the pristine MSNs (Fig. 8A) [82, 96]. In addition to improved loading efficacy, these specific interactions were highly susceptible in the acidic environment (pH-5.0) and facilitated disassembling through the protonation of nitrogen atoms in the guest molecules (Fig. 8B, C) [96, 308]. Despite the efficient loading and precise release of the encapsulated guest species, the doped metal species could merely facilitate the coordination interactions to certain guest species with terminal amine-functional groups [82]. Further efforts in terms of the incorporation of various metals, such as Fe, Zn, have been reported [91, 110]. Hitherto, only very few metals have been doped, requiring enormous investigations to further explore the multiple combinations and optimized conditions for incorporating diverse metal species. Although reduced silica density improves the compatibility attributes, the safety and toxicity considerations must be considered while doping various metals in siliceous frameworks.

Compared to molecular and supramolecular linkers, these transition metal-based MSNs offer a specific advantage of participating in the metabolic activation cascade reactions due to the unique electronic architecture. These metal species, such as Cu and Fe, participate in catalysis and significantly facilitate the conversion of the intracellularly available H_2_O_2_ molecules to toxic ROS. These deadly free radical species promote the substantial activation of apoptotic cascades and cell membrane deterioration, leading to cell death. In an attempt to explore these events, we fabricated the Cu metal-impregnated and multi-metal (Cu/Fe)-doped MSNs, which resulted in the generation of highly toxic free radicals by participating in the redox chemistry mediated by chemodynamic therapy (Fig. 8D). These deadly species damaged the intracellular organelles, such as mitochondria, by disturbing the electron transport chain for energy production and substantially enhancing the stimulation of various apoptotic cascades. Notably, the rationale behind choosing these metals was that they could participate actively in the redox reactions and lead to the generation of higher amounts of free radicals from H_2_O_2_ molecules, which were in higher amounts in cancer cells over normal cells. Moreover, these metals would cause no adverse effects and are regarded as safe as these metals are essential trace elements in the body. Moreover, incorporating various metals, being one of them as Fe metal, facilitated the change of the overall shape of MSNs to sphero-ellipsoid due to the dependent arrangement of positively-charged metals by strong repulsion forces and played a significant role in the tumor ablation through Ferroptosis compared to the free DOX molecules. Despite the success, further in-depth analysis in biosafety and delivery efficiency is required. Moreover, impregnating different combinations of the metals with substantial altered physicochemical features would inevitably provide the synergistic effects in multiple aspects of improved optical and electrical properties as well as therapeutic events. However, the critical advancements in the utilization of various metal species would result in severe effects on the elimination rates imposing severe health risks, requiring in-depth analysis for addressing these issues.

#### Targeted delivery of chemotherapeutics

**Fig. 9:**
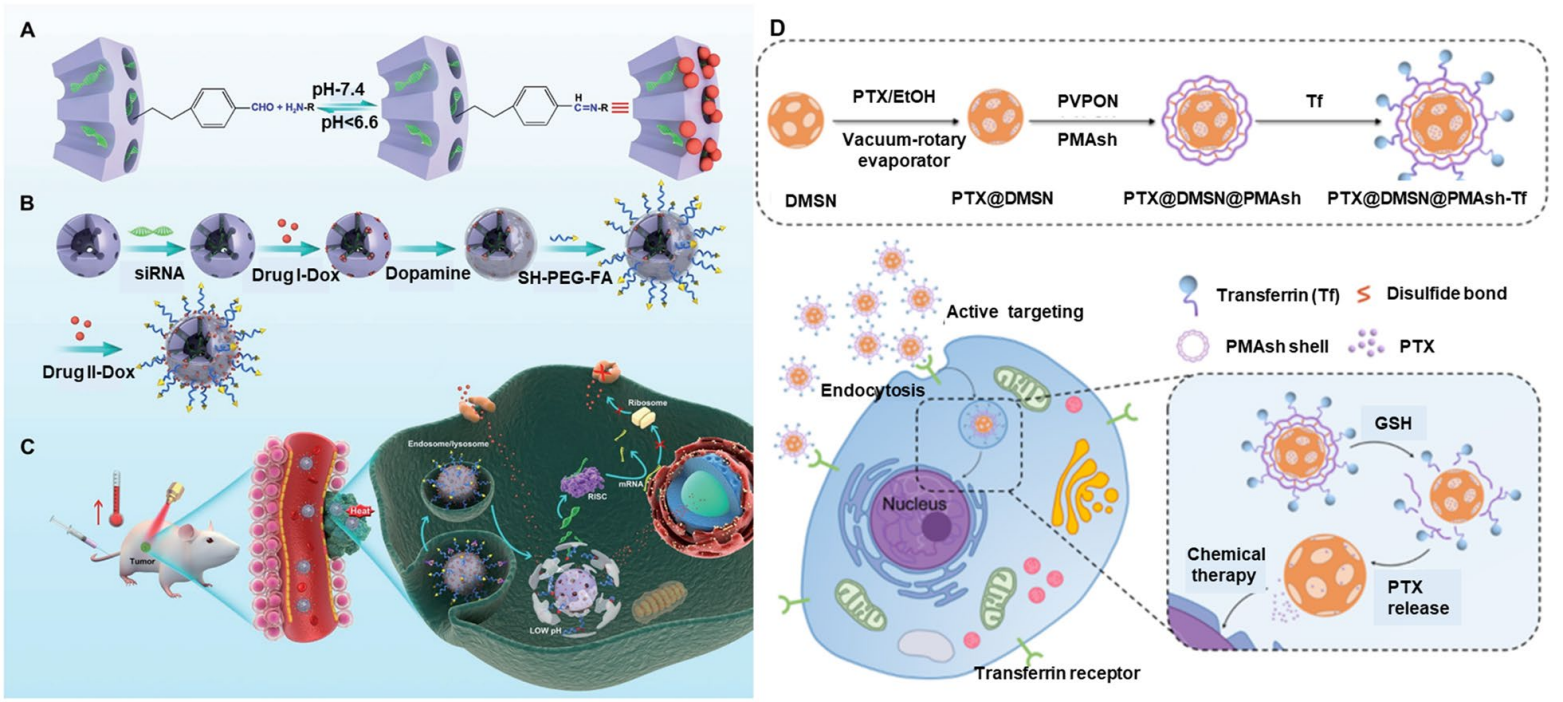
Schematic illustration of **A** the dynamic interaction between DOX and benzaldehyde via pH-sensitive benzoic–imine bond. **B** The synthesis route of M-R@D-PDA-PEG-FA-D and **C** the combined photothermal chemo gene-targeted therapy of tumors. Reproduced with permission from Ref [318]. Copyright 2017, John Wiley and Sons. **D** Schematic illustration of the synthesis and cancer cell internalization of the PTX@DMSN@PMAsh-Tf nanocarriers with both tumor-targeting and GSH-responsive deconstruction of the protective PMAsh shell for the efficient delivery of water-insoluble PTX in cancer therapy. Reproduced with permission from Ref. [232] Copyright 2021, Elsevier

More often, the advancements based on pristine MSNs and their subsequent composites have been predominantly focused on various stimuli-controlled nanoformulations towards improving the encapsulation and delivery efficiencies. Although successful in terms of controlled delivery avoiding premature leakage, most of these formulations lack targeting ability as they are simply dependent on the EPR effect (loose endothelial/vascular connections facilitate their entry specifically in cancer), hampering their translation to clinics [309]. Despite the advantageous attributes to a considerable extent, the efficacy of the EPR effect-based passive targeting was questioned due to inappropriate accumulation, off-target toxicity, and development of MDR, among others [310]. To this end, several active targeting approaches of MSN-based nanocomposites have been designed using various targeting ligands, such as folic acid (FA), prostate-specific membrane antigen (PSA), HA, lactoferrin, RGD, transferrin, and lactobionic acid, among others [309, 311–316]. These ligands immobilized either on the surfaces directly or through the polymer conjugation specifically act by targeting the overexpressed corresponding receptors on cancer precisely to increase the internalized delivery and reduce adverse events in normal cells. In addition to these small targeting molecules, several long-chain multifunctional peptides have been decorated to improve tumor-targeting efficacy [145, 317]. The external stimuli-based targeting efficacy can also be employed to precisely target the designed nanoformulations, for instance, external applied magnetic field [7]. Further efforts have been continued to explore diverse targeting strategies for precise cancer therapy. In a case, Cheng et al. fabricated a multifunctional platform based on the advanced prototypes of MSNs encapsulated with siRNA and DOX species, gated with the PDA and SH-PEG-FW coatings subsequently for targeted delivery (Fig. 9A**-**C) [318]. These nanoconstructs encapsulated with DOX and siRNA further enhanced the cytotoxic effects in MCF-7/ADR cells through overcoming the resistance, resulting in the siRNA-medicated downregulation of P-gp inhibition and retention of DOX. In another case, Gisbert-Garzarán et al. fabricated biotin-conjugated MSNs, which were first immobilized with the silylated redox-responsive linker and further functionlized with the thiol-PEG-NH_2_ [319]. The targeted and redox-responsive delivery platform presented the controlled premature drug release and subsequent targeting to the tumor, inducing specific and enhanced internalization by HAVAB moieties into the tumor spheroids. Further, the designed nanocomposites showed excellent tumor reducing efficacy in chicken embryos, which, however, improved the internalization through a passive accumulation in tumors. Similarly, the transferrin ligand-conjugated dendritic large-pore MSNs through the poly(methacrylic acid) shell, for the delivery of water-insoluble paclitaxel for cancer therapy (Fig. 9D) [232]. These designed targeted nanocomposites with excellent drug loading efficacy and colloidal stability presented substantial targeting efficiency to A549 tumor-bearing mice compared to pure drugs.

In most instances, the targeting ligand-immobilized polymeric shells coated over the MSNs have been predominantly developed to address the targeting of cancer and improved cellular internalization for intracellular delivery of therapeutic cargo [320]. However, the intracellular fate of these delivered MSNs with the aid of targeting ligands has become another issue to be addressed [102, 103, 320]. In this vein, to further explore the mechanistic views of therapeutics and their fate along with the interactions with the subcellular organelles, several ligands that recognize the intracellular organelles have been designed or discovered to target various organelles such as TAT peptide, triphenylphosphonium, and coumarin, among others [171, 320–322]. Predominantly, the nucleus and mitochondria are the specific organelles that would substantially facilitate the room for cancer therapy due to their participation in many cellular growth events. However, it should be noted that several features must be considered while fabricating the delivery systems, in which the eventual particle size of the MSNs must not exceed over 100 nm, as the passage of the large-sized constructs is challenging intracellularly. More often, the cellular and organelle targeting are two different aspects with altered surface features, which resulted in multiple camouflaged layers over the MSNs with multi-step synthesis procedures [323]. Considering these aspects, it is required to develop the common targeting ligands for cell membrane and specific organelles to deliver therapeutic guests more effectively.

#### Cancer immune therapy

**Fig. 10:**
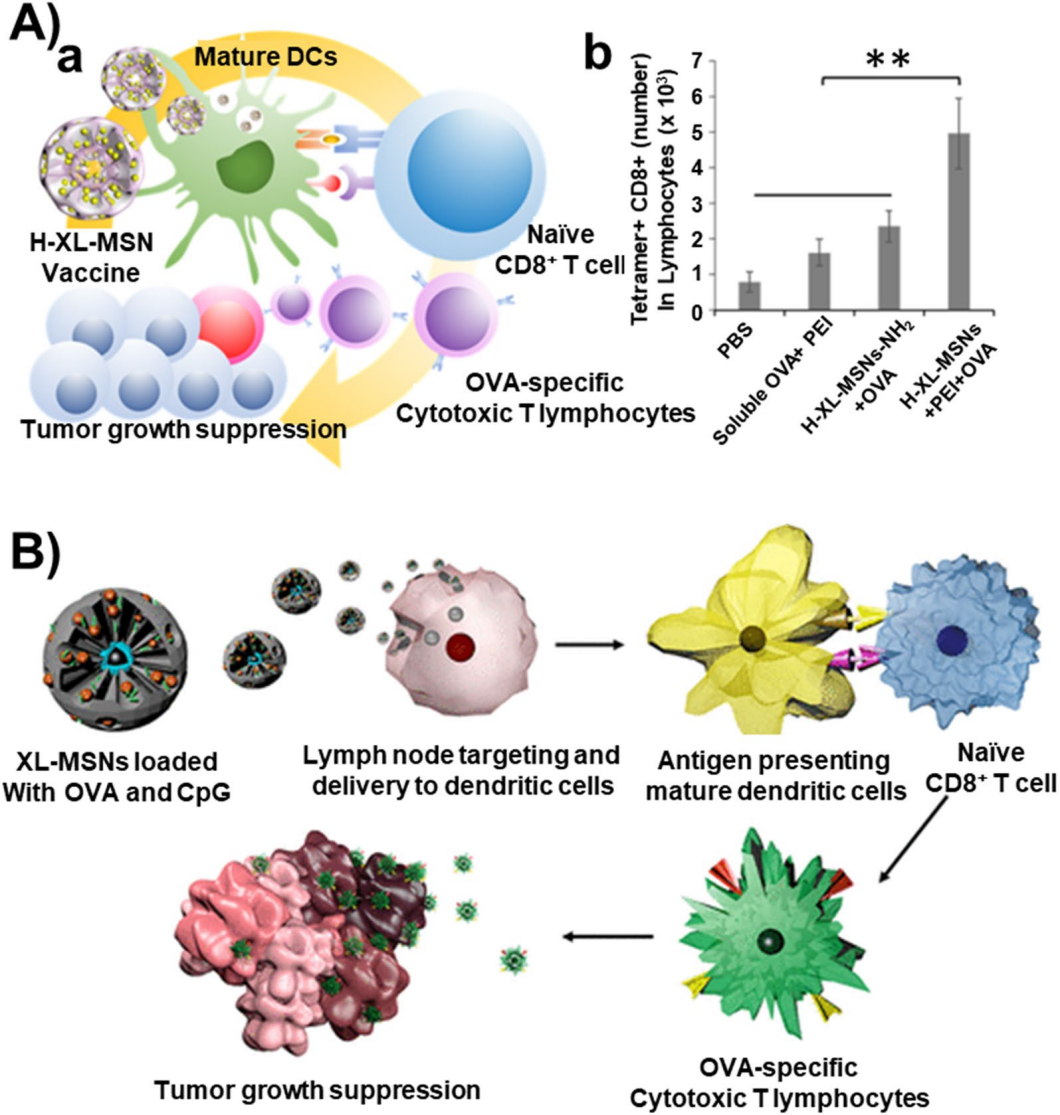
**A**, **a** Schematic of the immunological sequence of H-XL-MSN cancer vaccine to suppress tumor growth. **b** Level of SIINFEKL-specific CTLs in the lymph node. Seven days after vaccination (n = 4) (error bars, mean ± SD; **P < 0.0005). Reproduced with permission from Ref. [233] Copyright 2020, American Chemical Society. **B** Schematic illustration of the overall vaccination process using extra-large pore MSNs (XL-MSNs) loaded with antigen and TLR9 agonist to invoke antigen-specific cytotoxic T lymphocytes (CTLs) to suppress tumor growth. Reproduced with permission from Ref. [234] Copyright 2018, American Chemical Society. Further permissions related to this material excerpted should be directed to the ACS

Despite the success in utilizing numerous non-targeted and targeted delivery systems, the conventional cancer therapy based on various delivery systems for conveying small molecular drugs and genes offer limitations, such as adverse effects due to overdose, complex surgical strategies, and cancer recurrence [233]. To address these challenges, cancer immune therapy has emerged as the potential therapeutic approach, which is increasingly recognized for preventing cancer recurrence due to cancer specificity and immune memory [234]. Notably, the immunotherapy acts by provoking the adaptive immune antigen-specific responses through several subsequent steps of draining to lymph nodes, internalization by dendritic cells and their activation and maturation, as well as presenting the MHC-1 complexes to CD8 + cells [324]. In a case, Lee et al. employed the extra-large porous MSNs (pore size of 20–30 nm), similar to flower-like constructs for loading the cancer vaccine to activate the dendritic cells (Fig. 10A) [233]. The extra-large porous constructs enabled the high loading of proteins, and the surface modification of these MSNs with PEI facilitated the slow release of the encapsulated antigen. Further, in-vitro and in-vivo experiments demonstrated that the cancer vaccine enhanced the antigen-specific cytotoxic cells and subsequent suppression of tumor growth. In another instance, the similar research group aimed at invoking the adaptive immune responses for tumor elimination using the co-delivering of antigen protein and toll-like receptors 9 agonist (Fig. 10B) [234]. Together, these innovative composites of advanced MSN prototypes presented excellent antigen-carrying capacity towards cancer immunotherapy. Although the encapsulation of antigens is favorable in MSNs, the influence of various physicochemical properties remained unclear, which would certainly influence their encapsulation and delivery efficacies, leading to the altered activation and maturation in the in-vivo cascade. To explore these aspects, Hong et al. synthesized the MSNs with an average size of 80 nm and different pore sizes (7.8, 10.3, and 12.9 nm) that were encapsulated with ovalbumin antigen [324]. The experimental results showed that the influence of pore size played a major role in the cross-presentation efficacy in the last step of the in-vivo cascade, which in turn presented that the lymph-node targeted constructs with B16F10 tumor antigens yielded excellent anti-tumor effects. Although this study explored the effect of pore size on the release of encapsulated antigens and subsequent activation of immune responses in-vivo, several other physicochemical properties, for instance, surface functionalities, yet remained to be explored. Further, the targeted delivery of the antigens are yet required to be demonstrated towards efficacious anticancer therapy.

#### Other ailments

In addition to enormous research on cancer therapy using the conventional and advanced stimuli-responsive delivery patterns, MSNs and their advanced prototypes have been utilized to deliver various therapeutics to other organs for better health, for instance, heart, brain, and others [325, 326]. In this section, we discuss various aspects of delivery systems using the MSN-based nanosystems for innovative therapeutics, highlighting their ability to convey the therapeutics overcoming the complex physiological barriers. In a case, a combination of therapies based on advanced MSN prototypes was employed to treat myocardial infection by RNA delivery for anti-inflammation and anti-angiogenesis [326]. A minimally-invasive, pH-responsive hydrogel matrix was encapsulated with the MSNs for the on-demand controlled release of microRNA-21-5p in the acidic microenvironment, which substantially acted by reducing the inflammatory responses through inhibiting the polarization of M1 macrophages and conquering the TLR/NFκB signaling pathway. Moreover, the additional intrinsic effects of localized delivery of microRNA by MSNs on the further delivery to endothelial cells improved the local neovascularization by targeting SPRY1 and triggering VEGF-induced ERK-MAPK signaling. The subsequent stages of stimuli-responsive release of RNA from the MSNs-based hydrogel matrix addressed inflammation in a couple of stages and neovascularization, leading to the protection of affected cardiomyocytes. Despite the successful exploration of in-vitro and in-vivo (porcine model) demonstrations and their targeted effects, as well as mechanistic views, the injectable administration of the hydrogel matrix lacks the precise targeting efficacy, which could be the reason for hampering their translation to clinics.

In addition, these advanced MSNs have been applied to deliver various therapeutics towards treating neurodegenerative disorders, such as Alzheimer’s and Parkinson’s diseases [327, 328]. Despite the ability to deliver the drugs (for instance, Dopamine, _L_-Dopa) by MSNs, the complex blood–brain barrier would undoubtedly limit the overpass of MSNs. In several instances, the uptake and transportation through the complex blood–brain barrier of advanced nanocomposites, such as polymer-coated MSNs, were demonstrated in various in-vitro models (rat brain endothelial cells, RBE4). The surface-functionalized MSNs presented an enhanced uptake efficacy compared to conventional MSNs [327]. Despite the success, it is required to demonstrate in-vivo elucidations of these nanocomposites as the different and complex anatomical features in-vivo always result in altered pharmacokinetic behaviors compared to in-vitro models. Similarly, AuNPs-encapsulated MSNs were employed to demonstrate the metal ions induced reduction in the amyloid-β aggregation towards the H_2_O_2_-responsive controlled release for Alzheimer’s treatment [139]. Moreover, these nanocomposites were supported to overcome the adverse effects of the metal chelators when administered alone, which were evident from the clinical studies [139]. Recently, Morales et al. utilized the drug-structure directing template to fabricate the L-Dopa-decanoyl chloride and synthesized anionic complex structures through amidation and subsequent MSNs for Parkinson's disease [80]. These strategies enclosing the therapeutic agents and the templates presented the reduced release in the gastric pH compared to conventional MSNs. In contrast, the release was improved in the intestinal pH in a sustained fashion to relieve the symptoms of Parkinson's disease. Although the release was controlled well when administered through the oral route in treating the neurodegenerative disorder, considering the complexity of the blood–brain barrier, it is too early to confirm the delivery accomplishments, requiring several other investigations in-vitro and in-vivo monitoring various parameters exploring PK-PD attributes. In an attempt to address the targeting ability of the delivery system to the brain, Song et al. fabricated lactoferrin molecules-conjugated silica nanoparticles, in which these iron-binding cationic glycoprotein molecules could effectively target the receptors overexpressed in the vascular endothelial cells of the blood–brain barrier [329]. The biocompatible carriers were modified with PEG to avoid protein adsorption and demonstrated the transportation efficiency in-vitro in three different cell lines of endocytes, pericytes, and astrocytes. The surface-immobilized targeting ligands substantially facilitated the crossing of the complex barrier through the receptor-mediated transcytosis pathway. Notably, these ligands were more efficient than the transferring molecules, which would be considered an excellent choice for delivering the theranostics to the brain through the blood–brain barrier. Recently, polysorbate 80-immobilized, calcium-doped MSNs were employed to deliver rivastigmine to brain [330]. These nanosystems with excellent compatibility in blood and brain cells presented improved pharmacokinetics, exhibiting better brain uptake clearance of the designed nanocomposites compared to free drug. In addition, the performance attributes concerning the therapeutic efficacy and mechanistic views of the delivered drugs must be explored comprehensively. Further, the elimination kinetics of the traversed advanced prototypes should also be considered while developing these advanced prototypes.

Diabetes is one of the major health problems globally affecting millions, in which one in five adults is diagnosed with this serious metabolic disorder. In general, several treatment strategies have been employed based on the type of diabetes, for instance, direct administration of insulin for type-1 diabetes and delivery of various therapeutics promoting the insulin release from the β-cells of the pancreas for type-2 diabetes. Since ever the pancreatic insulin was purified in 1922 by Leonard Thompson, the insulin-based formulations have been well established for type-1 diabetes. However, the direct administration of insulin often poses various limitations of multiple self-administration doses, invasiveness, and irregularities, causing serious complications, such as dizziness, unconsciousness, and even death [331]. In this vein, insulin was primarily delivered by applying conventional MSNs, presenting their release in sustained and controlled fashions, owing to their extensive carrying ability and compatibility [331]. Although the encapsulation and carrying abilities are exceptional for delivering the insulin by MSNs, the appropriate time and position to release insulin remained as query for employing these delivery systems. Further advancements have resulted in the fabrication of glucose-responsive insulin delivery. In a case, Xu et al. demonstrated the fabrication of a glucose-responsive insulin delivery system using the hollow MSNs, which were further coated with the enzyme-polymer layer-by-layer composite system [332]. Interestingly, polymer-coated MSNs presented the insulin release threshold at a varied range for the first time in response to glycemia. The bioefficacy measurements were demonstrated in type-1 diabetic mice, in which the glycemic levels were regulated up to 84 h. In another case, boronic acid-functionalized MSNs were designed for glucose-responsive controlled delivery of insulin and cyclic adenosine monophosphate (cAMP), in which the insulin-FITC conjugates acted as gatekeepers, facilitating the balanced release of encapsulated cAMP in different pH conditions [333]. In addition, the mSiO_2_ particles were coated with glutaraldehyde-linked enzymes, enabling the release of insulin from their mesopores in response to external glucose levels [334]. Although these investigations proposed extensive insulin delivery, the appropriate release of insulin in the bloodstream and the insulin release, as well as long-acting efficiencies yet remained explicitly explored. Moreover, the extended-release efficacy and safety of degraded silica remained comprehensively investigated.

In addition to insulin delivery, several sensitizers have been delivered using MSNs to promote the secretion of insulin release from the pancreas. In a case, our group has demonstrated the encapsulation of a natural dipeptidyl peptidase-4 inhibitor in MSNs, i.e., 16-Hydroxycleroda-3,13-Dine-16,15-Olide, for potential hypoglycemia in diabetic mice [325]. Although the encapsulated natural molecule explored the GLP-1 degradation and reduced the blood glucose levels, the optimal parameters and application of large porous molecules for the encapsulation of such large-sized natural molecule must be further explored. Recently, poly(3-acrylamidophenylboronic acid) (PAPBA)-coated HMSNs-encapsulated were integrated into the transcutaneous microneedle patches to deliver metformin [335]. The polymer graft over HMSNs prevented the drug release in the normoglycemic levels, while the rapid glucose-responsive drug release was evidenced in the hyperglycemic levels due to a change in the hydrophilicity of the PAPBA at a high concentration of glucose. The transdermal administration in rats presented excellent hypoglycemic effects over the subcutaneous injection. However, challenges such as comprehensive safety attributes, stability of the needles after administering, and extended-release for prescribed time intervals remain to be explored.

**Fig. 11:**
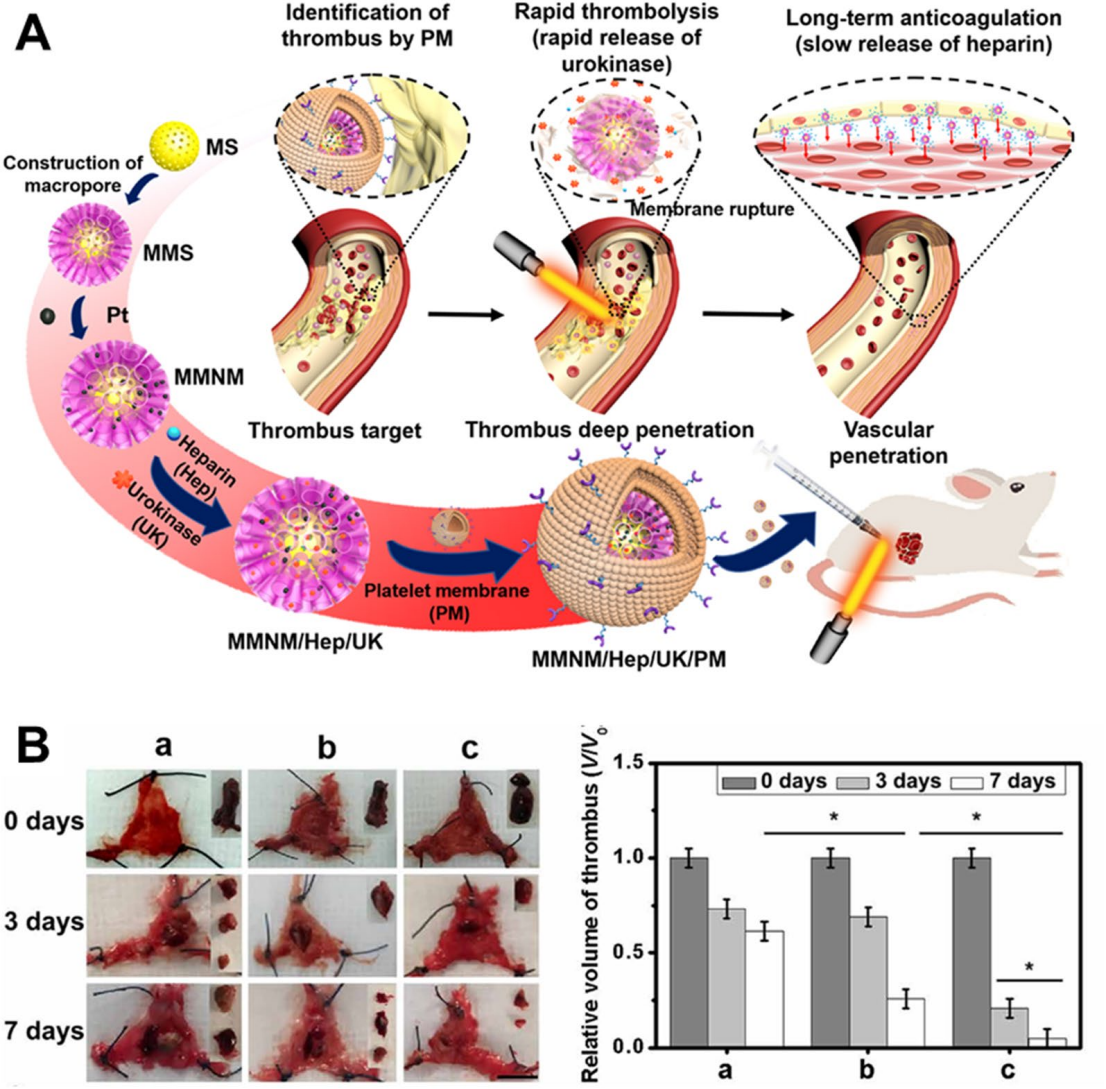
**A** Schematic illustration of the fabrication of MMNM/Hep/UK/PM nanomotors and their application for thrombus targeting and thrombolytic treatment. **B** Photographs of blood vessels and thrombus at 0, 3, and 7 days of different samples and corresponding relative volume of thrombus (**a**, blank; **b**, UK; **c**, MMNM/Hep/UK/PM with NIR irradiation; scale bar, 0.5 cm). Reproduced with permission from Ref. [337] Copyright 2020, American Association for the Advancement of Science

Although the bloodstream in the body is highly accessible, several ailments often lack accessibility for treatment, similar to various major organs, such as the brain. Among such ailments, venous thrombosis has been facing several treatment challenges, such as poor penetration ability and short half-life of the therapeutics [336]. To address these challenges, Wan et al. explored the fabrication of innovative nanomotors (flower-like) based on platelet-modified mesoporous/macroporous silica/platinum nanomotors to delivery of thrombolytics (urokinase) and anticoagulants (heparin) for thrombus treatment (Fig. 11A) [337]. The proteins in the decorated platelet modification regulated the targeting ability, and its subsequent NIR-assisted degradation enhanced the release of the encapsulated urokinase rapids and heparin sustainably in 3 h and 20 days, respectively. Moreover, the interesting motion ability of the nanomotors could effectively enhance the thrombolysis effect in the rat models with static and dynamic thrombus (Fig. 11B). Although the targeting ability and their stimuli-responsive controlled release abilities for effective thrombolysis, the optimization of the asymmetric Pt encapsulation is still required to be demonstrated for exploring the effective motion abilities.

### Bioimaging

Indeed, one of the exciting attributes of nanomedicine is the use of nanoparticles as contrast agents, which support the visualization of the functional and anatomical features in delineating diseased sites from the healthy tissues. This imaging-based approach substantially allows the clinicians to recommend appropriate treatment options [338]. In this context, most of the nanoparticles are often employed for imaging as some of them lack tracking capacity but carrying ability of various fluorescent species [4, 5, 338]. To this end, various inorganic species with intrinsic optical and magnetic properties have been employed for bioimaging applications. Notably, compared to the organic-based fluorescent molecules that suffer from aggregation-caused quenching, the nanodevices, such as organics-encapsulated in nanoparticles and inorganic nanoparticles-based contrast agents, potentially offer higher contrast efficacy. To overcome the limitations of various organic- and inorganic-based materials, various nanoengineering strategies have been employed to encapsulate various contrast agents for multiple diagnostic modalities, such as fluorescence imaging, MRI, computed tomography (CT), positron emission tomography (PET), single-photon emission computed tomography (SPECT), and photoacoustic imaging (PAI) [339–343]. However, critical advancements in material development to enable the localization and potentially high efficiency of contrast agents play crucial roles in facilitating the significant visualization of biological tissues for all the mentioned approaches.

MSNs have been utilized as versatile nanoplatforms by encapsulating various contrast agents for subsequent diagnostic and imaging modalities due to their unique mesostructured attributes, such as high specific surface area, tunable pore volumes, flexible surface decoration features, and intrinsic biocompatibility. Moreover, these attributes of MSNs can offer substantial advantages towards addressing the aforementioned limitations of contrast agents. The contrast agents-integrated MSNs improve the diagnostic performance of the imaging probes, leading to early detection, substantial real-time therapeutic monitoring through drug release profiling, and subsequent validation in providing pathophysiological changes for effective therapeutics. This section provides insights into notified bioimaging techniques, specifically, MRI, optical/fluorescence, PET, and other multi-modal imaging modalities, considering the importance of the imaging modality and amount of published literature based on advanced prototypes MSNs (as listed in Table 2).

**Table 2:** Various examples of advanced prototypes of MSNs for bioimaging

Mode	Imaging modalities	Advanced composites	Particle size (nm)	Pore size (nm)	Contrast agent	Animal model	Refs
Single-mode	T1-MRI	MnOx-HMSNs	200	4.4	MnOx	Rats	[459]
	MRI	fmSiO_4_@SPIONs	50	2.8	SPIONs	Mice	[345]
	T1 MRI	mSiO_2_@GGO NPLNPs	50	–	Cr^3+^, Nd^3+^, Gd_3_Ga_5_O_12_	Kunming mice, BALB/c mice	[347]
	T1/T2 MRI	MnFe_2_O_4_@ mSiO_2_	50	2.4	MnFe_2_O_4_	Kunming mice	[344]
	T1/T2 MRI	Fe_3_O_4_@MnO/mSiO_2_	45	2.5	Fe_3_O_4_@MnO	–	[460]
Dual-mode	T1/T2 MRI, FL	Fe_3_O_4_@mSiO_2_	45–105	2–3	FITC, RITC	Balb/c mice	[15]
	T2 MRI, FL	Fe_3_O_4_-MSN	70 ± 6	2.3	FITC, RITC	Balb/c mice	[461]
	PET, CT	^89^Zr-labelled MSNs	180	8	^89^Zr	SCID mice	[366]
	FL, T1 MRI	MSN@QDs	101.2	3.9	Mn-doped ZnSe QDs	Balb/c mice	[182]
	UCL, CT	UCNPs@mSiO_2_-POM @FC	120	–	Yb	Kunming mice	[462]
	FL, PAI	17AAG@HMONs- Gem-PEG	60	–	ICG	Balb/c mice	[463]
	TPF, T2 MRI	Fe_3_O_4_@CDs@mSiO_2_ @PTX@mSiO_2_	91	2.9	Fe_3_O_4_, CDs	Balb/c mice	[464]
	T2 MRI, PL	CoFe_2_O_4_@mSiO_2_	52	8	-	–	[465]
	T1 MRI, FL	MS‐Gd_2_O_3_:Eu@PEG	190 in length, 70 in width	~ 4	Gd_2_O_3_	–	[211]
	T2 MRI, FL	Fe_3_O_4_@mSiO_2_ -CD-FA	100–150	4.72	CD, Fe_3_O_4_	–	[466]
	T1 MRI, PL	GdVO_4_:Eu^3+^@mSiO_2_	75	3.3	GdVO_4_, Eu^3+^	–	[361]
	^19^F MRI, FL	Au-FMSNs	~ 100	< 1.2	Au, C_6_F_6_, FITC	–	[467]
	T2 MRI, FL	MSN-Gd	> 75	1.4–2.9	Gd^3+^	–	[468]
	UCL, PAI	UCNP@mSiO_2_-ICG	~ 60	–	Nd, Er, ICG	ND4 Swiss Webster mice	[270]
	T2 MRI, CT	M-MSN(Dox/Ce6)/ PEM/P-gp shRNA	280 ± 17	2.482	Fe_3_O_4_-Au	Balb/c mice	[264]
	T1 MRI, UCL	NaYF4: Yb/Er@NaGdF4@ SiO_2_@mSiO_2_	240–265	2.23, 4.01	Gd	Balb/c mice	[469]
	T1 MRI, FL	Gd-Al@MSNs	57	2.5	Gd-Al, Cy5	Balb/c mice, Kunming mice	[470]
	UCL, CT	Y_2_O_3_:Yb, Er@mSiO_2_	340	4.6	Y_2_O_3_:Yb, Er	Balb/c mice	[471]
	FL, CT	A-AuNC@PAA/mSiO_2_	120	2	Au	Balb/c mice	[472]
	MRI, UCL	β‐NaYF_4_:Yb^3+^, Er^3+^ @β‐NaGdF_4_:Yb^3+^@mSiO_2_	78	2.9	β‐NaYF_4_: Yb^3+^, Er^3+^ @β‐NaGdF_4_: Yb^3+^	Kunming mice	[473]
	T1 MRI, UCL	MUCNCs@SNTs	95 in wall thickness	5	NaYF4:Yb/ Er/Gd	–	[474]
	T2 MRI, FL	Hydrazine-MSN- FITC-Fe_3_O_4_-PEG	–	–	Fe_3_O_4_, FITC	–	[353]
Multi-mode	FL, MRI, CT	WS_2_-IO@MS-PEG	–	2.48	WS_2_, Iron oxide	Balb/c mice	[141]
	PAI, PET, FL	CuSNDs@DOX-MSNs	100	6	CuSNDs	Nude mice	[475]
	US, CT, PAI, Thermal	HMSs@Au-PFH-mPEG NSs	200	3.8	Au	Balb/c mice	[374]
	T2 MRI, FL Thermal	Au-NRs-MMSNEs	300 × 180	2–3.5	Au, Fe_3_O_4_	SD mice	[142]
	PT, FL, T1 MRI	RGD-CCmMC	116.5	2.9 ± 0.3	Mn-Cdots, gold cube-in-cube	Balb/c mice	[476]
	T2 MRI, US, FL	mSiO_2_-MNPs	9.4 ± 1.2	2, 4	Fe_3_O_4_, FITC	–	[370]
	TPL, TPEF, FL, PAI, PT	GNR@mSiO_2_-5-FU	54.14 ± 4.39 in length and 15.87 ± 1.28 in width, a shell of 19.85 ± 1.67		GNRs, ICG	Balb/c mice	[477]
	SR‐STXM, CT, T1 MRI, PA	Au@SiO_2_(Gd)@HA	232.7	–	Gd	Balb/c mice	[346]
	T2 MRI, PAI, BL	GRMNBs	130	2.1	Au, Fe_3_O_4_	C57BL/6 mice	[371]
	PAI, MRI, FL	Au@SiO_2_	200	4.6	AuQDs	CD1 mice	[478]
	UCL, CT, PT, T1/T2 MRI	GdOF: Ln@SiO_2_– ZnPc-CDs-FA	293	3.53	GdOF, ZnPc, CDs	Balb/c mice	[479]
	T1 MR, CT, UCL	UCMSNs	-	3.1	Yb3^+^, Gd^3+^	Balb/c mice	[273]
	T1 MRI, PET, FL	MSN@Gd@^64^Cu	~ 60	-	Gd^3+^, ^64^Cu, FITC	Balb/c mice	[358]
	T1 MRI, FL, US	MSN-Gd	384 ± 134 nm	4.1 ± 1.1 nm	GdCl_3_, Fluorescein	nude mice	[480]
	US, CEUS, T2 MRI	Fe-HSNs	200.3 ± 15.2	6.03	-	C57BL mice, ApoE-/- mice	[368]
	T2 MRI, CT, FL	i-fmSiO_4_@SPIONs	50	2.7	Fe_3_O_4_	–	[373]

17-AAG: 17-*N*-allylamino-17-demethoxygeldanamycin or Tanespimycin; 5-FU: 5-Fluorouracil; A-AuNC: Aggregated gold nanocluster; Al: Aluminum; Au: Gold; BL: Bioluminescent; CDs: Carbon dots; CEUS: Contrast-enhanced ultrasound; Ce6: Chlorin e6; CPT: Camptothecin; CT: Computed tomography; Cu: Copper; Cy 5: Cyanine 5; DOX: Doxorubicin; FA: Folic acid; FITC: Fluorescein isothiocynate; FC: Folate-chitosan; FL: Fluorescence; FMSNs: Fluorescein-functionalized MSNs; Gd: Gadolinium; Gem: Gemcitabine; GNR@mSiO_2_: Mesoporous silica‐coated gold nanorods; GRMNBs: Multi‐gold nanorods crystal‐seeded magnetic mesoporous silica nanobeads; HA: Hyaluronic acid; HMONs: Hollow mesoporous organosilica nanocapsule; HMS -Hollow mesoporous silica; HSN: Hollow silica nanoparticles; i-fmSiO_4_@SPIONs: Iodinated oil-loaded mesoporous silica-coated superparamagnetic iron oxide nanoparticles; ICG: Indocyanine green; mC: Magnetic carbon; MMSNEs- Magnetic mesoporous silica nanoellipsoids; Mn: Manganese; MNPs: Mesoporous silica-coated magnetic nanoparticles; mPEG: Methoxypoly(ethylene glycol); MRI: Magnetic resonance imaging; mSiO_2_ or MS: Mesoporous silica; MSNs: mesoporous silica nanoparticles; MUCNCs@SNTs: Silica nanotubes functionalized with NaYF4:Yb/Er/Gd nanocrystals; NPLNPs: Near-infrared persistent luminescence nanoparticles; NRs: Nanorods; NSs: Nanostars; P-gp: P-glycoprotein; PAA: Polyacrylic acid; PAI: Photo acoustic imaging; PEG: Poly(ethylene glycol); PEM: Alginate/chitosan-based polyelectrolyte multilayers; PET: Positron emission tomography; PFH: Perfluorohexane; PL: Photoluminescence; POM: Polyoxometalate; PT: Photothermal; PTX: Paclitaxel; QDs: Quantum dots; RITC: Rhodamine isothiocyanate; ShRNA: small hairpin ribose nucleic acid; SR-STXM: Synchrotron radiation scanning transmission X‐ray microscopy; TA: Trimethylammonium groups; TPEF: Two‐photon excitation fluorescence; TPF: Two‐photon fluorescence; TPL: Two‐photon luminescence; TRF: Transferrin; UCNPs: Upconversion nanoparticles; UCL: Upconverting luminescence; UMSCs: Umbilical cord‐derived mesenchymal stem cells; UCMSNs: NaGdF4:Yb,Er@NaGdF4:Yb@NaNdF4@mSiO_2_-CuS-PEG; US: Ultrasound; Yb^3+^: ytterbium trivalent ion

#### MRI

Various advanced metals/contrast agents-encapsulated MSNs have been employed for more precise and effective MRI-based diagnostic purposes, such as gadolinium complexes and iron oxide composites [344–347]. In general, these contrast agents act by depending on the proton relaxation stimulated by an externally-applied magnetic field, resulting in the production of endogenous contrast ability in the time course [338, 343]. Moreover, this imaging modality is one of the most used approaches clinically due to the superior 3D tissue contrast efficiency, high stability, and high spatial resolution [338]. Despite the various advantages over other imaging techniques, MRI suffers from a significant drawback of limited sensitivities of its contrast agents. Although gadolinium complexes as the exogenous contrast agents for clinical MRI have shown excellent resolution and sensitivity, in-depth investigations on the improvement of relaxivity and diagnostic performances are required to detect pathophysiological conditions of diseases. Moreover, the FDA has cautioned that the Gd complexes could cause severe complications of nephrogenic systemic fibrosis (NSF) in patients, leading to the search for effective alternatives to these composites. Recently, manganese-based MRI agents have been found for utilizing them as an alternative to Gd complexes as these species contain five unpaired electrons, variable valence states with strong oxidizing ability, and long electronic relaxation time [348]. In a case, MnO_2_ was doped into the porous networks of MCM-48, enabling the labeling of cells and tracking these nanocomposites [349]. These nanocomposites with excellent colloidal stability offered high longitudinal relaxivity values and highly positive contrast properties at a low concentration, facilitating them as effective cell tracking composites. In another case, manganese oxide-MSNs were fabricated for targeting prostate-specific membrane antigen, which subsequently offered excellent colloidal stability, targeting efficacy, and good T_1_ relaxivity [314]. Although the designed composites offered excellent contrast efficiency and excellent relaxivities, in some instances, the compatibility issues might have significantly hampered the utilization of these MSNs due to the accumulation-induced risk of silica and MnO_2_. To overcome these aspects, Yu et al. fabricated Mn-doped MSNs in which the EPR-assisted accumulation of these nanocomposites offered excellent T_1_-weighted MRI [350]. Interestingly, the doped Mn species were released specifically in tumor environment precisely, referred to as Mn-extraction, resulting in the selective degradation of the Mn-doped composites in the tumor. However, further optimization of Mn doping amount and investigations related to improved metabolism, distribution, specificity, and targeting efficacy are required.

On the other hand, highly crystalline superparamagnetic iron oxide nanoconstructs are used as highly efficient T_2_-weighted MRI platforms [351]. The iron oxide composites based on the thermal decomposition synthesis approach often possess highly crystalline structures with hydrophobic surfaces, which limit their applicability in-vivo. To a considerable extent, these limitations can be addressed by coating the superparamagnetic constructs with the hydrophilic mesoporous silica shell [71]. Due to the abundant surface chemistry and the eventual constructs can be further immobilized with the high Mol. Wt. polymers and targeting moieties augment their circulation time in the physiological fluids and targeted diagnosis [351, 352]. In a case, Kim et al. fabricated mesoporous silica-coated iron oxide composites, in which the silica coating significantly improved the relaxivities of the iron oxide composites in the physiological fluids [15]. Further, PEGylation of these silica-coated iron oxide composites enhanced their accumulation in the tumor tissues of mice after intravenous administration via the EPR effect, which was confirmed by the signal enhancement in the T_2_-weighted MRI. In addition, these accumulated particles were retained for more than 24 h, indicating their suitability for long-term bioimaging application [353]. Moreover, chelation of Gd^3+^ into the mesopores of the iron oxide-coated mSiO_2_ core–shell composites could further enhance the transverse relaxivity (r_2_), demonstrating that the combinatorial efficacy of both these contrast agents could yield in the effective MRI in-vivo.[47, 351, 354] Interestingly, the mesoporous silica shell coating over these contrast agents offers enormous advantages toward highly efficient MRI. In particular, the porous shell with unique morphological attributes of well-ordered structural features provides accessibility of water molecules to the paramagnetic centers, allowing their anisotropic diffusion to improve the imaging efficiency [9, 348]. In addition to improved imaging efficacy, these iron-oxide-based constructs require no auxiliary targeting ligands as these could be managed by externally applied magnetic field-guided targeting to various cancers in-vivo. Notably, this physical targeting approach offers more advantages over the active and passive targeting approaches in terms of controlled “ON–OFF” strategy and no requirement of multi-functionalization steps. Moreover, the silica thickness could be optimized for controlled and improved MRI contrast efficiency. Despite the compatibility to a certain extent, it is a long way to go for the FDA approval to further clinical translation of these iron-oxide-based MSN nanocomposites.

#### Optical imaging

Optical imaging is another innovative modality used for bioimaging with comparatively offering advantageous features over MRI, such as cost-effective, high sensitivity, a relatively wide selection of fluorescent probes, and biosafety, among others [355]. Various types of fluorescent moieties can be used to execute optical imaging, including fluorescent organic molecules and inorganic nanoparticles, such as quantum dots and rare-earth up-conversion constructs for an imaging application. To this end, the classic NIR fluorescence agent, ICG, has been approved for clinical application of surgical resection, owing to low toxicity [270, 355]. As mentioned earlier, these organic-based fluorescent molecules suffer from specific drawbacks of aggregation-caused quenching and photo-bleaching effect. Even the classic ICG is no exception, as it suffers from deprived solubility in the physiological environment and poor fluorescence quantum yields in the aqueous environment [356]. To a considerable extent, these fluorescence molecules integrated with the carriers like MSNs can overcome such limitations due to their unique structural attributes. Moreover, these siliceous matrices offer optical transparency, simultaneous optical imaging, and therapeutic functionalities by transporting therapeutic guests, such as fluorescein and ZW800 [17, 253, 357]. Moreover, these innovative strategies result in not only the dynamic optical imaging of tumors but also help in demonstrating the biodistribution behavior of the nanocarriers [75, 358]. In a case, ICG and sorafenib were encapsulated in MSNs to improve real-time fluorescence imaging with enhanced cellular internalization and longer red fluorescence signal retention [359]. To further improve the fluorescence imaging, the polymer-grafted MSNs enclosed with the fluorescent molecules have been fabricated to cap the mesopores with the polyelectrolytes [360]. Moreover, core–shell nanocomposites encapsulated with fluorescent nanocrystals have been explored for molecular imaging, which resulted in simultaneous enhancement of stability and performance efficiency of the fluorescent probes [117]. Other advantages of high quantum yield and resistance to photobleaching effects made them very promising for optical bioimaging. Moreover, other fluorogenic molecules have been used as alternatives to conventional fluorescent organic molecules with tunable fluorescent attributes, such as aggregation-induced emission (AIE) fluorogen (PhENH_2_) and up-conversion luminescent quantum dots [361, 362]. Compared to the conventionally immobilized fluorescent molecules, the conjugated AIE fluorogenic species present more stability. Although these are highly compatible and efficient bio-imagers, extensive investigations regarding the synthesis are required to explore the physicochemical attributes and their effects in-vivo for their use in the future.

#### Miscellaneous

Apart from these imaging modes based on MRI and fluorescent organic agents-based optical imaging, various imaging techniques have been utilized by encapsulating diverse contrast agents in the encapsulated MSNs such as PET, ultrasound imaging, PAI, and computed tomography [363–365]. In most instances, these imaging modalities are often favorable in imaging in-vivo toward the early diagnosis of various ailments. In this framework, several advancements have been evidenced in fabricating various contrast agents-encapsulated formulations towards improving their intrinsic imaging amplitude [270, 366]. PET imaging has garnered enormous interest toward the early diagnosis of tumors in-vivo due to its high sensitivity, unlimited penetration depth, non-invasive, and the broad range of probe selectivity [363]. Moreover, it should be noted that this imaging modality is the only currently employed approach for metabolic and functional imaging of organs for any ailments. This imaging technique is often employed in nuclear medicine, in which the carriers, for instance, MSNs, are utilized to encapsulate radionuclides (^64^Cu) for improved imaging efficiency [358, 366, 367].

In addition, ultrasound-based imaging has been employed to screen various diseases due to several benefits of cost-effective, non-invasiveness, flexibility, real-time monitoring, and non-ionizing [276, 290]. The ultrasonography approach requires high efficient contrast agents due to its poor resolution efficiency and sensitivity compared to other imaging modes, such as MRI and PET [365]. In general, this imaging modality comprises microbubbles stabilized in polymeric or lipid shells to improve the imaging resolution [368, 369]. Despite using organic bubbles for contrast enhancement, the micron-sized bubbles with instability seem highly challenging to fulfill the required tasks of early detection of tumors and other diagnostic applications. In a way, Jin et al. fabricated superhydrophobic silica spheres with high surface area for improving their performance as ultrasound agents with strong contrast intensity for over 30 min [369]. In another instance, hollow silica spheres have been developed to overcome these limitations by exhibiting exceptional echogenic behavior with excellent stability [7]. In terms of cancer imaging for early diagnosis, this imaging modality is often dependent on the EPR effect after administering contrast agents. To overcome this aspect, Pilapong et al. fabricated a targeted ultrasound imaging agent based on EpCAM aptamer-conjugated MSN-iron oxide nanoparticles to visualize EpCAM-positive cells (HepG2) [370]. Moreover, these silica-based bubbles significantly reduced the toxicity attributes compared to the organic bubbles as these innovative architectures with surface functionalities are convenient to immobilize targeting ligands. Despite the success, most of the research is focused on improving the contrast efficacy, requiring further investigations to address the targeting efficacy, and in-vivo fate of these nanoformulations in terms of distribution, metabolism, and sensitivity.

PAI, an advanced non-ionizing imaging technique integrated with ultrasound and optical imaging. Notably, the PET approach offers deeper penetration efficacy than the optical method and higher contrast efficiency than ultrasound imaging. Similar to ultrasound imaging, the PAI efficacy has been improved further in exploring the diagnostic characteristics by utilization of the MSNs [270, 364, 371]. In addition to encapsulating typical optical agents (ICG, DOX), nanocomposite forms of MSNs with the 2D materials (MXene) significantly provided excellent optical and ultrasound imaging effects due to SPR effects along with the photothermal effects in hepatocellular carcinoma [372]. It should be noted that the formation of core–shell improved the theranostic effects of the nanocomposite forms, which, however, require stringent control over the optimum synthetic conditions to enrich silica shell thickness and pore engineering. In addition, the careful considerations relevant to the compatibility attributes are yet remained to be explored.

To this end, CT has gained enormous interest in cancer diagnosis due to various advantages of rapid and detailed image generation and cost-effectiveness. This modality is based on utilizing small iodinated molecules as contrast agents, which suffer from short-term imaging due to rapid excretion, hampering their applicability [373]. The applicability can be improved by employing materials with X-ray attenuation properties [343, 374]. In a case, gold nanorods-encapsulated MSNs were utilized for the early diagnosis of cancer and improved photothermal therapy [375]. In another case, to address the reduced radiation-absorption efficacy of conventional MSNs, Janus-type mesoporous silica-coated gold nanomaterials were fabricated as a versatile platform for cancer theranostics. These nanocomposites conjugated with folic acid improved the targeting of hepatocellular carcinoma, resulting in reduced systemic toxicity. Overall, the versatile Janus platform presented significant tumor inhibition and CT imaging-assisted HCC diagnosis [376]. Further advancements on fabricating core-mSiO_2_ shell materials are required to be explored, which, however, needs to address several optimization parameters and the effect of silica shell on the CT properties. These advanced nanocomposites would certainly offer great potential to address the existing limitations.

#### Multi-modal imaging

**Fig. 12:**
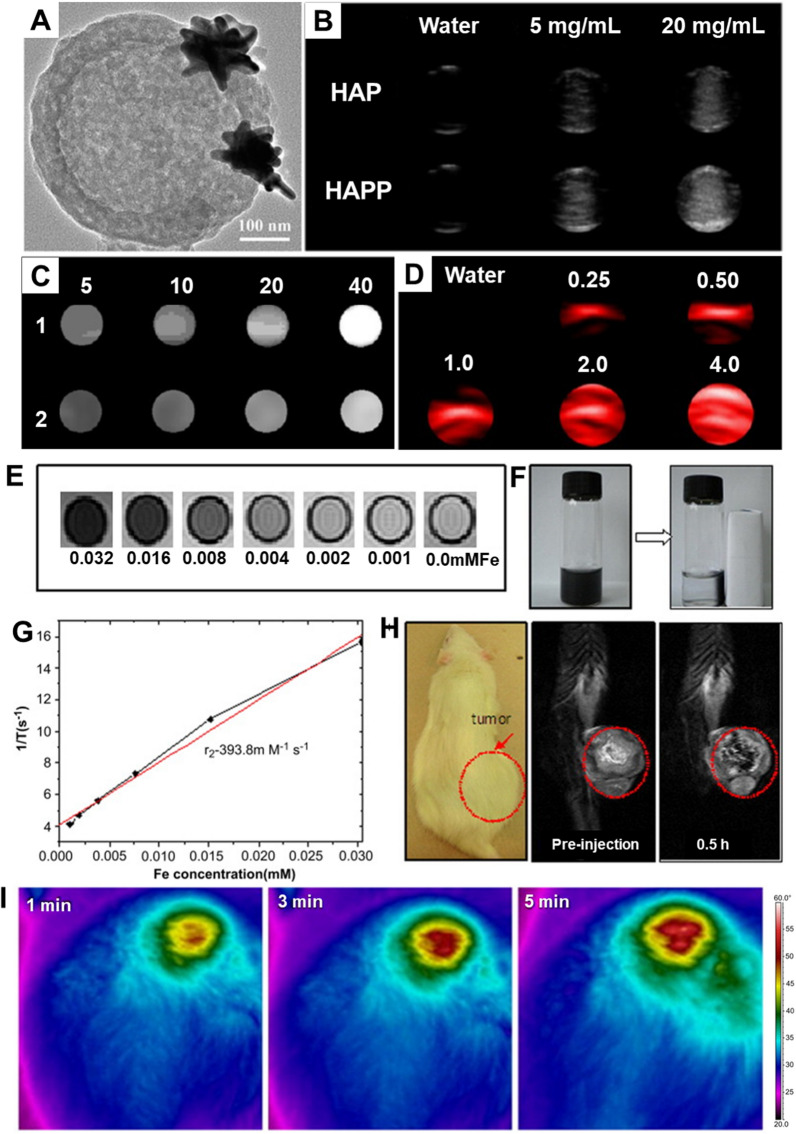
Advanced MSNs for bioimaging application. **A** High-resolution TEM images of HMSNs@Au nanostars. **B** Ultrasonographic images of water, HMSNs@Au nanostars@ perfluorohexane (HAP), and HAP@mPEG (HAPP) with different concentrations under contrast mode. **C** CT images of (1) HAPP and (2) Omnipaque with different concentrations of the radiodense element (Au or I). **D** PA images of HAPP with different Au concentrations. Reproduced with permission from Ref. [374] Copyright 2017, American Chemical Society. **E** T2 phantom images of AuNRs-MMSNEs at different Fe concentrations. **F** Relaxation rate 1/T2 of AuNRs-MMSNEs as a function of Fe concentration. **G** In-vivo MRI of a mouse before and after intratumor injection of Au NRs-MMSNEs. **H** Photographs of AuNRs-MMSNEs dispersed in water before (left) and after (right) an external magnetic attraction. **I** Infrared thermal imaging under the photothermal heating by 808 nm laser irradiation for different periods in AuNRs-MMSNEs-injected tumor under 2 W cm^−2^ irradiation. Reproduced with permission from Ref. [142] Copyright 2012, Elsevier

In addition, there has been growing interest in establishing the advanced type of multimode imaging as a theranostic platform using MSNs to provide high accuracy and high imaging efficiency. Moreover, these imaging modes possess pros and cons, critically requiring multimodal imaging to overcome these demerits and combine their merits to allow better therapeutic outcomes through accurate diagnosis. Such active imaging probes encapsulated within the highly porous MSNs would facilitate multimode imaging purposes (Table 2) [358]. Moreover, these mesostructured architectures offer the augmented stability and intracellular retention time of nanoprobes, enabling long-term imaging. In one case, Li et al. designed distinct, versatile nanostructures based on Au nanostars-coated and perfluorohexane-encapsulated hollow MSNs modified with mPEG-SH for multimodal ultrasound/CT/PA/thermal imaging and PTT (Fig. 12A–D) [374]. Initially, the fabricated HMSNs were modified with thiol groups for coating Au through Au–S linkages. Further, several steps were performed to fabricate an eventual versatile theranostic nanoplatform, such as the growth of Au nanostars on MSN surface, encapsulation of perfluorohexane in the mesopores, and the surfaces modified with the thiolated PEG. Owing to their accumulation specifically in the tumor site due to the EPR effect of cancer, the intravenously administered nanocomposites exhibited exceptional stability in the physiological environment and augmented the contrast efficiency, reaching the highest signal intensity at 2 h post-injection in the tumor region. In addition, these composites resulted in NIR-assisted photothermal conversion efficiency of around 67.1%. These experimental results suggested that the fabricated biocompatible composites can be a potential theranostic platform for multimodal imaging and PTT for cancer ablation. In another case, Mn-doped ZnSe QDs were incorporated in the mesopores for improved synergistic dual (FL and MRI) mode imaging modalities, which was demonstrated with superior and complementary performance over the single-mode imaging [182]. Oftentimes, these MSNs enriched the encapsulated composites with large stokes shift towards highly luminescent/paramagnetism Mn-doped ZnSe QDs assembly. Similarly, Ma et al. fabricated uniform AuNRs-gated magnetic core/mesoporous silica shell nanoellipsoids (AuNRs-MMSNEs) and integrated MR/thermal/optical imaging in one platform (Fig. 12E–I), and synergistic chemo- and photothermal therapies were demonstrated [142]. Interestingly, the resultant versatile nanocomposites exhibited high intracellular localization, apparent darkening of the tumor site in 0.5 h post intratumoral injection, and enhanced T_2_ relaxivity coefficient with increased concentration of Fe species, demonstrating that these nanocomposites could be used for T_2_-weighted MRI. In addition, the high loading efficiency of the chemotherapeutic agent and substantial release in the tumor environment due to the electrostatic interactions between the guest species and MSN host and augmented photothermal conversion efficiency of AuNRs indicated that the designed versatile composites could be used for synergistic therapy.

### Tissue engineering

Tissue engineering aims to repair malfunctioned tissues by fabricating 3D biomimetic constructs at arbitrary geometries while managing the physiological microenvironment and biochemical cues by emulating their native counterparts due to the shortage of donors for organ replacement therapy [377, 378]. In this context, numerous biodegradable constructs have been fabricated, such as porous 3D scaffolds, photo-crosslinkable hydrogels, and nano/microfibrous biocompatible materials. These innovative scaffolds mimic the natural extracellular matrix (ECM), facilitating the proliferation and differentiation of encapsulated cells, as well as providing efficient control over the physiological microenvironments for tissue growth [10, 378]. Despite the advances in tissue engineering, it still faces specific challenges concerning the macro and micro-sized tissue constructs, such as establishing coordination with the adjacent cells, the inability to mimic the complex biochemical environment, and insufficient migration capability. These critical shortcomings led the researchers to integrate the nanostructured components with the fabricated biodegradable constructs, providing enormous potential in improving functionalities and structural restoration of the biomimetic scaffolds [12]. The intrinsic molecular characteristics of the nanocomposites adjust the biobehavior of the macro-sized constructs by altering their spatiotemporal arrangement. These nanocomposites with the ability to convey certain guest species can also deliver therapeutic guest species (for instance, growth factors and small therapeutic molecules), which can synergistically augment tissue growth [29].

**Fig. 13:**
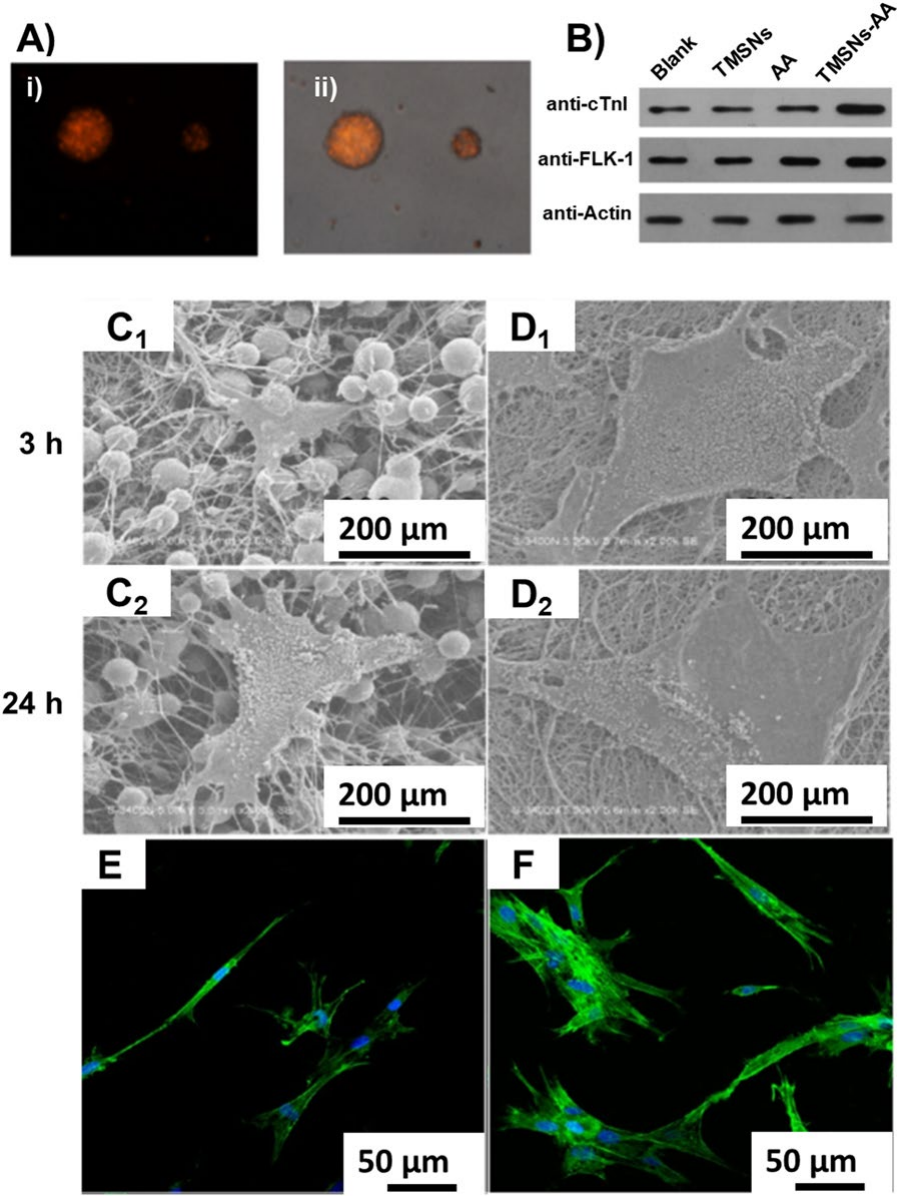
MSNs for tissue engineering and regenerative medicine. **A** Fluorescent images of human ESCs treated with tetramethylrhodamine (TRITC)-loaded MSNs (TMSNs) (i) TRITC and (ii) overlaid with a bright-field image of ESCs. **B** TMSN-AA nanoconjugates induced the differentiation of human ESCs toward cardiomyocytes (subjected to western blot analysis using antibodies against myocardial marker genes cTnI and FLK-1. Anti-actin was used as a loading control). Reproduced with permission from Ref. [380] Copyright 2015, Elsevier. **C–F** SEM images showing the attachment and spreading of hMSCs to the membranes after 3 and 24 h: (C_1_-C_2_) pure poly(lactide-co-glycolide) (PLGA); (D_1_-D_2_) PLGA-10% SBA-15. Cellular morphology and alignment of the hMSCs on different membranes: Cellular actin filaments stained with fluorescein isothiocyanate-phalloidin (green) and nuclei counterstained with 4′,6′-diamidino-2-phenylindole (blue) **E** pure PLGA; **F** PLGA-10% SBA-15. Reproduce with permission from Ref. [386] Copyright 2014, American Chemical Society

Indeed, MSNs are one of such innovative carriers recently applied for tissue engineering due to their intrinsic physicochemical features of colloidal stability, high dispersity, abundant surface chemistry, morphological attributes with high surface area, and tunable pores for encapsulating diverse guest species [74, 96, 379–381]. In addition, the surface hydroxyl groups of MSNs offering enormous hydrophilicity to the frameworks facilitate the enhanced interactions with the biological membranes and subsequent tissue growth [379]. Notably, silica has been recognized as one of the most biocompatible materials among various available inorganic constructs due to the several facts of existence as endogenous material in bone and commercial utility as an excipient in oral formulations [40]. More often, these innovative MSN-based nanoconstructs can be used for delivering protective drugs concerning the tissue engineering application, owing to their high porosity facilitating encapsulation efficiency of therapeutic guests for cellular engineering and stem cell therapy [382]. Numerous stem cells have been preferred in engineering biomimetic tissue constructs due to their intrinsic differentiation efficacy into various cell types. However, deprived efficacy based on hard-to-transfect property, in a few instances, such as embryonic stem cells (ESCs), significantly affect the cells in achieving differentiation efficiency and substantial proliferation of target tissues, which could limit their applicability in tissue engineering [379, 380]. In this context, MCM-41-type MSNs were used to design a delivery platform for efficiently monitoring the transfection of ESCs in situ [379]. The surface coating of PAH over the MSNs acted as shielding on pores, facilitating the delivery of 5*-*azacytidine (5-Aza) from MSNs, specifically at pH 5.0. The delivered therapeutic guests successfully induced/regulated their differentiation of ESCs (P19 cells) to cardiomyocytes [379]. Ren et al. fabricated ascorbic acid (AA)-loaded MSNs and subsequently demonstrated their potential for myocardial repair (Fig. 13A) [380]. Notably, MSN nanoconjugates significantly augmented the differentiation efficiency of ESCs in comparison to AA alone by substantially upregulating the expressions of various myocardial genes and subsequently downregulating the stemness genes (Fig. 13B). Although the in-vitro investigations are successful to a considerable extent, these studies warrant further feasibility toward investigations in-vivo and their substantial translation [380].

To fabricate minimally invasive surgery practice toward tissue engineering, Buchtova et al. developed an injectable cellulose derivative modified with pendant silanol or alkoxysilane groups, allowing the sol–gel pathway to reversible polycondensation, which consequently showed access to fulfill and reserve the scope of vulnerable cartilage areas [377]. The microarchitecture of the scaffold provided strong support for cell adhesion and proliferation. Interestingly, the well-interconnected porous structure of MSNs facilitated the adhesion to cells. In addition to repairing the vital organs by delivering therapeutic molecules, mesoporous silica-based materials can generate biological structures, such as bone, owing to their intrinsic bioefficacy of silanol groups, which can react with the physiological fluids, resulting in the carbonated nano-sized apatites. Subsequent delivery of antibiotics toward protection against inflammation and infection during tissue growth is another appealing, functional attribute of MSNs [383]. In a case, Trejo et al. fabricated parathyroid hormone-related protein (PTHrP)-encapsulated SBA-15-type MSNs [384]. The delivered hormone significantly promoted localized bone formation than the control groups, i.e., SBA-15 alone and media. Contrarily, it was reported that the SBA-15-type MSNs and metal-encapsulated mesoporous materials might facilitate the bone growth assisted with calcium phosphate and bone-matrix interforce enhancement. In another case, Zhu et al. utilized MSNs to co-deliver antitubercular drugs (isoniazid and rifampicin) toward long-term osteoarticular tuberculosis therapy in-vivo [385]. The MSNs-*β*-tricalcium phosphate (TCP) composites loaded with bioactive glass exhibited higher drug loading efficiencies for two kinds of drugs and excellent sustained releasing abilities for about effective concentrations over 42 days to the pure β-TCP. The long-term sustained release ability of MSNs was due to the bioactive glass coating over them, which substantially resulted in the eradication of Mycobacterium tuberculosis culture in the seeded plates in 28 days. Contrarily, the β-TCP groups exhibited the tuberculosis strain in the 14-day treatment period. Various MSN-based advanced nanocomposites with various polymeric biocompatible constructs are listed in Table 3.

**Table 3:** Several examples showing the advanced prototypes of MSNs for tissue engineering and other miscellaneous biomedical applications

Application	Advanced composites	Orientation	Particle size	Outcome	Refs
Tissue engineering	SiO_2_-NaHA/pNIPAAm	Core–shell	450–1000 nm	Injectable polymer-coated nanogels improved the stimulus–response and mechanical strength towards bioprinting	[481]
	PAH-MSNs	PAH modified over the surface	175 nm	The delivery of 5-AZA regulated the differentiation of P19 cells to cardiomyocytes	[379]
	AA-MSNs	AA-encapsulated in MSNs	94.9 ± 8.3 nm	AA delivery from MSNs induced the differentiation of human ES cells into cardiomyocytes efficiently	[380]
	MSNs	Pristine MSNs	~ 200 nm	These MSNs provided enhanced osteoblast differentiation, bone mineralization and promoted angiogenesis	[482]
	Antibiotics/antimycotics-spider silk–MSNs	MSNs-dispersed in spider silk hydrogels	MSNs- 72 nm Composite- > 2 μm	These antimicrobials-loaded MSNs provided excellent antimicrobial properties, promoting fibroblast cell adhesion and proliferation	[483]
	MSN-Dex@CS/PLLA	CS-coated MSNs in PLLA	783 ± 342 nm	Altered physicochemical and mechanical properties of PLLA offered favorable interfaces for MSCs proliferation and osteogenic differentiation	[484]
	MSNs-IGF	IGF-loaded MSNs	384 ± 134 nm	IGF delivery from biodegradable MSNs increased the cell survival rate of mesenchymal stem cells towards cardiac stem cell therapy	[480]
	D-MSNs	DMOG-loaded in mesopores	90 nm	Sustained release of DMOG from MSNs facilitated angiogenesis and osteogenesis towards bone tissue regeneration	[485]
	BMP-2 + Dex@CS-MSNs	Chitosan-coated over the surface	100–200 nm	pH-responsive release of Dex and BMP-2 from MSNs coated with chitosan stimulated osteoblast differentiation	[486]
	Eu-MSNs	Eu-doped in MSNs	280–300 nm	Eu^3+^ doped MSNs stimulated the pro-inflammatory response of macrophages and further activated osteogenic differentiation of BMSCs and angiogenic activity of HUVECs	[487]
	PA-ACP@AF-eMSN	Expanded mesopores and PA-ACP-loaded MSNs	200 nm	Polymer-stabilized intermediate precursors of calcium phosphate were delivered for intrafibrillar mineralization of collagen for in-situ mineralization of bone and teeth	[488]
	DMOG-NPSNPs	DMOG-loaded in amine-modified silicas	45 nm	These nanoporous silicas induced hypoxic conditions and promoted blood vessel formation	[489]
	HA-DMSN	Calcium-doped dendritic MSNs	> 2 μm	Increased surface area, calcium deposition, and surface roughness improved bone repair in a rat cranial bone defect model	[490]
	ADA-GEL/Ica-MSN	MSNs in hydrogels	> 2 μm	These hydrogel composites with MSNs enhanced osteoblast proliferation, adhesion, and differentiation ability for bone repair	[491]
	DBM-MSN/152RM	DBM scaffolds modified with MSNs	> 100 μm	These scaffolds promoted cell migration, differentiation, proliferation, regeneration, and angiogenesis by regulation of PTP1B	[492]
	CS-SF/MSNs/GN	Hybrid gel with silk fibroin and chitosan	> 2 μm	Well-defined injectability and thermo-responsiveness enhanced mechanically strong and elastic characteristics of the composites improved growth, matrix deposition, and osteogenic development of cells	[493]
	Ca-mSiO_2_	Ca-doped MSNs	230 nm	The MSNs as fillers presented the ability to induce mineralization, enhance mechanical properties, and prevent secondary caries	[494]
Wound healing	MSN-Ceria	Ceria nanocrystals decorated over the surface	< 100 nm	These composites offered strong tissue adhesion strength, restricted ROS in the wound, accelerated the wound healing process	[388]
	Ag-MSNs	Ag-doped MSNs	~ 200 nm	DNase I delivered from Ag-MSNs showed enhanced antibacterial effects through promoting biofilm biomass dispersing ability	[495]
	Ag-MSNs@CTAB	Janus-type	280–380 nm	pH-responsive release of antibiotics offered efficient antibacterial ability	[496]
	Fe_3_O_4_@SiO_2_@RF&PMOs	Janus-type	260 (center) + 150 nm (pods)	These tetra-pods offered high bacteria adhesion efficiency and high antibiotic loading towards treating bacterial infections	[387]
	MSNLP	β-CD-capped, PEI-modified MSNs	90–110 nm	These nanocomposites were able to load large-molecular medicine (AMPs) to eradicate pathogenic biofilms	[497]
Peptide enrichment	l-Cys-Fe_3_O_4_@mSiO_2_	Core–shell, Cys-modified over the surface	250 nm	The composites with magnetic field assistance, and strong hydrophilicity, presented highly effective against enriching endogenous glycopeptides	[498]
	Fe_3_O_4_@mSiO_2_-Cu^2+^	Core–shell, Cu^2+^ immobilized in the mesopores	350 nm	High dense Cu^2+^ ions immobilized in the mesopores improved the enrichment of hydrophobic and hydrophilic peptides from standard peptides solution	[187]
	Fe_3_O_4_@TiO_2_-ZrO_2_@mSiO_2_	Core–shell	886.5 nm	The magnetic MSNs with binary metal oxides showed enhanced enrichment performance towards mono- and multi-phospho-peptides with better sensitivity	[393]
	Ti^4+^-Mag graphene@SiO_2_	Core–shell, Ti^4+^ immobilized in the mesopores	250 nm	These composites presented great capability of fast enrichment for endogenous phosphorylated peptides	[394]
	Fe_3_O_4_@mSiO_2_-Cu^2+^	Core–shell	400 nm	Improved hydrophilic and biocompatibility of designed composites enriched the endogenous peptides from human saliva and optimized the detection of endogenous peptides	[499]
	Fe_3_O_4_@mSiO_2_@Ti^4+^-Zr^4+^	Core–shell	300 nm	The composites with more robust specificity, higher sensitivity, and better efficiency presented enrichment ability towards both mono- and multi-phosphorylated peptides	[500]
Artificial enzymes	His-BMSs, Pro-BMSs, Trp-BMSs	Amino acid-templated MSNs	150–270 nm, 130–270 nm, 150–290 nm	Comparative studies indicated that the composites with the highest wettability and the fastest degradation rate showed the lowest brain distribution ability and good biocompatibility	[501]
	EMSN-PtNCs	Pt loaded in the mesopore	200 nm	The enclosed Pt nanoclusters showed higher catalytic activity for H_2_O_2_ released from living cells	[502]
	Au-MSN	Au NPs incorporated into the pore wall	~ 1 μm	These composites presented excellent enzymatic peroxidase-like activity for determining the dopamine concentration	[503]
	T-DMSNs@Au	Au load in pores of thiolated DMSNs	162 nm	These thiolated nanocomposites with different densities of thiol groups and altered Au sizes showed significant impact with the highest sensitive peroxidase-like activity at an Au size is 1.9 nm	[504]
	PEG/Ce-Bi@DMSN	Core–shell nanorods	~ 120 nm	The nanocomposites exhibited peroxidase-mimic and catalase-mimic catalytic activities, GSH depletion, and higher hydrophilicity	[505]
	Au-MS	Janus-type	~ 100 nm	These nanodevices presented improved enzymatic processes of invertase and glucose oxidase	[506]
	PdCo@MSNs	PdCo NPs-coated over the surface	200–250 nm	These nanocomposites exhibited peroxidase-mimic and catalase-mimic catalytic activities	[507]
	Au@Pt@SiO_2_	Core–shell	~ 110 nm	The antigen-loaded composites presented susceptible peroxidase-like activity with catalytic stability and robustness	[508]
Nucleic acid detection	CaF_2_: Yb/Ho@MSNs load on TPU@GO	DNA probes-linked GO	> 2 μm	Co-hybridization between target miRNA sequences and the DNA probe enriched the accuracy of miRNA detection	[404]
	MB@MSNs–DNA	DNA-gated MSNs	~ 100 nm	DNA H1 has miRNA response, MB release, and intercalate in dsDNA enhanced significant electrochemical response	[509]
	Ca:RE^3+^@MSNs	Ca:RE^3+^ loaded in pores	~ 100 nm	Different rare earth elements provided different luminescence to detect different miRNAs	[510]
	AuNP@g-C_3_N_4_QDs@mSiO_2_	Core–shell	~ 220 nm	Enhances ECL signal and high stability towards determining Shiga toxin–generating E. Coli STEC gene	[403]
	DNA-M-PS40	Core–shell	1350 ± 50 nm	The higher surface area of the composites allowed to unequivocally detection the different high- and low-risk HPV DNA types	[511]
	DNA@MSNs	Modified over the surface	60 ± 4 nm	dsDNA open and RhB restored fluorescence for developing innovative early disease diagnosis and cell screening assay	[512]
	MSN@HRP-DNA	HRP-modified on the surface	26.3 nm	CRET, higher loading, and low CL background improved the sensitivity and selectivity of miRNA detection	[513]
	NaYF_4_:Yb,Er@NaYF_4_@mSiO_2_	Core–shell	40 nm	The designed LRET nanoprobes accurately detected target miRNA	[514]
	Large pore-MSNs	Thiol functionalized MSNs	5 μm	Large mesopores and binding sites detected short DNA sequences ~ 20 bp	[402]
	ssDNA-Au@SiO_2_	Hemispherical coat of Au on silica	450 nm	Excellent SERS effect with high sensitivity provided ultrasensitive detection of CpG methyltransferase	[515]
	Rub-Pt@mSiO_2_	Modification	380 nm	Strong ECL signal of encapsulated nanocomposites presented high sensitivity of miRNA let-7b	[516]
	Au-GNST/SiNP-IL/SPE	Loaded on electrode	~ 600 nm	Specific recognition of CTCs, with DNA probe and RCA process, generated a strong electrochemical signal	[517]
Photoluminescence	MCM-41-SH-Tb(DPA)_3_	Grafted using the chelating ligand, 4-Vinylpyridine2,6-dicarboxylic acid	> 200 nm	These lanthanides immobilized constructs presented bright green and red emission, long luminescence lifetime, high quantum efficiency	[518]
	CsPb_2_Br_5_NCs-MS	Core–shell	230 nm	These constructs offered bright emission and enhanced water stability, thermal stability, and photostability	[519]
	SiO_2_@ANA-Si-Eu-phen@SiO_2_	Core–shell-shell	200 nm	These multi-shell constructs exhibited strong luminescence properties and excellent luminescence stability	[520]
	QDs/MSF/Au	Multilayered metals on the Mica membrane	> 2 μm	Luminescence resulted in the near-infrared region with sufficient stability and low radiation loss	[521]
	R-CDs@MPS	Modification on the surface	82 nm	Reducing the aggregation-induced self-quenching of R-CDs enhanced luminescent efficiency and CRI of WLED	[522]
	CsPbX_3_@MSN	Deposited in pores	120 nm	CsPbX_3_ perovskite nanocrystals-encapsulation in MSNs offered better stability, achieving high PL quantum yield	[523]
	Er_2_O_3_@MSNs	Deposited in pores	–	The different particle sizes of the host materials have shown a biggish influence on the luminescence characters	[524]

152RM: PTP1B tyrosine-152-region mimicking peptide; 5-AZA: 5-Azacytidine; β-CD: β-Cyclodextrin; AA: Ascorbic acid; ACP: amorphous calcium phosphate; ADA-GEL: Alginate dialdehyde-gelatin; Ag-MSNs: Silver-doped MSNs; AgNPs: Silver nanoparticles; AMPs: antimicrobial peptides; ANA-Si: 5-*N*-bis(amidopropyltriethoxysilyl) nicotinic acid; Au-MSNs: Gold-incorporated MSNs; AuNPs: Gold nanoparticles; BMP-2: Bone morphogenetic protein -2; BMSs: Biomimetically-synthesized MSNs; BMSCs: Bone marrow stromal cells; Ca-mSiO_2_: Calcium-doped silica; CL: Chemiluminescence; CRET: Chemiluminescence resonance energy transfer; CS: Chitosan; CsPbX_3_: Cesium lead halide; CsPb_2_Br_5_ NCs: Cesium lead bromide perovskite nanocrystals; CTAB: Cetyl trimethyl ammonium bromide; CTCs: Circulating tumor cells; Cys: Cysteine; DBM: Demineralized bone matrix; Dex: dexamethasone; DMOG: Dimethyloxallyl glycine; DPA: pyridine-2,6-dicarboxylic acid; dsDNA: Double stranded-deoxyribose nucleic acid; ECL: Electrochemiluminescence; EMSNs: Expanded MSNs; Er_2_O_3_: Erbium(III) oxide; Eu-MSNs: Europium-doped MSNs; g-C_3_N_4_ QDs: graphite phase carbon nitride quantum dots; GN: Genipin; GNSTs: Gold nanostar structures; GO: Graphene oxide; HA-DMSN: Hydroxyapatite-dendritic MSNs; HA: Hyaluronic acid; His-BMS: C_16_-_L_-Histidine-templated BMS; HPV: Human papillomavirus; HRP: Horseradish peroxidase; HUVECs: Human umbilical vein endothelial cells; ICA: Icariin; IGF: Insulin-like growth factor; LRET: Luminescent resonance energy transfer; MB: Methylene blue; miRNA: micro ribose nucleic acid; MSCs: mesenchymal stem cells; MSNLP: Large porous MSNs; MSNs: Mesoporous silica nanoparticles; NPSNPs: Nanoporous silica nanoparticles; PA-ACP: Polyacrylic acid-stabilized amorphous calcium phosphate; PAH: Poly(allylamine hydrochloride); PEI: Polyethylenimine; PEG: Poly(ethylene glycol); PLLA: Poly-_L_-lactic acid; PMOs: periodic mesoporous organosilicas; pNIPAAm: Poly(*N*-isopropylacrylamide); Pro-BMS: C_16_-_L_-poline-templated BMS; PS: Polystyrene; PtNCs: Platinum nanoclusters; QDs: Quantum dots; R-CDs: Red carbon dots; RCA: Rolling circle amplification; RhB: Rhodamine B; RF: Resorcinol–formaldehyde resin; ROS: Reactive oxygen species; Rub: Rubrene; SERS: Surface-enhanced Raman spectroscopy; SiNPs: Silica nanoparticles; SF: Silk fibroin; SPE: Working electrode surface; STEC: Shiga toxin-generating *E. coli*; TPU: Thermoplastic polyurethane; Trp-BMS: C_16_-_L_-tryptophan-templated BMS; WLED: white light-emitting diode

In addition to metal-based materials as hard tissue replacement materials for mounting, there has been a significant interest in developing traditional ceramic replacement materials for bone tissue engineering due to their similar composition and structure to human bone. However, these ceramic substances suffer from certain drawbacks of inflexibility and fragility. These limitations can be addressed by various innovative composites based on the biodegradable and compatible polymers encapsulated with MSNs to mimic the natural bone ECM. To address these aspects, Zhou et al. generated PLGA-SBA-15 membranes based on the electrospinning approach to strengthen the adhesion and subsequent proliferation of human bone marrow-derived mesenchymal stem cells (Fig. 13C–F) [386] for promoting the activity of osteogenic differentiation. Although a few of the notified examples have exhibited the growth of tissue considering the intrinsic properties of both scaffolds and encapsulated silica-based constructs, there is still a long way for MSNs toward tissue engineering application. Moreover, in-depth analyses in understanding the behavior of tissue growth and the influence of degraded products remained to be explored. Further, these advanced prototypes of MSNs have been employed to deliver various antibacterial agents, such as AgNPs, and antibiotics, to improve the wound healing and subsequent regeneration ability of damaged skin (Table 3) [387, 388].

### Miscellaneous

Recently, in addition to drug delivery and bioimaging, these complex types of advanced MSNs have been applied in diverse advanced biomedical applications, such as peptide enrichment, photoluminescence, artificial enzymes or nanozymes, and DNA detection, among others. Owing to the exceptional physicochemical and morphological attributes, integrating MNPs offers exciting and intriguing features to this versatile MSN carrier. Herein, we give a brief synopsis and insights on the specific functional attributes of these advanced prototypes of MSNs for various innovative miscellaneous biomedical applications.

#### Peptide enrichment

**Fig. 14:**
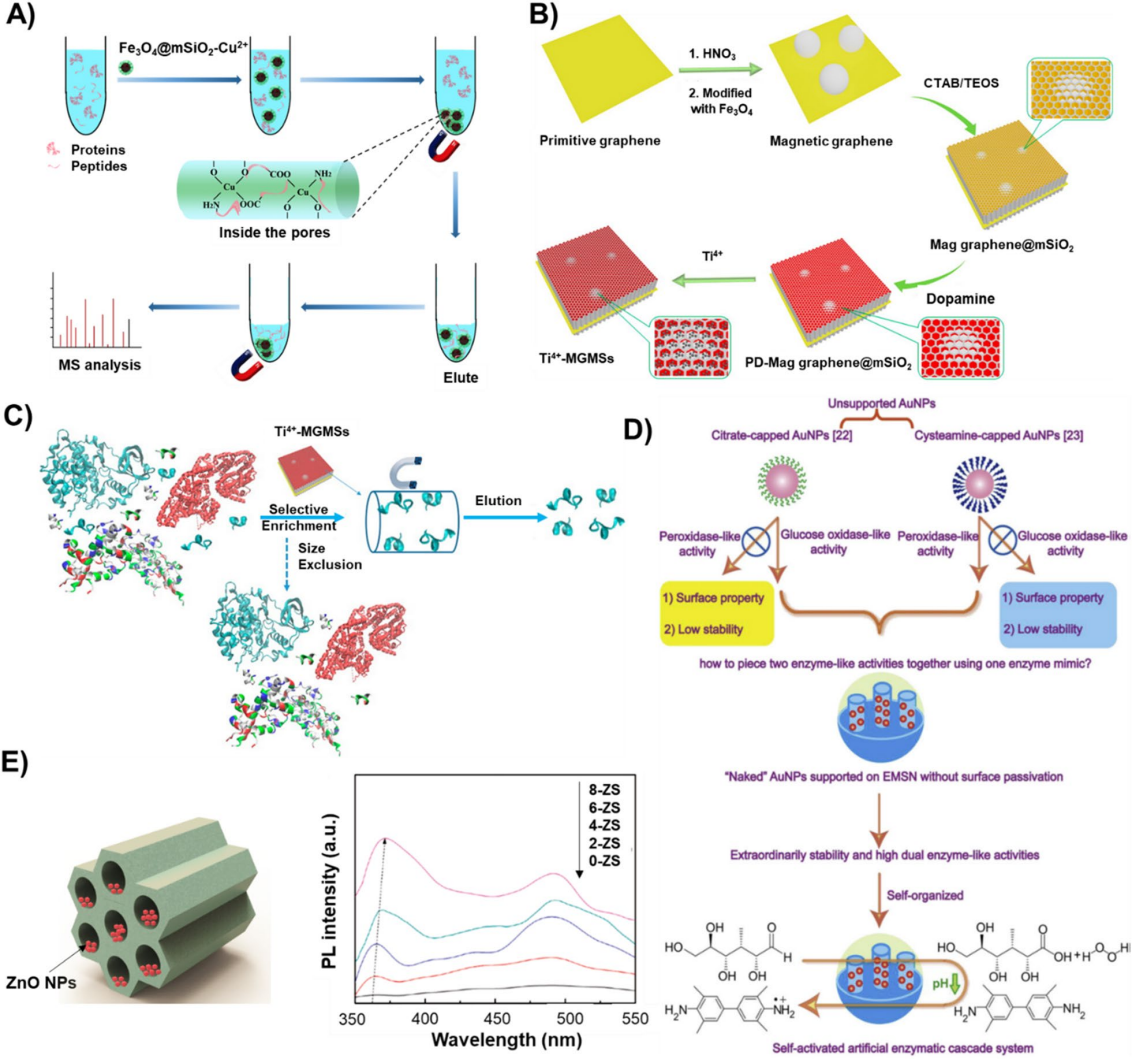
Innovative advanced metal-encapsulated mesoporous silica frameworks for miscellaneous applications. **A** Schematic representation illustrating the fast and efficient approach of selective peptide enrichment process using Fe_3_O_4_@mSiO_2_-Cu^2+^ core–shell containers and subsequent matrix-assisted laser desorption ionization-time of flight mass spectrometry (MALDI-TOF MS) analysis. Reproduced with permission from Ref. [187] Copyright 2010, John Wiley and Sons. **B**, **C** Schematic showing the synthesis of Ti^4+^-MGMSs. Reproduced with permission from Ref. [394] Copyright 2014, American Chemical Society. **D** Rational design of AuNPs-EMSN as an intelligent enzyme mimic for realizing higher functions (self-organized artificial catalytic cascade). Reproduced with permission from Ref. [395] Copyright 2013, Elsevier. **E** Schematic showing the formation of ZnO nanoparticles in the mesopores after subsequent calcination for photoluminescence (PL) applications and subsequent PL spectra at different loadings of ZnO. Reproduced with permission from Ref. [406] Copyright 2011, American Chemical Society

Currently, enormous interest has been gained by researchers in exploring the enrichment of peptides from biological samples towards safe diagnosis and other biomedical applications. Several conventional approaches have been utilized based on biomarkers for physical separation towards the quantitative and qualitative assessments of various endogenous peptides in the biological specimens (urine and blood). In this context, centrifugal ultrafiltration is the most acceptable strategy to obtain pure peptides by removing the high Mol. Wt. molecules based on the mechanism of size-exclusion [389]. To this end, MSNs have garnered enormous interest in peptidome research due to the several benefits of extensive porous channels and high surface area, facilitating the improved adsorption efficacy. These advantageous attributes of MSNs allow the efficient retaining of low molecular weight peptides from the biological samples through weak hydrophobic interactions with the interior pore walls [390–392]. Despite the success in peptide retaining ability of mesopores over other particles, such as multi-walled carbon nanotubes (MWCNTs), the stringent efficiency of peptide enrichment is still limited due to the weak hydrophobic interactions with MSN host, which may result in the escape of the adsorbed species. Combining the advancements of immobilized metal ions affinity toward chromatography technique, the typical ordered silica-based mesoporous materials exhibit sophisticated performance in proteomics via size-exclusion and magnetic field-induced separation. The advanced prototypes, metals-encapsulated MSNs (M-MSNs), could serve as potential peptide enrichment biomarkers, ensuring high safety and sensitivity in proteome research. In this framework, many endogenous peptides have been used to record the pathophysiological conditions in the body toward disease-specific diagnostic information [187]. In a case, Chen et al. fabricated iron oxide species-encapsulated MSNs with the hydrophobic surface to enhance their specificity toward hydrophobic endogenous peptides in a fast, rapid, and selective enrichment [391]. It was demonstrated that the high throughput screening of their peptide enrichment applicability was feasible through combinatorial efficacy of superparamagnetic properties of enclosed iron-oxide species and intrinsic porous architectures of MSNs [393]. Nonetheless, these composites are often preferred to enrich only hydrophobic moieties. On the other hand, they extended the research by encapsulating Cu metal ions in the nanoporous channels of the core–shell architectures to selectively improve the encapsulation of hydrophilic endogenous peptides from serum specimens efficiently (Fig. 14A) [187]. Sun et al. prepared size-selective magnetic mesoporous silica complexes with titanium (IV) immobilization steps (Fig. 14B, C), significantly simplifying the whole procedure of phosphorylated peptide enrichment [394]. Although the protein enrichment was successful in human serum and urine samples, several mechanistic elucidations are still required to be accomplished to evaluate their performance toward peptide enrichment thoroughly.

#### Artificial enzymes

The artificial enzymes often referred to as nanozymes, have emerged as promising alternatives to biological enzymes due to the specific limitations of natural biocatalysts or enzymes, such as difficulties in recovery and recycling, higher efficiency to particular substrates, high sensitivity to external conditions, and low operational stability, which limited their applicability in practical applications [395–399]. Despite the success in fabricating wide varieties of inorganic nanomaterials-based artificial enzyme-mimetic catalytic cascades, there still exist certain limitations, such as lack of potential enzyme-mimicking behavior and deprived stability attributes in terms of distribution and aggregation, which limited their performance and substantial applicability [399]. Several investigations demonstrated that the metal-impregnated MSNs could be appropriate and more convincing as nanozymes due to their intrinsic inert nature, enzyme-mimicking complexity and functional attributes, advantageous physicochemical properties, attractive catalytic, optical, and electronic properties. An exceptionally stable and self-activated, enzyme-simulated catalytic cascade system was fabricated by Lin et al. using AuNPs-encapsulated expanded MSNs (AuNPs-EMSN) (Fig. 14D) [395]. This innovative cascade system mimicked both the glucose oxidase and peroxidase-like artificial enzymes substantially. Subsequently, the AuNPs-EMSN catalytically oxidized glucose yielding gluconic acid in PBS, activating the peroxidase-like catalytic activity at reduced pH. Several advanced prototypes of MSNs utilized as artificial nanozymes are listed in Table 3. Despite the noteworthy paybacks of M-MSNs as artificial nanozymes, the applicability of advanced MSNs is still in their infant stage as the optimization experiments, and mechanistic studies in evaluating the performance as well as their subsequent advancements relevant to scalability are yet remained to be explored.

#### Nucleic acid detection

In addition to peptide enrichment, the detection of nucleic acids is one of the essential tools relevant to gene therapy and clinical diagnosis [400]. Various conventional approaches to detect DNA include DNA sequencing and polymerase chain reaction (PCR). These approaches suffer from several disadvantages, such as time-consuming and complex labeling [215, 400, 401]. To address these shortcomings, several efforts have been dedicated to the utilization of inorganic-based materials, including silica-based mesoporous materials with safe and cost-effective attributes for DNA detection. Despite the success, the applicability of conventional MSNs loaded with dyes (for instance, Rhodamine B) is limited due to poor encapsulation efficacy and unclear fundamental mechanistic studies on adsorption. In a case, Wang et al. fabricated specially designed advanced prototypes of MSNs based on Pt nanoparticles-gated MSNs (Pt@mSiO_2_) for label-free nucleic acid detection [401]. Extensive electrostatic interactions facilitated by Pt nanoparticles significantly augmented the adsorption of single-stranded DNA probes than the Pt@mSiO_2_ containers. The efficacy was confirmed by substantially preventing the intrinsic peroxidase-like catalysis of its specific substrate, TMB. Further, upon DNA hybridization in the presence of complementary DNA for detection, the Pt nanoparticles were typically available for catalysis function. In addition, the authors demonstrated that this label-free approach presented efficient, and self-signal amplifying detection, requiring no auxiliary labeling with ssDNA. Further, several efforts have been dedicated recently to exploring the applicability of DNA detection using these advanced prototypes of MSNs (Table 3) [402–404]. Despite the efficacy, in-depth analysis, and further improvements are significantly required as the fluorescence resonance energy transfer (FRET) procedure possessed similar sensitivity to that of the innovative metal-encapsulated MSNs.

#### Photoluminescence

With beneficial quantum confinement properties of metal oxides, the advanced MSNs with enclosed MNPs possess some exceptional characteristics and make them one of the most impressive materials for optical applications, such as photovoltaic sensing, photocatalysis, and diagnostics [405]. Compared to their bulk counterparts, the confined metal oxide nanoarchitectures would exhibit exceptional luminescence properties due to exceptional magnetic, electronic, and optical properties. Nonetheless, it is highly challenging to achieve control over the determination of functional attributes and altering the configuration of these remarkable materials. Moreover, they may lose their inherent luminescence property due to undesirable severe aggregation over time, which could be addressed by dispersing these photoactive nanoarchitectures into porous matrices like silica, hampering their particle–particle interactions [405]. Although MSNs exhibit specific advantages, it is convenient for these semi-conductive particles to be encapsulated in narrow-sized mesoporous channels, resulting in the enhancement of the bandgap of these semiconductor-based advanced architectures. In addition, some of these photoluminescent particles, for instance, ZnO nanoparticles, can be accumulated over the external surface of silica. To overcome this limitation, Niu et al. demonstrated the fabrication of ZnO nanoparticles-encapsulated MSNs by employing the chelating-template strategy. The zinc ions were initially captured by *N*-hexadecylethylenediamine triacetate (HED3A) and then guided the formation of mesophase, enabling the deposition of ZnO species inside the mesopores during calcination (Fig. 14E) [406]. The simplified and versatile approach could be extended to various other MNPs with excellent dispersibility, size-dependent light emission, and quantum confinement performance. In addition, these composites, including luminescent semiconductor nanoparticles and dye-doped nanocontainers, could be applicable in bioanalysis, drug delivery, and diagnosis to explore their targeting ability to specific tissues and escape from the RES uptake [407]. In this framework, luminescent bio-labeling was proposed for the first time. The concept of encapsulating transition metal-based complexes of Eu^3+^ on the surface of silica walls via a bifunctional ligand elucidated the cellular labeling under light excitation in the range of 355–365 nm [408, 409]. On the other hand, a similar type of transition complexes were impregnated in MSNs, and subsequent silica shell coating was achieved, which led to the deprived premature leakage of the encapsulated dye [408, 409].

## Steps towards scale-up and clinical translation

Despite the success in exploring the performance efficacy of these innovative advanced MSNs both in-vitro and in-vivo, there still exists a significant gap between the synthesis of advanced MSNs in lab-scale and industrial production, as well as their clinical applicability [37, 146]. Several researchers have explored the scale-up processing of these MSNs and their advanced prototypes. In a case, using mild conditions, Zhang et al. fabricated MSNs at a kilogram scale based on the templating sol–gel conditions, employing the mineralizing agent to control the morphologies through a three-step formation mechanism [410]. In another instance, Šoltys et al. generated batch synthesis of hollow mesoporous microspheres of silica using micro-emulsion-based processing with successful scale-up to 40 times [411]. In recent times, Liu et al. generated the advanced lipid-coated MSNs for irinotecan delivery at a large scale of around 100 gm, involving a multi-round optimization of various processing and formulation parameters of around 70 in number [412]. Interestingly, these lipid-coated MSNs presented an enhanced therapeutic efficacy and reduced gastrointestinal and bone marrow toxicities in a pancreatic cancer model due to excellent stability and irinotecan loading capacity. Moreover, the MSNs-based delivery platform significantly outperformed the lipid formulation (Onivyde) in clinical trials and the encapsulated pure drugs. These studies apparently provided immense confidence in researchers using silica and their conjugates towards applying them in human trials.

Despite the extensive research in the lab scale along with excellent safety and efficacy considerations, the translation rate  of silicas is lower compared to the other organic-based formulations, for instance, liposomes [413, 414]. Notably, MSNs are yet to be introduced to clinical trials due to insufficient preclinical investigations, which is the deciding factor for the clinical settings. To this end, several instances of human trials have been performed to explore their proof-of-concept of mesoporous silicas and silica-based hybrids. Owing to exemplary experimental results in the application of silica-based materials, both in-vitro and in-vivo evaluations, several instances of well-off exploration in human trials have been evidenced. For instance, dye-doped silica-based C-dots, referred to as core–shell nanosilica, have been approved by US-FDA for bioimaging applications [363, 413–415]. The interesting attributes of these dots included biological stability, non-toxicity, and high quantum effectiveness, ensuring their applicability in clinics. In another instance, nanomaterials-first-in-man (NANOM-FIM) trials of the silica-AuNPs-dispersed on-artery patch were performed [275, 379, 416, 417]. In an attempt to explore the bioavailability and reliability of silica-based formulations, a Phase-I study was conducted to assess the oral bioavailability of silica-lipid hybrids for delivering ibuprofen, confirming the efficacy of these nanostructured formulations and demonstrating their safe utilization for the delivery of poor-water soluble compounds (Ibuprofen and simvastatin) [418, 419]. In another case, a proof-of-concept in man study was performed to explore the bioavailability-enhancing potential of the ordered mesoporous silicas while delivering the fenofibrate. Interestingly, the absorption efficacy of the silica-assisted formulation was higher compared to micronized marketed formulation of fenofibrate [420]. In addition to lipid-based formulations, gold–silica hybrids were designed to execute the ultrafocal photothermal ablation of prostate cancer based on localized hyperthermia. The multiparametric MRI and postprocedure mpMRI/ultrasound targeted fusion biopsies-based treatment options presented that these hybrids were safe for men with low and intermediate risk of prostate cancers [421].

Although some efforts on clinical trials by employing silica-based formulations have been performed, it is required to elucidate various necessary parameters in terms of scale-up and comprehensive biosafety, as well as silica matrix degradability, attributes to further boost their commercialization and clinical application [4, 37, 146, 244]. Initially, essential assessments of the scale-up are required in terms of applying cheap sources and critical optimization of synthesis (processing and formulation) parameters. In this vein, several synthesis parameters of controlled reaction time, utilization of eco-friendly solvents, reduced silica density, and facile synthesis steps rather than multiple functionalization procedures must be employed. Moreover, regulating the norms and designing procedures for the systematic assessments in morphological and physicochemical attributes such as particle sizes, surface attributes would undoubtedly lead to improved industrial scale-up of these composites. Although the amorphous silica is predominantly employed in various clinical applications (for example, bone regeneration and dental applications) along with necessary evaluations, the resultant advanced MSNs are always different from their silica precursors in terms of physicochemical attributes, requiring the necessary biosafety evaluations. Moreover, several studies have reported biosafety both in-vitro in various cells and in-vivo in various animal models, requiring extensive biosafety evaluations, including but not limited to genotoxicity, immunotoxicity, and organ toxicity, due to the multiple components involved in the advanced prototypes. In addition to safety, siliceous matrix degradation is another necessary attribute for translating these innovative architectures to clinics. Notably, the degradation of the siliceous matrix is one way or the other related to the biosafety considerations of MSNs [42]. In most instances, the degradation of the rigid siliceous frameworks has been highly challenging for their clinical translation. To a considerable extent, the modified siliceous frameworks in terms of organosilicas, metal-impregnation, stimuli-responsive coordination linkages in the frameworks, and polymer-modified MSNs have presented controlled degradation [52]. For instance, the metal-impregnated MSNs, stimuli-responsive disulfide, and iron-coordinated linkages in the frameworks significantly boosted the degradation of MSNs [41, 91]. These studies demonstrated that the controlled degradation, in turn, substantially improved the biosafety of the advanced MSNs. However, the challenging aspects of the degradability of advanced MSNs include the tracking of the controllable degradation and monitoring their subsequent excretion rates, which could be supportive in exploring the toxicity attributes of these advanced MSNs. In contrast, some other advanced types resulted in no considerable degradation, resulting in undesired agglomeration and leading to thrombosis and death.

Prior to evaluating their performance and safety considerations, it should be noted that critical optimization of dosage and subsequent enumeration of PK-PD parameters are crucial to be explored with careful consideration in human subjects, as patient-specific efficacy is another challenge. In a case, Ferreira et al. fabricated ultrasmall porous silica nanoparticles to improve their pharmacokinetics towards cancer theranostics [422]. The isotopic pair ^86^Y/^90^Y encapsulated ultrasmall constructs improved the in-vivo behavior with superlong circulation by avoiding the RES uptake and excellent PET imaging with rapid and selective distribution in the targeted tissues for internal radiotherapy. Considering these aspects, particle size played a crucial role in the pharmacokinetics of the silica constructs in terms of enhanced absorption, excellent distribution, and elimination kinetics. Contrarily, in several instances, the internalization of various nanocomposites happened to be favorable in terms of surface modification with polymeric chains and targeting ligands, for instance, crossing the blood–brain barrier. To further improve the biomedical applicability of these advanced prototypes in the clinical trials, improved synthesis with optimal morphological features with simple modifications, as well as detailed toxicity along with PK-PD profiles, and comprehensive biological assessment tests of the notified parameters are necessary to be evaluated in human patients, requiring various in-vivo tests for biosafety evaluation initially and subsequent performance efficacy measurements of these MSNs and their advanced prototypes.

## Conclusions and outlook

This review has provided a critical emphasis on the fabrication and biomedical applications of multifunctional advanced MSNs, in terms of different modified surfaces, engineered frameworks, altered porosity, and specially designed architectures with improved functionalities. Initially, we have provided a brief explanation of synthesizing the MSNs and the critical emphasis of the possible factors, both formulation and processing points-of-view. Further, engineered MSNs with different modifications concerning modified surfaces, altered porosity, engineered frameworks, and various innovative and complex architectures are emphasized, highlighting their pros and cons towards improving the physicochemical and morphological attributes. By integrating the realms of chemistry, physics, and medicine, these innovative prototypes of MSNs have opened up new burgeoning opportunities towards diverse biomedical applications. Furthermore, we provided several exciting discussions in the application of these advanced MSNs towards diverse, innovative stimuli-responsive (pH/light/thermos/ultrasound)-based drug delivery strategies and imaging, as well as artificial enzymes, peptide enrichment, photoluminescence, and biosensing, among others, highlighting their structural and physicochemical attributes in biomedical applications. Finally, discussions on the critical necessities and progress in translating these innovative architectures to clinics are provided.

Although several efforts have been dedicated to exploring various progressions in the generation and applicability of these innovative MSNs, there still exist some challenges in terms of fabrication and performance attributes towards their adequate translation and commercialization. The astonishing gap between the current progress in lab-scale and future advancement towards their applicability and commercialization lies in various factors, such as biosafety, colloidal stability, and degradability regarding performance attributes and scale-up in terms of processing [412]. In terms of performance, the safety of these advanced MSNs at a comprehensive scale has yet remained to be addressed. Although some investigations in-vitro in cell models and in-vivo in animal models have been performed, comprehensive toxicity evaluations with detailed insights on nephrotoxicity, immunogenicity, and others have yet remained to be addressed. These studies, in several instances, have been studied for various silica-based platforms, including conventional MSNs [423–427]. However, the changes in the fabrication procedures, altered morphologies of nanoplatforms, in this case, modified surfaces and siliceous frameworks through impregnating various conjugates, may alter their safety profile, requiring extensive biosafety investigations at all the levels of preclinical stages. Second, depending on the size, shape, and surface chemistry, the fate of the designed formulation is correlated to the success of the delivery system. Among such various features, colloidal stability of the nanoformulation plays a crucial role in the dosage distribution and their fate in the systemic circulation, delivery patterns, and eventual clearance [294, 425, 428, 429]. MSNs are generally negative in charge and stable in physiological fluids, which has limited their successful delivery as the biological membranes possess negative charge, resulting in repulsion and poor delivery. However, this limitation has been overcome by modifying the surfaces with polymers and siliceous frameworks with transition metals towards improving colloidal stability. In addition to such changes, it is required to address the aggregation phenomena through stringently controlling the eventual particle size of these advanced prototypes and optimizing the surface charge for appropriate application as a paramount interest with a necessity in avoiding multiple functionalizations’. Third, the controlled degradability of the advanced prototypes of MSNs must be considered, besides safety. Notably, these two features are one way or around related in biomedical applications, which are often referred to as essential prerequisites as non-degradable materials would often lead to accumulation-induced toxicity risk. To a considerable extent, the modified frameworks of MSNs (PMOs and metal-impregnated MSNs) and degradable polymers-coated MSNs led to change in their degradability profiles. Moreover, strict regulation of safety procedures and fundamental concepts for establishing rapid degradability profiles with standard operations and guidelines must be established. Similarly, we are anticipating various modifications in the MSNs that will undoubtedly improve the degradability and safety attributes, alleviating the chances for clinical translation.

Since their inception, several reports have demonstrated the scalability and reproducibility of conventional MSNs. As the lab scale is different from the industrial scale in processing, several predominant reasons include the batch-to-batch challenges relevant to reproducibility maintaining uniform-size distribution, encapsulation efficacy in high yields, and non-availability of cheap silica sources, resulting in increased cost. These challenges always hindered the adoption of these materials. Concerning the advanced prototypes, it is still a long way to go towards the scale-up of these innovative prototypes, requiring the facile synthesis procedures, cheap silica sources, reduced fabrication steps, saving time of production. However, the rapid pace of research and steps towards translation would certainly address these challenges and see the advanced prototypes in the future.

## Data Availability

All relevant figures and tables are included in this manuscript.
